# Design and analysis heterogeneity in observational studies of COVID-19 booster effectiveness: A review and case study

**DOI:** 10.1126/sciadv.adj3747

**Published:** 2023-12-20

**Authors:** Sabir Meah, Xu Shi, Lars G. Fritsche, Maxwell Salvatore, Abram Wagner, Emily T. Martin, Bhramar Mukherjee

**Affiliations:** ^1^ Department of Biostatistics, University of Michigan School of Public Health, Ann Arbor, MI 48109, USA.; ^2^ Department of Urology, Michigan Medicine, Ann Arbor, MI 48109, USA.; ^3^ Center for Precision Health Data Science, University of Michigan, Ann Arbor, MI 48109, USA.; ^4^ Rogel Cancer Center, University of Michigan, Ann Arbor, MI 48109, USA.; ^5^ Center for Statistical Genetics, University of Michigan School of Public Health, Ann Arbor, MI 48109, USA.; ^6^ Department of Epidemiology, University of Michigan School of Public Health, Ann Arbor, MI 48109, USA.; ^7^ MRC Biostatistics Unit, University of Cambridge, Cambridge, UK.

## Abstract

We investigated the design and analysis of observational booster vaccine effectiveness (VE) studies by performing a scoping review of booster VE literature with a focus on study design and analytic choices. We then applied 20 different approaches, including those found in the literature, to a single dataset from Michigan Medicine. We identified 80 studies in our review, including over 150 million observations in total. We found that while protection against infection is variable and dependent on several factors including the study population and time period, both monovalent boosters and particularly the bivalent booster offer strong protection against severe COVID-19. In addition, VE analyses with a severe disease outcome (hospitalization, intensive care unit admission, or death) appear to be more robust to design and analytic choices than an infection endpoint. In terms of design choices, we found that test-negative designs and their variants may offer advantages in statistical efficiency compared to cohort designs.

## Teaser

Boosters offer robust protection against COVID-19 hospitalization and death.

## INTRODUCTION

The first emergency use authorization (EUA) of vaccines for coronavirus disease 2019 (COVID-19) in the US in December 2020 ushered in a new era of the pandemic, with mRNA vaccines having shown strong efficacy against both confirmed symptomatic disease and severe outcomes in randomized clinical trials (*1*). After the EUA and broad administration of the vaccines, observational studies sought to measure real-world vaccine effectiveness (VE) against infection and severe disease outcomes such as hospitalization and death. Many early observational studies reported strong effectiveness against both infection and severe disease, consistent with the clinical trials (*2*).

However, in late 2021, early data on waning immunity motivated officials in the US and other countries to approve booster doses of COVID-19 vaccines (*3*–*5*). The landscape has further evolved with the approval and administration of additional booster doses (*6*), emergence of new and more immune-evasive severe acute respiratory syndrome coronavirus 2 (SARS-CoV-2) variants (*6*–*8*), bivalent boosters updated for such new variants (*9*–*11*), and general increase in vaccine availability worldwide (*12*, *13*). In the US, as of 10 May 2023, 92.3% of the adult population has received at least one dose of a COVID-19 vaccine, while 20.5% of the adult population has received an updated bivalent booster (*14*). In addition, the US Centers for Disease Control and Prevention (CDC) has recommended another updated COVID-19 vaccine in September 2023 (*15*). This dynamically evolving environment of vaccine-conferred immunity has led to an exploding number of papers and preprints on VE in 2022. Our sweep of all VE literature identified 380 papers and preprints in 2022, compared to 77 in 2021.

However, reported VEs for the first booster versus primary series vaccination in such studies have varied considerably, particularly for infection, where estimates from as high as 93% to as low as negative effectiveness can be found (*16*–*18*). Differing epidemiological and clinical landscapes, including preexisting natural immunity, evolution of COVID treatments, and contemporary variant circulation, may explain some of the differences between study results, but there still remains substantial heterogeneity in VE estimates reported by studies in the same country at around the same time, particularly in the US (*19*, *20*).

Another factor that may explain some of the substantial differences in the results of VE studies is heterogeneity in study design and statistical methods. In a systematic review and meta-analysis of VE studies for primary series vaccination, Zeng *et al.* (*21*) noted that “there is high heterogeneity between studies, and high statistical heterogeneity is also observed in most analysis.” The same has been observed in booster VE studies regarding study design and statistical methods.

Most observational designs used in booster VE studies can be classified into two categories: cohort designs, where a study population is (identified retrospectively or prospectively) followed over time to see whether they develop the outcome of interest (*22*–*24*), and test-negative designs, where a study population is constructed from those who present for COVID-19 testing with similar symptom manifestations, with cases defined as those who test positive and controls as those who test negative (*25*–*27*). The test-negative design is a particular form of case-control study where the cases and controls are ascertained through testing. This reduces potential confounding due to health care–seeking behavior that may affect both the outcome (infection status) and exposure (vaccination status) (*28*). In addition, there are other case-control designs, where cases are defined as those presenting a certain outcome, such as infection or hospitalization, and controls are chosen in various different ways, such as hospital- or population-based controls (*29*, *30*).

**Fig. 1.**
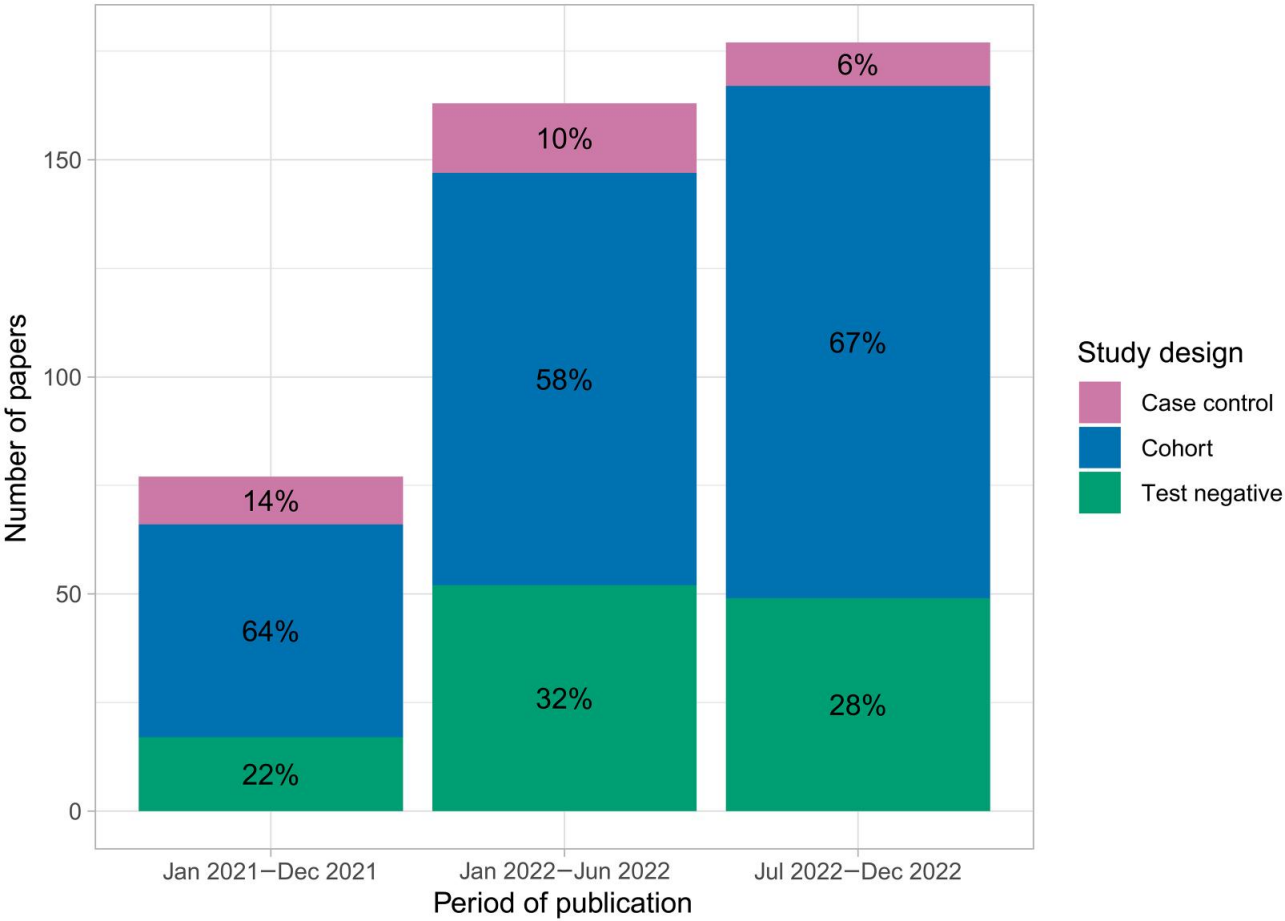
Summary of literature sweep of all COVID-19 VE literature in 2021–2022. Number of published COVID-19 VE articles, preprints, or conference abstracts in each of three time periods: 2021 (January 2021 to December 2021), first half of 2022 (January 2022 to June 2022), and second half of 2022 (July 2022 to December 2022). We do not divide 2021 in halves given the low volume of literature in the first half of 2021 (an alternative figure with both years divided into halves is given in fig. S39). Bars representing each time period are colored by class of study design, with the percentage of literature having each design in that time period displayed inside each shaded area.

To illustrate this, we see in the latter half of 2022, of 177 papers identified in a sweep of all VE literature, 49 (28%) used a test-negative design, 118 (67%) used a cohort design, and 10 (6%) used other case-control designs (Fig. 1). In addition to differences in study design, studies made different choices regarding adjustment for confounding, stratification, and other aspects of statistical analysis. The effect of these choices on the ultimate VE estimates for infection, hospitalization, and death is not clear.

In this study, we attempt to analyze study design and analysis heterogeneity for COVID-19 booster VE studies using a two-step process. First, we conduct a literature review of studies estimating booster VE relative to primary series vaccination or second booster VE relative to first booster vaccination, summarizing VE estimates and methodology found in current work. We also report relative (to any previous number of boosters) VE estimates from early studies of the bivalent booster. Second, we implement 20 different methods for estimating VE on electronic health record (EHR) data from Michigan Medicine (MM). This includes the methods commonly found in the literature, methods found occasionally in the literature, and additional methods not found in the literature that we believe may be useful. This case study demonstrates how study design and analysis decisions can influence reported VE estimates and results even when we use the same data source and time period.

## RESULTS

### Literature review

#### Included studies

Three hundred twenty-four records, including 217 from PubMed, 95 from Embase, and 12 from other sources including Google Scholar, were identified through the database search. Sixty-nine satisfied our inclusion criteria, with 53 of them estimating VE for the first booster and 16 for the second booster. Figure S1 displays the PRISMA flow diagram describing the process of article selection. Tables S1 to S3 present summaries of the types of study designs adopted in the included studies. Of the 69 included studies, 17 used a test-negative design (4 with matching), 2 used a non–test-negative case-control design (none with matching), and 50 used a cohort design (19 with matching). The proportion of cohort designs was higher for second booster studies compared to first booster studies (81% versus 70%, respectively). The 69 studies in the literature review included 129,368,483 individuals (or tests in designs where tests, not individuals, were the unit of observation) in total.

Note that the scope of our literature review was limited to studies that measured VE relative to the previous stage of vaccination, termed rVE by some studies, and as a result, any reference we make to VE is relative to the previous vaccination. Studies that measured absolute VE (relative to no vaccination, i.e., an unvaccinated reference group) were not included in our literature review.

#### First booster VE against infection

**Fig. 2.**
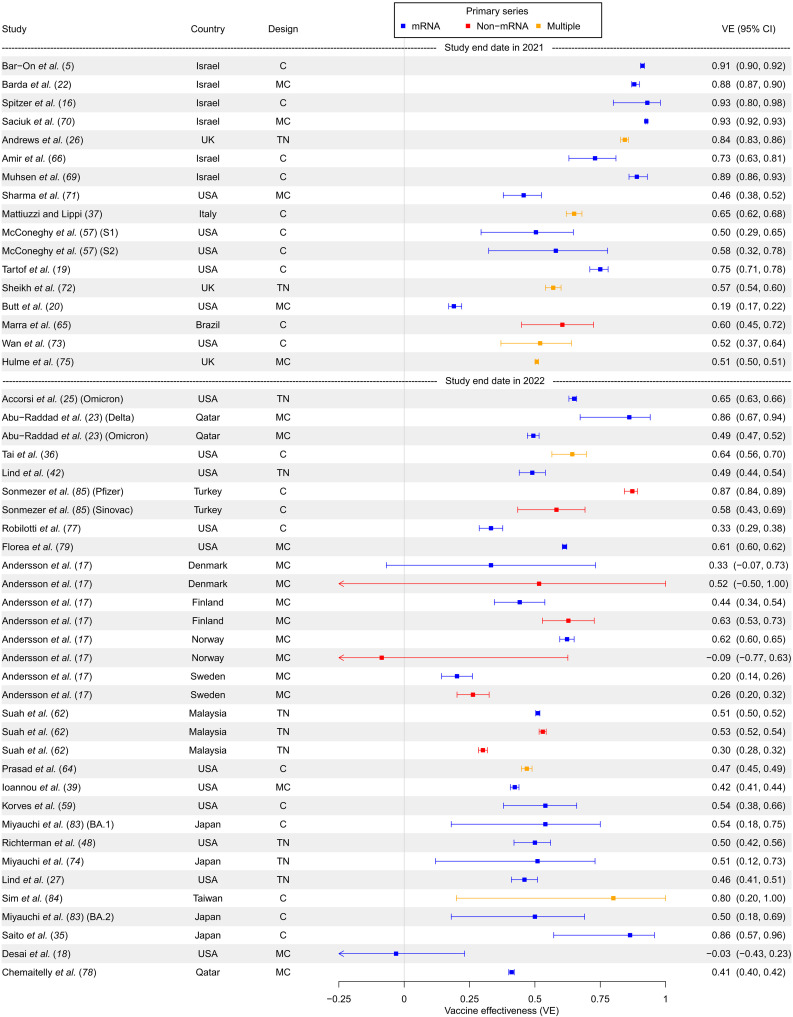
First booster versus primary series VE against infection (in studies unstratified by time since booster) First booster VE compared to primary series against infection, in studies unstratified by time since booster administration. Studies are sorted in chronological order of last month included in the study period (with publication date being used to break ties). Year of study end date (noting that the end of 2021 was a time approximately corresponding to the dominance of the Omicron variant in many countries) is denoted by dashed lines in the figure, with studies completed after the year start below the respective year marker. TN, test-negative; MTN, matched test-negative; CC, case-control (non–test-negative); MCC, matched case-control (non–test-negative); C, cohort; MC, matched cohort; S1/S2, system number for McConeghy *et al.* (*57*).

A forest plot summarizing first booster versus primary series VE against infection for studies not stratified on time since booster can be found in Fig. 2, while VE stratified by 2 weeks and 1, 2, and 3 months since booster administration can be found in figs. S2 and S3.

Infection VE for the first booster was high (80 to 95%) for the earliest published studies in 2021 but then dropped substantially for studies published in 2022 (to around 50%, and several studies reported even lower VEs around 30%) (Fig. 2). We noted consistent patterns over time in studies that presented data stratified by time since vaccination (figs. S2 and S3). A clear pattern of waning as time since vaccination increased can also be observed. Infection VE 1 month after vaccination ranged from 41 to 67% in 2022 studies and then (aside one outlier at 69%) fell to −4 to 44% (with most studies around 30%) 3 months after infection (fig. S3).

Cohort designs, including both with and without matching, were the majority class of study design (Fig. 2). Test-negative designs, however, appeared to be popular for earlier studies stratifying on time since vaccination (figs. S2 and S3). The earliest studies were predominantly from Israel, followed by studies in the US and western Europe (particularly the UK), then followed by studies from Asia (although the US contributed many later studies as well). Perhaps related to the timing of studies, Israeli studies for the most part exhibited similar VE, while studies from the US or Europe displayed a high degree of heterogeneity of VE estimates.

There were no clear differences in booster VE when comparing study populations with mRNA or non-mRNA primary series vaccines (Fig. 2). However, studies comparing the first booster to a non-mRNA primary series vaccination sometimes exhibited slightly lower VE compared to studies using an mRNA primary series, when from around the same time and when stratified by time since vaccination (figs. S2 and S3).

#### First booster VE against symptomatic infection

Of the 50 first booster VE studies we reviewed, 11 considered symptomatic infection. Symptomatic infection VE (fig. S4) followed a similar pattern as any infection VE, with the earlier 2021 studies exhibiting very high VE (84 to 91%). Studies in later 2021 and 2022 had lower VE (49 to 65%). These numbers were higher and less variable across studies than VE for any infection.

Forest plots for the symptomatic infection endpoint stratified by time since booster administration can be found in fig. S5, although this only includes four studies. The two 2021 studies reported high VE (78 to 86%) in every time strata. The two 2022 studies reported sharply declining VE across time strata (54% 1 month since booster administration and −4 to 25% 3 months since booster administration), a pattern consistent with any infection VE. Additional patterns in terms of study design and type of primary series vaccination were similar to patterns noted with any infection VE.

#### First booster VE against hospitalization/severe disease or death

**Fig. 3.**
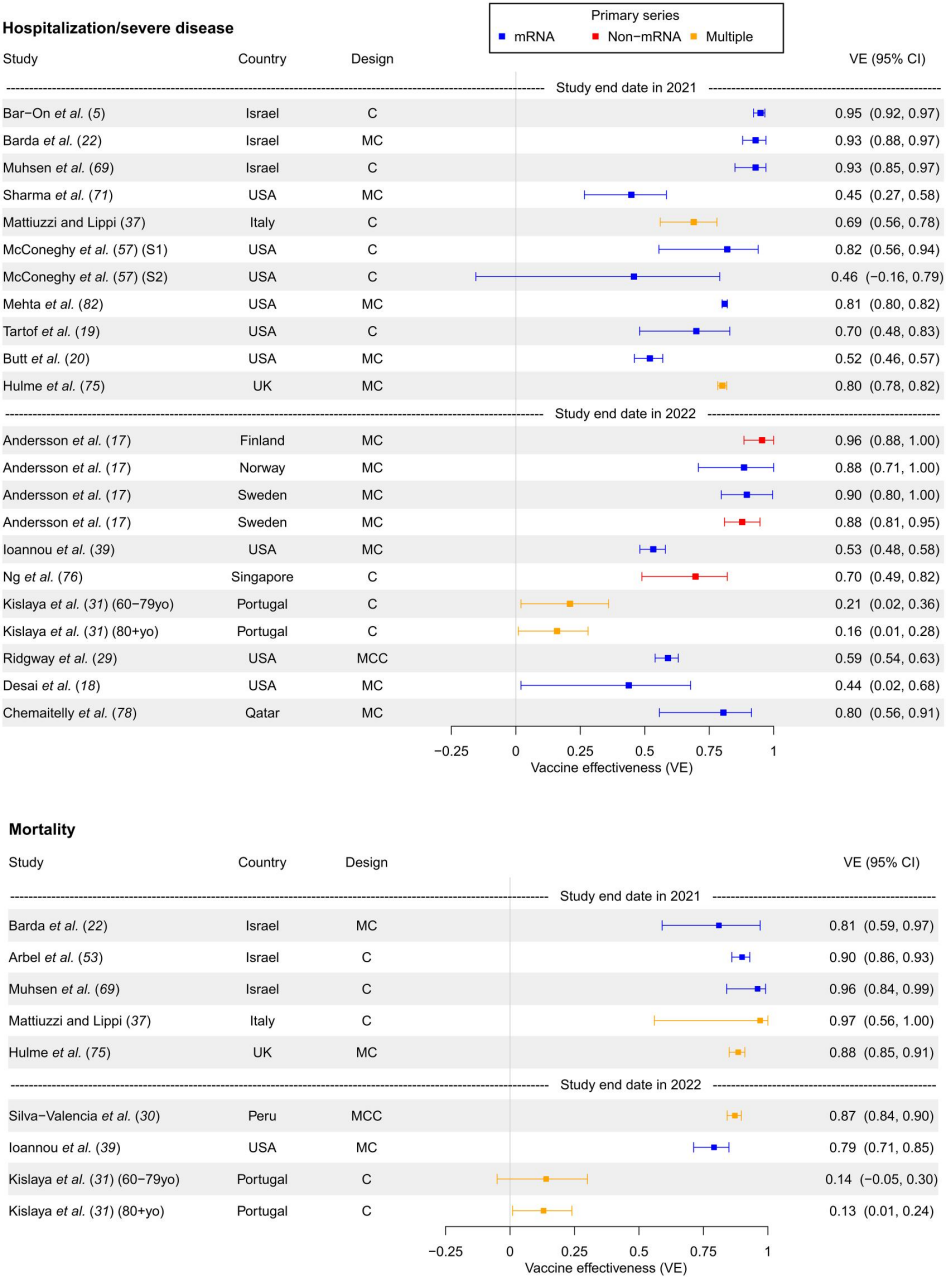
First booster versus primary series VE against hospitalization/severe disease and mortality (in studies unstratified by time since booster). First booster VE compared to primary series against hospitalization or severe disease outcomes and mortality in separate panels, in studies unstratified by time since booster administration. Studies are sorted in chronological order of last month included in the study period (with publication date being used to break ties). Year of study end date (noting that the end of 2021 was a time approximately corresponding to the dominance of the Omicron variant in many countries) is denoted by dashed lines in the figure, with studies completed after the year start below the respective year marker.

A forest plot summarizing first booster versus primary series VE against hospitalization or severe disease and against death can be found in Fig. 3, while VE stratified by 1, 2, and 3 months since booster administration can be found in fig. S6 for hospitalization/severe disease and in fig. S7 for death.

Similar to infection VE, early studies examining VE against hospitalization or severe disease outcomes exhibited very high VE (81 to 97%), particularly the earliest Israeli studies (Fig. 3 and fig. S6). However, the drop in VE in later studies was not nearly as precipitous [with just Kislaya *et al.* (*31*) outlying], declining to around 44 to 80%. It also occurred later than with infection, in mid-2022 (Fig. 3 and fig. S6). Similarly, VE against mortality was high in early studies (80 to 90%) and dropped only slightly [again apart from Kislaya *et al.* (*31*)] in later studies to 62 to 89% (Fig. 3 and fig. S7).

The effect of waning immunity over the 3 months since vaccination for the outcomes of severe disease and death was much less than that for infection (figs. S6 and S7). VE against hospitalization ranged from 64 to 91% 1 month after vaccination in 2022 studies and from 48 to 79% 3 months after vaccination in studies also ending in 2022.

The cohort study design was far more dominant in hospitalization/severe disease or mortality VE studies than infection studies (Fig. 3). There were only two mortality studies that used a noncohort design: one case-control and one test-negative (Fig. 3 and fig. S7).

Like infection VE, hospitalization/severe disease studies did not display a clear divide in VE estimates between mRNA and non-mRNA primary series. For mortality outcomes, many studies used a mixed selection of primary series vaccinations, so it was impossible to disentangle differences in VE across these subgroups.

#### Second booster VE

**Fig. 4.**
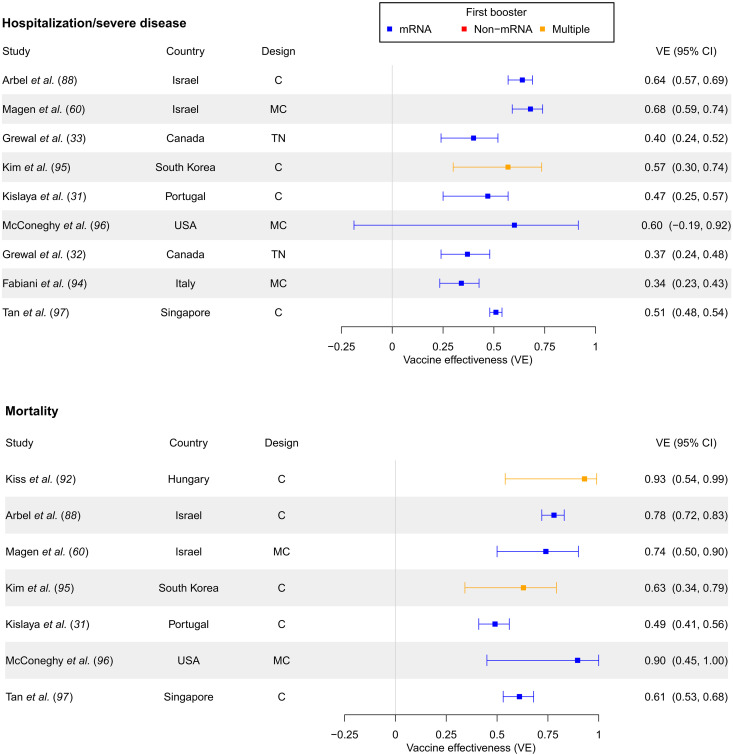
Second booster versus first booster VE against hospitalization/severe disease and mortality (in studies unstratified by time since booster). Second booster VE compared to primary series against hospitalization or severe disease outcomes and mortality in separate panels, in studies unstratified by time since booster administration. Studies are sorted in chronological order of last month included in the study period (with publication date being used to break ties).

Forest plots summarizing second booster versus first booster VE against hospitalization/severe disease and mortality for studies can be found in Fig. 4. An additional plot for second booster VE against infection can be found in fig. S8. Forest plots summarizing second booster VE stratified by time since vaccination are not included on account of the small number of studies for each time stratum.

Similar to first booster results, second booster versus first booster VE was higher for the early set of studies (30 to 61% for infection, 40 to 68% for hospitalization/severe disease, and 74 to 93% for death), often in Israel, but subsequently dropped for later studies in other settings (Fig. 4 and fig. S8). Overall, relative VE estimates across the board appeared to be lower for the second booster. The decline through later study dates was still more pronounced for infection than for hospitalization/severe disease and mortality. Infection VE in later studies fell to 14 to 31% (fig. S8). VE against hospitalization after the early Israeli studies was in the range of 34 to 60% (Fig. 4). VE against death ranged from 49 to 90%, with more later studies being closer to the lower bound (Fig. 4).

Of the 13 second booster studies examining infection, 5 provided VE estimates for symptomatic infection. A forest plot showing second versus first booster VE for only symptomatic infection can be found in fig. S9. Symptomatic infection VE ranged from 19 to 55%, with some evidence of a decline over time. Four of these studies measured VE against both symptomatic and any infection, and symptomatic infection VE estimates were typically higher than any infection VE by 10 to 13% (estimated differences of 13, 10, 12, and 13%).

Cohort designs were dominant for second booster studies. Every second booster mortality study in the review used a cohort design, and the test-negative design was used only by Grewal *et al.* (*32*, *33*) for infection and severe disease outcomes. Most second booster studies estimating infection VE used matching, although several hospitalization/severe disease studies and most mortality designs did not use matching. There were no easily discernible patterns regarding first booster type (mRNA versus non-mRNA).

#### Bivalent booster VE

**Fig. 5.**
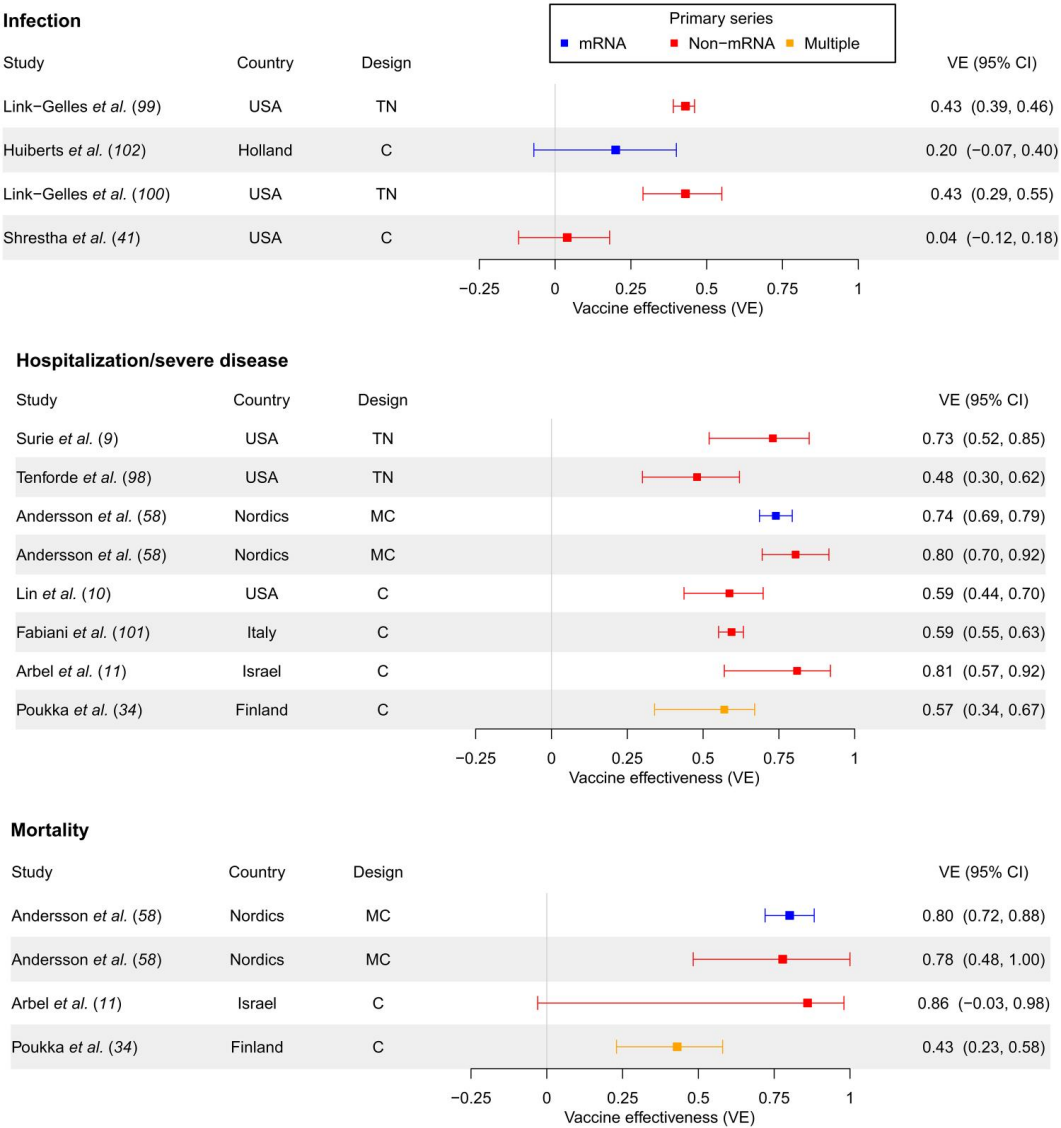
Bivalent booster (targeting either BA.1 or BA.4/5) VE compared to a previous dose against infection, hospitalization/severe disease, and mortality only in separate panels. Studies are sorted in chronological order of last month included in the study period (with publication date being used to break ties). For studies with stratified results, we displayed results corresponding to only the oldest age, most recent variant, longest time since previous dose, and a 2-month time since boosted strata. For Andersson *et al.* (*58*), the Nordic countries included were Denmark, Finland, Norway, and Sweden (Iceland and Greenland were not included).

Our Google Scholar query for bivalent booster VE (relative to any number of prior doses) returned 853 articles, and we screened the first 40 of them (in addition to another article identified through the citations of these articles). We ultimately included 11 articles (including 4 CDC *Morbidity and Mortality Weekly Reports*) generating bivalent booster VE estimates. Four of the studies used a test-negative design (none using matching), and seven used a cohort design (one using matching). Full study characteristics can be found in table S4, and a forest plot summarizing the estimates can be found in Fig. 5. The 11 studies included 23,215,145 individuals (or tests in designs where tests, not individuals, were the unit of observation) in total.

VE against any infection was unclear, with two cohort studies failing to find a VE significantly different from zero, and two test-negative studies finding a VE of 43%. Notably, the latter two studies measured only symptomatic infection, while the former two included any infection. VE against hospitalization/severe disease, however, was much higher, in the range of 48 to 81%. VE against death was close to 80% in most studies, but was also characterized by wide confidence intervals (CIs) because of limited follow-up data.

We additionally provide forest plots (figs. S10 and S11) stratifying estimates based on age above or below 65 years (or a proximal cut point such as 60 years, which some European studies used). Because of differences in access, availability, eligibility, and uptake of bivalent boosters, only studies from the US and one study from the Netherlands were able to evaluate VE in individuals younger than 65/60 years, and most of these studies only reported infection VE, given the rare incidence of COVID-19 hospitalization and death in younger populations. Each of the three studies that evaluated bivalent booster infection VE in both those above and below 65 years of age reported slightly lower VE for those 65 and older than those younger (around 10 percentage points lower in each of these three studies: 43% versus 56%, 20% versus 31%, and 43% versus 49%).

### MM data analysis

#### Study population

The MM EHR cohort corresponding to the main analysis (first booster versus primary series) included 186,495 individuals overall, with 153,811 boosted before or during Q4 2021, and 32,684 fully vaccinated but not boosted before the end of Q4 2021 (i.e., eligible to be included in the comparative primary series vaccination group). A full set of descriptive statistics for the cohort can be found in table S5. Boosted individuals were generally older (57.0 versus 47.6 average age for boosted and fully vaccinated, respectively) and had a higher percentage of non-Hispanic whites (77.5% versus 68.6%) than individuals not boosted by Q4 2021. In addition, they had more comorbidities (1.01 versus 0.87 average Charlson comorbidity index) and were, on average, from less affluent neighborhoods (47.1% versus 38.7% of individuals in the lowest quartile). Both groups were mostly female (by self-reporting), but boosted individuals were a higher proportion male (42.9% versus 39.9%). Rates of working in health care (1.6% versus 2.3%) and number of negative tests (1.35 versus 1.47 average) were slightly lower for boosted than fully vaccinated individuals. There were not notable differences between the boosted and fully vaccinated in body mass index (BMI) (28.7 versus 28.9 average), population density of residence area (24.5% versus 25.0% in least dense quartile), and proportion receiving primary care at MM (51.2% versus 51.6%). Study population characteristics for the test-negative analysis are given in table S6 and display similar patterns. Study population characteristics for the two or more versus one booster cohort and the full cohort of fully vaccinated individuals no matter the number of boosters are given in tables S7 and S8.

In the cohort analysis, 7.77% of boosted and 6.55% of fully vaccinated individuals had a positive test in the follow-up period, while 0.40% of boosted and 0.38% of fully vaccinated individuals had severe disease. In total, 5.21% of test-positives (of any vaccination status) developed severe disease.

#### Estimated VE

**Fig. 6.**
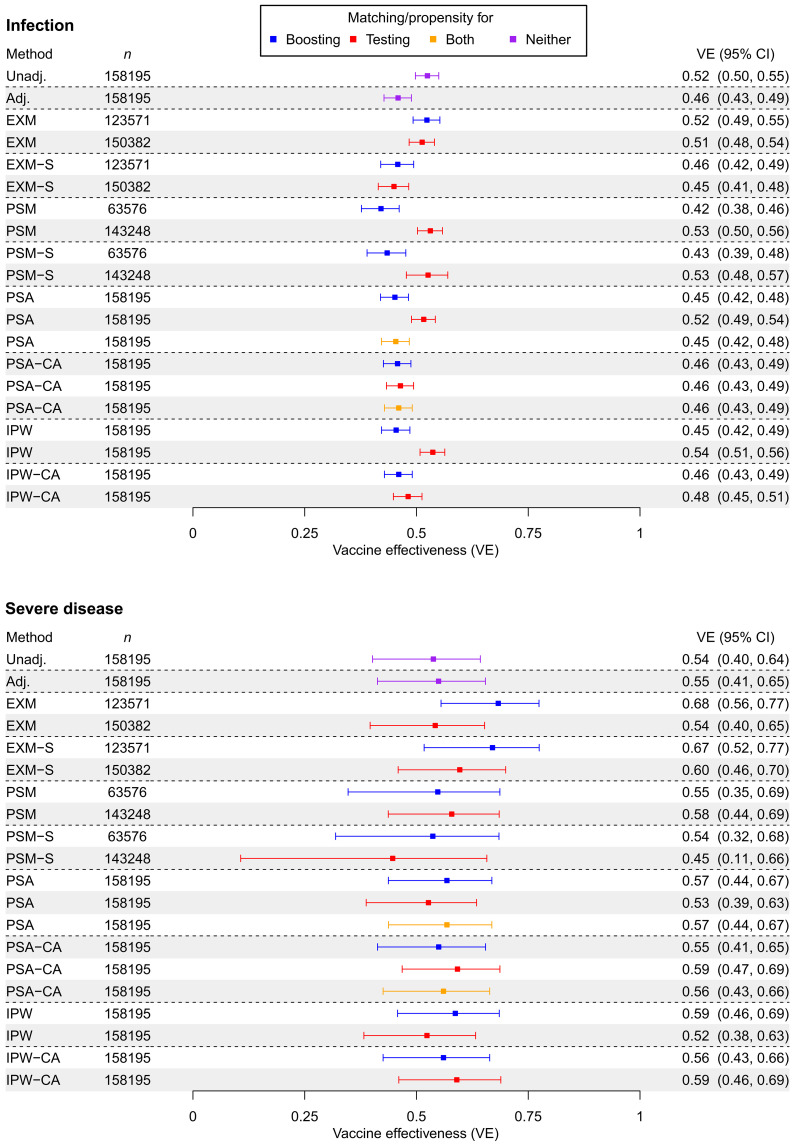
First booster/third dose versus primary series/second dose VE against infection (first panel) and severe disease (second panel). We apply various matching designs during a study period of 1 October 2021 to 31 December 2022. Methods not using matching/propensity scores (purple) and methods using a matching/propensity scores for boosting (blue) were present in the literature. We added methods using matching/propensity scores for testing (red) or both propensity scores for boosting and testing (orange) to the comparison to explore to what extent adjusting for selection bias due to testing matters in determining VE estimates. Unadj., unadjusted; Adj., adjusted for covariates; EXM, exact matching; EXM-S, exact matching with matching strata stratification in Cox regression; PSM, propensity score matching with a 0.2 caliper; PSM-S, propensity score matching with a 0.2 caliper and matching strata stratification in Cox regression; PSA, adjustment for propensity score; PSA-CA, adjustment for propensity score and for covariates; IPW, inverse probability weighting of propensity score; IPW-CA, inverse probability weighting of propensity score also adjusting for covariates.

Relative VE estimates obtained by applying different statistical methodologies for infection and severe disease outcomes using the cohort design are shown in Fig. 6. Associated Kaplan-Meier curves can be found in fig. S12. A summary of the propensity score models can be found in table S9. Results using different study start dates can be found in figs. S13 and S14. Corresponding results using the test-negative design can be found in figs. S15 to S18.

Point estimates of infection VE ranged from about 42 to 54% across different statistical methods (Fig. 6). We note that either regression adjustment for the full set of covariates or accounting (with either matching, weighting, or adjustment) for propensity of receiving a booster led to more conservative VE estimates (42 to 48%). Solely adjusting, matching, or weighting for propensity of testing led to higher VE estimates compared to other methods (52 to 54%). For any matching strategy, stratification of the Cox proportional hazards model led to lower VE estimates (43 to 53%). Matching, in general, led to wider CIs for VE due to loss of observations.

VE estimates for severe disease fell in a range from about 45 to 68%, but generally had much wider CIs than infection VE because of a limited number of events (Fig. 6). Excluding matching methods (i.e., methods that discard observations), however, VE ranged only from 53 to 59%. Matching methods in general had wider CIs, as with infection VE. All CIs for severe disease VE estimates across the different methods were largely overlapping.

The test-negative analysis (figs. S15 and S17) demonstrated a similar pattern: VE estimates against infection ranged from 40 to 57%, while VE against severe disease, aside from some erratic results with exact matching, ranged from 65 to 75%. With the test-negative results for the infection outcome, there were two clusters of VE estimates: one for methods that involved covariate adjustment (ranging from 53 to 57%) and another for methods that did not (ranging from 41 to 47%). However, one noteworthy observation was the reduction in the width of the CI by using a test-negative design, particularly when using inverse probability weighting (IPW) (by the propensity of boosting) and covariate adjustment. While the severity VE for the cohort design corresponding to the same method was 56% (95% CI: 43 to 66%), the test-negative design VE was 67% (95% CI: 62 to 72%), a much narrower CI.

A necessary consideration for the extension of the test-negative design to estimate severity VE is to consider case-control studies where cases are defined as severe COVID-19 [hospitalization/intensive care unit (ICU) admission], and the test results may inform the ascertainment/definition of the control group (*28*). Within the same method, we observed only slight variation in point estimates depending upon the choice of the control group, which could be one of test-negatives, those without severe disease regardless of test results, and test-positives without severe disease. In all methods except exact matching, where small sample sizes produced erratic results, VE estimates from using each of the three control groups all were within a six-percentage point interval or smaller (fig. S17).

Results for the following secondary study populations can be found in the Supplementary Materials. Both cohort and test-negative results for the vaccination group of one or more boosters (versus primary series) can be found in figs. S19 to S28, while cohort and test-negative results for the vaccination group of two or more boosters (versus one booster) can be found in figs. S29 to S38.

## DISCUSSION

### Literature review

#### Choice of boosters: VE of first, second, and bivalent boosters

##### First booster

We found that the preponderance of the literature suggests that, relative to a primary series vaccination, a first booster provides strong protection against both hospitalization and death from COVID-19. This protection remained strong even in studies conducted later in 2022 (44 to 80% for hospitalization and 62 to 89% for death) (Fig. 3 and figs. S6 and S7). We, however, caution that we did not examine longer periods of time since vaccination beyond 3 months.

Protection against infection was much more fragile, shown most clearly in our forest plots stratified on time since vaccination, where the decrease in VE as time progressed followed a relatively linear pattern (fig. S3). These plots also conveyed a decline in protection against infection between 1 and 3 months after booster (41 to 59% to −4 to 44%) (fig. S3).

##### Second booster

We observed that a second monovalent booster provided additional protection over the first booster against hospitalization and death (Fig. 4). However, this protection was notably lower than the protection afforded by the first booster over primary series vaccination for all outcome measures. In addition, protection against infection was very low in general (around 10 to 30% for later studies) (fig. S8).

##### Bivalent booster

Our primary literature search occurred at a time where there was little bivalent booster literature formally published in scientific journals, thus limiting its scope to monovalent boosters targeting the ancestral SARS-CoV-2 strain. However, many developed countries in late 2022 and 2023 have shifted their vaccination rollout exclusively to bivalent boosters targeting Omicron variants. While the specific VE estimates of the bivalent booster could be much different than that of the monovalent boosters that we included in our literature review, our findings on the design and analysis of VE studies are just as applicable to bivalent booster VE studies.

Our rapid review in late March 2023 offers a glimpse into the early evidence of bivalent booster effectiveness. The current results seem promising, suggesting increased effectiveness of bivalent boosters compared to monovalent boosters, at least for severe disease outcomes. Many of the more recent second monovalent booster studies reported a VE against hospitalization or severe disease of around 50% or lower, with multiple studies below 40% (Fig. 4). In contrast, out of the six bivalent booster studies with hospitalization/severe disease VE estimates, all reported a VE of at least 48%, and the second lowest VE reported was 57% (Fig. 5). Of particular interest is Lin *et al.* (*10*), who calculated VE of a monovalent and bivalent booster in the same study, finding a much higher VE against severe disease for the bivalent booster (61.8%, 95% CI: 48.2 to 71.8%) compared to the monovalent booster (24.2%, 95% CI: 1.4 to 42.8%). We note that not all the studies on the bivalent booster are encouraging: Poukka *et al.* (*34*) reported sharply waning immunity, with a severe disease VE of 26% for both a hospitalization (95% CI: −9 to 50%) and mortality (95% CI: −13 to 51%) outcome between 61 and 90 days after booster administration. The jury is still out on longer follow-up time and whether a recommended annual booster is sufficient to ensure protection in the endemic stage of the pandemic. Nevertheless, the general trend of higher effectiveness of the bivalent booster than the second monovalent booster in our review supports the rationale behind the current policy of reformulating COVID-19 vaccines annually to match current circulating variants, similar to what is done for other respiratory illnesses such as influenza.

#### Choice of designs: Cohort and/or test-negative

##### Cohort design

Cohort studies were usually viewed in a time-to-event paradigm, where the time since study start or booster administration to an outcome event of interest or censoring event was compared between fully vaccinated and boosted groups. This comparison was done using Cox regression, often adjusted for a set of covariates, or performed on a matched cohort, sometimes using stratified Cox regression with the matched sets as strata (with the final hazard ratio pooled between strata). VE was calculated as the hazard ratio from Cox regression subtracted from one, i.e.,

$$\hat{\mathrm{VE}}=1-e^{{\hat{\beta }}_{\mathrm{Vax}}}=1-{\hat{\mathrm{HR}}}_{\mathrm{Vax}:\mathrm{Outcome}}$$ (1)

We discuss additional analysis minutiae and choices for the cohort design in discussion S1.

##### Test-negative design

Statistical methods were very similar between test-negative designs for infection. Test-negative designs used logistic regression to estimate VE or conditional logistic regression if they used matching. The response variable was the test result, and explanatory variables were the vaccination status of the individual at the time of the test, along with other adjustment covariates. VE was calculated as the implied odds ratio for the vaccination status term subtracted from 1, i.e.,

$$\hat{\mathrm{VE}}=1-e^{{\hat{\beta }}_{\mathrm{Vax}}}=1-{\hat{\mathrm{OR}}}_{\mathrm{Vax}:\mathrm{Outcome}}$$ (2)

Test-negative designs were not very common for outcomes besides infection, as testing data essentially serve as a case-control for infection, but not other outcomes. Most severe disease endpoints were evaluated with a cohort design. Nonetheless, a few studies have extended the test-negative design, usually used for estimating infection VE, to evaluate VE for severe disease as a case-control. In these studies, the cases are defined as those with severe COVID-19 disease, but the control group can be defined in multiple ways, for example, those who tested negative for COVID-19, those who tested positive but did not experience severe COVID-19 disease, or severe COVID-19 disease–free individuals regardless of their test results. We give a more detailed overview of possible choices of control group in Materials and Methods.

We discuss potential issues with test-negative designs in discussion S2. We also give additional case-control designs found in the literature in discussion S3.

#### Choice of analysis: Covariate adjustment, matching, weighting, and stratification

##### Covariate adjustment

Most studies controlled potential confounding bias in VE estimates by regression adjustment (in the logistic or Cox regression models given above) of relevant covariates. We give additional details on the choice of covariates to be included in such models (or be used to match on) in discussion S4. There were several studies included in this review that presented completely unadjusted VE metrics (*18*, *35*–*37*). These were often shorter articles or preprints, often from countries or populations with little prior booster VE literature. Forgoing any type of covariate adjustment (or matching) may risk substantial confounding bias affecting VE estimates, especially for infection. These studies may add some value to the literature when they are the only VE resource, particularly in less studied populations, but need to be interpreted with substantial caution. An exception to this rule, however, may be studies that include information on all individuals nationwide, or other ecological cohorts where the study population is also the target population of inference, where general effectiveness in such a population with more complete population-level data can be ascertained without covariate adjustment (*37*).

##### Matching and weighting

Matching was a strategy used in some but not all test-negative and cohort designs. It served as an alternative way to adjust for covariates beyond the standard regression adjustment used in unmatched studies. Some matched studies used matching in lieu of regression adjustment entirely, having entirely unadjusted regressions after matching. Others matched on some covariates and adjusted for a few remaining ones. A small number of studies both matched on and regression-adjusted for numeric covariates, typically binned into several categories in the matching step, and then either treated as a continuous variable or modeled with splines within the categories in the regression step (*38*).

The most popular type of matching performed was exact matching, where only individuals with exact matches in all considered covariates were matched. This generally consisted of binning continuous variables into several bins or categories before performing exact matching. Observations were matched in either pairs or unbalanced ratios of every subject sharing the same exact matching values depending on the study. Observations not receiving an exact match were discarded.

A less common method found in the literature was propensity score matching, using the propensity for receiving a booster vaccination. We give details on the propensity score generation and matching process in discussion S5. Even less common in the literature was the use of a propensity score not via matching, but with IPW instead (*17*). This involved weighting observations in the study sample by the inverse of their propensity for vaccination/booster (more detail is included in tables S10 and S11). Compared to propensity score matching with a caliper, IPW provides potential efficiency benefits by not fully excluding any observations.

##### Stratification

Stratification was a key area where different strategies were used by different studies. Many studies did not stratify their results at all, but those that did often chose different stratification variables. One of the most popular stratification variables was time since booster vaccination. This factor was used to characterize the waning effect of the booster in the same target/source population over time. Such an analysis, however, is subject to the possibility of incurring survivorship or immortal time bias. One can try to address this issue by design or analytic choices. By forming study arms with matched pairs of boosted and nonboosted individuals, and censoring both individuals when either had an outcome or censoring event (e.g., censoring the uninfected booster individual when the non-boosted individual tests positive or vice versa), observation time can be equalized between arms even with stratification (*23*). Another method of addressing survivorship or immortal time bias that was used in the literature was target trial emulation, where observational data are analyzed in a way emulating a hypothetical (unconducted) randomized clinical trial (*39*, *40*). For the addressment of immortal time bias in any case, it is important to have accurate death data or timely national death registry integration in VE studies.

Parallel to the time since (booster) vaccination of the treatment group, a smaller number of studies also stratified on time since (primary series) vaccination of the referent group. While this review did not directly compare these estimates (i.e., with stratified forest plots), we discuss this choice further in discussion S6.

Two other stratification factors that we considered in our literature review were symptomatic infection (as opposed to any infection) and age (e.g., 65 years and older versus less than 65 years of age). Age is an important stratification condition, as individuals in an older age stratum may have a different vaccination decision process given their elevated absolute risk of severe disease. Vaccine messaging and distribution have also differed based on individuals’ age. Symptomatic infection is also an important factor to stratify on as it is a robust endpoint that remains less variable across phases and waves of the pandemic. Reporting of mild or asymptomatic infection was rare during very early days of the pandemic with limited access to testing and is increasingly rare in later stages of the pandemic. This may imply that newer infection VE studies in 2023 are practically symptomatic VE studies, reducing the need to specifically stratify on symptomatic infection (*41*). We noticed a consistently higher VE for symptomatic infection and younger age (about 10 to 15 percentage points for either).

Another important potential consideration for stratification is prior infection(s). Considering prior infection as an effect modifier may lead to heterogeneous VE estimates across strata, depending on the hybrid immunity profile. However, this was unfortunately not a focus of the booster VE literature that we found in our review, with the vast majority of studies entirely excluding individuals with prior documented infection. Only 3 of the 53 first booster studies and none of the 16 second booster studies included natural or hybrid immunity as separate strata in the analysis (*24*, *27*, *42*). This may in part be due to the evaluation of natural or hybrid immunity being undertaken in separate studies that did not meet our review’s criteria of inclusion as they did not include VE estimates (*43*). Nonetheless, studying the effect of past infections, their severity, and timing on VE estimates is an important question that will require high-quality data pooled from multiple studies in 2021–2022 when reporting behaviors were more consistent. Documented polymerase chain reaction (PCR) testing has become sporadic since the middle of 2022, and recent VE studies using real-world health care data have very little opportunity to characterize the true effect of prior infection. This is an area where a well-defined population-based cohort with serial testing, survey monitoring, serological confirmatory tests for natural infection, and regular follow-up is necessary to secure more complete information.

### MM data analysis

The VE estimates from our MM data analysis provided VEs that were relatively consistent with the literature review for the US during the relevant time period, a reassuring occurrence. Nevertheless, the main utility of the data analysis is not the specific VE estimates, but that it allowed us to apply many different statistical methods to the same health care system (with the caveat that the cohort and test-negative design have different definitions of study cohorts). While the choice of study designs can easily be classified into three categories (test-negative, cohort, and other case-control), the analytical methods are more heterogeneous. Regression adjustment and exact matching for covariates were the most commonly adopted methods to adjust for confounding in VE studies in our survey of the literature. Using a propensity score for getting boosted was also found infrequently in the literature, where the propensity score was used via matching, weighting, or adjustment. The additional class of methods we explored in our case study was the use of a propensity score for testing.

It should be noted that the methods we examined even under the same design do not all target the same estimand. First, exact and propensity score matching may alter the study sample by matching on the treated units, thus potentially changing the target population that the conclusion is generalizable to. Second, we included methods that estimated either marginal or conditional relationships. A comparison between different matching/propensity score methods is therefore not able to decompose differences in the true parameter or estimator. While we keep this fact in mind and refer to it when relevant, we note, however, that such a distinction is not crucial to the purposes of this comparison: We intended to document how different analytic decisions can change VE estimates, regardless of what side of the decomposition leads to such changes. As observed in the literature review, VE studies in practice have targeted either marginal or conditional estimands, and have certainly differed, often systematically, in target population, so such a comparison across different estimands serves only to better reflect the landscape in the literature.

For multiple methods, the infection VE using only propensity for testing was notably higher than the VE using propensity for boosting or adjustment for covariates. The VE using only propensity for testing was closer to an unadjusted estimate. This may indicate that, with an infection outcome, accounting for propensity of testing alone is not sufficient to control confounding bias. We also hoped that using two propensities (both for boosting and testing) may have helped adjusting for both confounding and selection bias. However, our results indicate that the propensity for boosting or adjusting for confounding exerts stronger influence on VE estimates, particularly for hospitalization and mortality outcomes where the effect of selection bias due to testing is limited. This observation based on one case study needs future investigation and consideration of more sophisticated methods. We give additional discussion on why this may be the case in discussion S7.

However, using a propensity score for either boosting or testing did not make a notable difference for a severe disease outcome. No statistical method had a nonoverlapping CI for severity VE. This is in part because CIs were generally wider with a limited number of events, but, particularly excluding matching methods that discard observations, point estimates were also largely in agreement. In general, our results suggest that inference regarding a severe disease outcome is more robust to changes in statistical methodology than an infection outcome, even between models targeting conditional and marginal average treatment effects.

The takeaway message from our analysis of the test-negative design suggests that, for infection VE, we may need to adjust for an expanded set of covariates despite the design attempting to control for confounding because of health care–seeking behavior. On balance, the test-negative design with weighting and covariate adjustment for the propensity for getting boosted led to narrower CIs for the severity VE compared to the cohort design. Last, extending the test-negative design to severe disease as a case-control involves a choice of several different control groups, but this only seems to lead to slight differences in results.

Our exploration in the case study using a gamut of methods on the MM data revealed that accounting for differences in the propensity for boosting plays a more critical role than accounting for differences in the propensity for testing, particularly in determining VE estimates for severity endpoints. Exact matching was a popular method in the literature. In addition, when using propensity scores, matching methods were more common than IPW. However, matching (both exact matching and with a propensity score) often leads to a loss in sample size and thus in reduced statistical efficiency. The loss of sample size is often more severe for exact matching with many covariates. IPW and assumption-lean multiple robust methods should be applied more frequently for VE studies and not just be limited to their presence in the statistical literature (*44*).

### Limitations

There are several notable limitations of our review that may prevent it from being truly representative of the entirety of booster VE literature without some level of disclaimer. First and foremost, we limited our search to English-language articles only. While our search was still able to include an international selection of articles from a broad selection of countries, this may have systematically excluded literature from certain areas of the world. In addition, we chose to limit the scope of the review to articles that estimated VE of a booster relative to the previous dose in the vaccine regimen only. Many booster VE articles, particularly for the first booster, evaluated VE as relative to no vaccination, and such articles (including 47 of 116 VE articles in our search) were excluded from our analysis (fig. S1). In addition, our forest plots, even after stratification by time since booster, grouped together in the same plot articles with major differences in design and even their target estimand (e.g., prospective versus retrospective studies, symptomatic versus all types of infection, etc.), which may affect the interpretability of these plots.

For a novel emerging disease like COVID-19, the immediate access to patient health care data like EHR and medical claims proved to be of great scientific and clinical value as there was no time to wait for evidence from large, well-designed population-based studies. Seminal papers on VE based on EHR have been published during the pandemic, especially from countries like the UK, Israel, and Denmark where there exist nationally integrated digital ecosystems, capturing patients’ encounters with the health care system in a more holistic, comprehensive way. The United States lags in terms of its quality of EHR data without having a national digital health record system. Our analysis of the MM data suffers from this fragmented data framework. For example, patients may get tested in local pharmacies and those records may not be integrated with the EHR in MM. We tried to address this issue by using primary care at MM as an exclusion or adjustment factor, but of course, this does not fully address the incomplete nature of EHR information. In addition, issues with ensuring a common clinical case definition also apply to our EHR test-negative analysis, as we discuss further in discussion S2. We acknowledge the numerous issues with EHR data including but not limited to missing data, measurement error, selection bias, and confounding. However, 7 of our 17 studies with test-negative designs used EHR data to ascertain testing status (*27*, *42*, *45*–*49*). Thus, it is important to raise these issues with imperfect EHR data and discuss potential solutions. Our case study is not used to yield another VE estimate but to compare, within the same data sources and study population, the effect of different design and analytic choices. It is reassuring that despite numerical differences, what we found in the literature review holds up in our data analysis: VE estimates for severity endpoints are robust across sensible design and analytic choices. To this end, we feel that the case study serves an important purpose.

In conclusion, as we advance into the endemic stage of the COVID-19 pandemic, infections are less likely to be reported. Our study indicates that severity endpoints are more robust to choice of statistical methodology than infection endpoints. However, since severe COVID-19 (as defined as hospitalization or death) is a rare outcome in a world with hybrid immunity (*14*), test-negative designs, which can be extended to other forms of case-control studies with severe disease outcomes, may offer more efficiency when properly used. But when using test-negative designs for infection, one must adjust for an expanded set of relevant covariates instead of purely relying on the design properties to mitigate confounding. Last, our work is not just relevant to COVID-19 vaccines but potentially may apply to other vaccination assessments as well.

## MATERIALS AND METHODS

### Literature review

#### Database search

We performed a search in the PubMed and Embase databases on 1 January 2023 for published articles or preprints written in English after 1 January 2021 that provided a VE estimate of the first booster versus primary series or second booster versus first booster against infection, hospitalization, ICU admission, or death. The search used the following four groups of terms, requiring chosen articles to include a term from each group in their title and/or abstract: (i) terms for COVID-19 generally, (ii) terms for indicating either the first or second booster, (iii) terms for a VE metric, and (iv) terms indicating real-life outcomes of interest (infection, hospitalization, ICU admission, and death). For detailed information on the search strategy, including specific verbiage for term blocks, see methods S1 and S2.

#### Article selection process

Database search results, along with several additional articles found from Google Scholar, were screened for potential duplicates by Rayyan (a web tool for literature and systematic reviews) and then removed if confirmed as a duplicate by manual title and abstract review (*50*). The remaining articles were subjected to a two-step review process of a title and abstract review followed by a full-text review. As inclusion criteria, we required included articles to (i) study a vaccine approved for COVID-19 (in any country); (ii) generate a VE metric (a proportion/percentage, odds ratio, risk ratio, or hazard ratio) against infection, hospitalization, ICU admission, and/or death; and (iii) generate the VE metric for the first booster (first dose after a primary series of vaccination, including both one- and two-dose regimens) compared to the primary series, or the second booster compared to the first booster (sometimes termed relative VE, or rVE, by papers). The first step of the title and abstract review was conducted to select articles that met the inclusion criteria in items (i) and (ii), while the second step of the full-text review selected articles that met final inclusion criteria in item (iii). We also constructed a PRISMA diagram describing this process (fig. S1) (*51*).

We additionally conducted a rapid review of bivalent boosters (which were first approved in the US in August 2022) on 28 March 2023, by searching “covid-19 ‘bivalent’ booster ‘vaccine effectiveness’” in Google Scholar. We selected articles and CDC *Morbidity and Mortality Weekly Report* releases that generated bivalent booster (targeting either the BA.1 or BA.4/5 variants) VE estimates based on the title and information shown in the preview on the search page. Unlike the main search for monovalent boosters, we did not enforce the requirement of VE being calculated compared to a specific number of previous boosters, as many studies now calculate bivalent booster VE in comparison with any number of previous monovalent boosters, but still required that VE be relative to some stage of vaccination (i.e., not relative to the fully unvaccinated).

#### Data collection and presentation

The study period, study country, general study design, dose and type of vaccine, and VE estimates (against infection, hospitalization, severe disease, and/or death for dose comparisons of interest) were collected in the full-text review of each included study. Study design was classified as one of test-negative, matched test-negative, case-control, cohort, or matched cohort. With the level of heterogeneity in vaccine brands used, study design, statistical methods, definitions of outcomes, and setting within our inclusion criteria, we did not perform any pooling of results or meta-analysis, with an emphasis on narrative synthesis instead. We visualized aggregate results by plotting VE estimates from the studies by using forest plots stratified by booster number (first or second), outcome (infection or hospitalization/severe disease or death), and time since vaccination (including a category for studies that did not stratify on time since vaccination). Studies in forest plots were displayed in (ascending) chronological order of last month in the study period, with publication date of the study used to break ties. For the primary bivalent booster forest plot, when the source studies were stratified by miscellaneous factors, we plotted results corresponding to only the oldest age, most recent variant, longest time since previous dose, and a 2-month time since boosted strata. For the stratified forest plots, we followed the above guidelines for all stratification factors except age, where different age strata (corresponding to above or below 65 or 60 years) were used to summarize strata-specific forest plots.

Forest plots were created in R, version 4.2.2 (*52*). Aggregated data files collected in the literature review and code used to create plots can be found at 10.5281/zenodo.8350757.

### MM data analysis

#### Study population

We implemented both cohort and test-negative designs with two different study populations. Study design and statistical methods for the test-negative analysis can be found in methods S3. Our cohort design study population for the main analysis consisted of 186,495 individuals after exclusion criteria (from 272,399 before exclusion criteria) who received two or three doses of the BNT162b2 (Pfizer) or mRNA-1273 (Moderna) vaccines and had at least two EHR entries at MM between 1 October 2021 and 31 December 2022. Individuals were eligible to contribute person-time from the start of the study period to their last EHR entry or the end of the study period (31 December 2022), whichever occurred first. Any individuals with a documented infection before the start of the study period or missing values of any covariates were excluded from the analysis. We present analysis results for those who were boosted before or during October to December 2021 in the main text because of more complete information with longer follow-up data. Results for all other subsequent quarters are presented in the Supplementary Materials (figs. S13, S14, S16, and S18 for the main study population).

#### Exposures

We considered three different exposure definitions (depending on booster dose) in this study in separate models. In our main analysis, we considered as exposure exactly three doses of the BNT162b2 or mRNA-1273 vaccines, and as reference two doses of BNT162b2 or mRNA-1273, both restricted to only the first 120 days of person-time, with individuals being censored after. We included both homologous and heterologous monovalent vaccine regimens, provided all doses were either BNT162b2 or mRNA-1273 (we did not include bivalent boosters). As secondary analyses, to understand the sequential benefit of serial boosting, we considered as exposure three or more doses of BNT162b2 or mRNA-1273 and four or more doses, with the reference groups being two doses and three doses, respectively.

#### Outcomes

We considered COVID-19 infection and severe COVID-19 disease as outcomes in different models. Infection was defined as a COVID-19 diagnosis or positive PCR test for SARS-CoV-2. Severe COVID-19 disease was defined as hospitalization or an ICU admission within 30 days of a positive PCR test or death within 60 days.

#### Statistical analysis

We used Cox regression to model the association between each combination of outcome and exposure in our analysis (*23*, *24*, *53*). VE was calculated by exponentiating the coefficient for vaccination, giving the hazard ratio for the outcome for those boosted compared to those in the previous stage of vaccination, and then subtracting it from one, i.e., Eq. 1 (see methods S4 for definitions of vaccination and outcome indicators). Aside from the outcome event, individuals were censored at the end of the study period, time of their last EHR entry (if before the end of the study period), death for any reason, and a positive PCR test (when infection was not the outcome event of interest).

We compared covariate adjustment, matching, and propensity score methods in the analysis. The (same) set of covariates was used for adjustment, matching, and propensity score generation: age, sex, BMI, race/ethnicity (non-Hispanic white, non-Hispanic black, other, and unknown), neighborhood development index (*54*, *55*), population density (quartiles), health care worker status, whether the subject was receiving primary care at MM or not, Charlson comorbidity index (categories of 0, 1 to 2, 3 to 4, and ≥5) (*56*), and number of documented negative SARS-CoV-2 PCR tests from the subject at any time (categories of 0, 1, 2 to 3, 4 to 5, 6 to 10, and ≥11). When used in exact matching, age was additionally categorized into three bins (18 to 49, 50 to 65, and ≥65), and BMI was categorized into four bins (underweight, healthy weight, overweight, and obese).

Matching and propensity score methods included exact matching, propensity score matching, regression adjustment for propensity score, and IPW on propensity score (IPW). We calculated separate propensity scores for both receiving a booster and reporting a SARS-CoV-2 PCR test at any time. We implemented both unstratified and stratified Cox proportional hazard models for matched data. For propensity score methods, we performed analysis with and without regression adjustment of the covariate set. Table S10 provides a listing of the methods found in our literature review or additionally proposed by us, and their relative commonality in the literature. Table S11 provides further detail on each of these methods that we apply to our MM data analysis (both cohort and test-negative), including associated model statements for the primary cohort analysis. In addition to methods in the literature that focus on propensity for getting boosted, we also included methods that include the propensity of getting tested in our data analysis, with the hope of reducing selection bias due to testing.

All analyses were conducted in R, version 4.2.2 (*52*). Code used for analysis and example code applied to a basic simulated dataset can be found at 10.5281/zenodo.8350757.

Individual-level data used in the MM VE case study cannot be shared publicly because of institutional policies around patient confidentiality and privacy concerns. The data, which were not collected by our team or as part of any other study, were curated from the electronic medical records and patient encounters at MM and made available through the University of Michigan Precision Health Analytics Platform. These data are available upon request from the University of Michigan Precision Health Analytics Platform at https://precisionhealth.umich.edu/tools-resources/data-access-tools/ for researchers who meet the criteria for access to confidential data and require Institutional Review Board (IRB) approval at their own institution and at Michigan.

Ethical review and approval for use of human subject data were waived for this study because of its qualification for a federal exemption as secondary research for which consent is not required. Determination for exemption was made by the University of Michigan Medical School IRB.

## Supplementary Materials

### This PDF file includes:

Supplementary Text Figs. S1 to S39 Legends for tables S1 to S13 References
