# Supplementary material for: Design and analysis heterogeneity in observational studies of COVID-19 booster effectiveness: A review and case study
Source: Sci Adv. 2023 Dec 20;9(51):eadj3747. doi: 10.1126/sciadv.adj3747 (PMC10732535; doi:10.1126/sciadv.adj3747)
Supplement: Supplementary file 1 — Supplementary Text Figs. S1 to S39 Legends for tables S1 to S13 References [file sciadv.adj3747_sm.pdf]

Science Advances
AAAS

Supplementary Materials for

Design and analysis heterogeneity in observational studies of COVID-19 booster effectiveness: A review and case study

Sabir Meah *et al.*

Corresponding author: Bhramar Mukherjee, bhramar@umich.edu

Sci. Adv. **9**, eadj3747 (2023)
DOI: 10.1126/sciadv.adj3747

The PDF file includes:

Supplementary Text
Figs. S1 to S39
Legends for tables S1 to S13
References

Other Supplementary Material for this manuscript includes the following:

Tables S1 to S13

Supplementary Text

Supplementary Discussion

Discussion S1: Cohort Design Additional Details

Censoring criteria for studies using Cox regression typically included documented COVID-19 infection (for non-infection endpoints), the end of the study, death for non-COVID-related reasons, or the administration of an additional dose of the COVID-19 vaccine. Individuals in the referent group for VE calculation were often allowed to re-enter the cohort as a member of the other group after receiving the next dose of the vaccine, with a period immediately following booster administration (usually 7 days, but could vary between 5-14 days depending on study) typically (but not always) discarded and not counted towards either vaccination group as the vaccine is not fully effective during this period. A small number of studies used this short time interval immediately following vaccine administration as an alternative referent group for the effectiveness of the dose in question, as this was a period where the new booster was assumed to yet have any protective effect (*59*). This provided a sensitivity analysis for selection bias of the two vaccination arms, given that it could compare the same individuals under two treatments, at the cost of making the assumption of no vaccination-conferred immunity in the immediate post-vaccination period. Cohort studies using matching would often censor both sides of the matched pair when the one in the referent group received the next dose (*60*).

Less commonly, cohort studies were analyzed in terms of counts of individuals at risk. In this situation, VE was calculated through a Poisson regression, where:

$$\widehat{VE} = 1 - e^{\hat{\beta}_{Vax}} = 1 - \widehat{IRR}_{Vax:Outcome} \qquad (3)$$

These models included calendar time as a covariate. For example, Bar-On et al. fit a model of person-days at risk for COVID-19 and adjusted for calendar day (*5*).

Discussion S2: Potential Issues with Test-Negative Designs

Test-negative designs attempt to limit confounding from healthcare seeking behaviors by limiting the sample to those presenting to testing. This is supposed to ensure that differences in outcomes between vaccination groups can be attributable to vaccination and not other differences between the vaccinated and unvaccinated. This strategy relies on the fact that similar healthcare seeking behavior is observed for both test positives and test negatives who choose to seek testing with similar symptom burden. However, in a retrospective EHR based study, this assumption may be violated when results of a PCR test are extracted from patient records for defining the tested cohort instead of using a clinical case definition. While such studies have benefited from the statistical efficiency benefits inherent to case-control studies, they may not receive the desired benefit of test-negative studies for eliminating confounding biases due to differences in healthcare seeking behavior. One may still need to adjust for healthcare access and healthcare seeking behavior via other means, such as regression adjustment, matching, or weighting using covariates such as insurance status, number of visits in the EHR and alike. Our MM analysis introduces the idea of constructing a propensity for testing that might also aid with reduction of such confounding biases.

Discussion S3: Additional Case-Control Designs

As an alternative strategy, a few test-negative studies used a secondary matched non-test-negative case-control design to estimate VE against severe disease endpoints (*45*). A few studies that evaluated VE against hospitalization but not infection tackled this control-group problem by designing a case-control sample among those hospitalized, not those presenting for infection, with matching used to ensure the appropriateness of controls (*29*). Another case-control design used as cases those dead from COVID-19, and controls were risk-set sampled and matched from the healthy at-risk population at the time of the case's death (*30*).

Discussion S4: Choice of Adjustment or Matching Covariates

Regression adjustment covariates (or matching covariates in studies using matching and not adjusting further on these covariates) differed somewhat between studies, but generally followed similar patterns. Most studies adjusted or matched on age and sex, and many also on one or more covariates related to the subject's economic status (including race/ethnicity and living conditions), along with comorbidities, as either a comorbidity index or a set of several covariates each responding to individual comorbidities. Studies with longer follow-up periods had adjustment for time periods at risk. Test-negative studies often adjusted for the time of the test. Some studies adjusted for exposure to disease in healthcare settings. Without the convenient built-in control that a test-negative design offered, many cohort studies also adjusted for the number of negative PCR tests taken either before the study or in total before the study end date, accounting in some sense for inclination for testing (*22*).

Discussion S5: Propensity Score Generation and Matching

Propensity score matching consisted first of the generation of a propensity for receiving a booster through the regression of the covariates on the vaccination status variable. Pairs of subjects with different exposure statuses were then matched with a nearest neighbor algorithm (see Table S11). Some studies introduced a caliper, or maximum allowable distance between propensity scores for matching observations, with matches with a greater distance discarded. Previous literature (*61*) has suggested a caliper of 0.2 standard deviations of the logit of the propensity score as a recommended value, and this was the value typically used by studies using propensity score matching (*39*).

Discussion S6: Time Since Primary Series Vaccination Stratification

A parallel consideration to stratification on time since booster for the treatment group was stratification or inclusion criteria on time since primary series vaccination for the referent group; shorter amounts of time provide a comparison of two fresh vaccine regimens, while a longer period of time provides a more useful policy decision on the utility of vaccination for those booster-eligible (in some cases both were provided (*37*)). Another popular stratification condition, at least in countries with different widely used vaccinations, was brand of booster, primary series, or entire vaccination series, with mRNA boosters sometimes (but often not) being combined in one stratum. A type of stratification used in studies with longer follow-up time was stratification on type of variant (usually done on a date of variant dominance, as opposed to genetic testing of cases), done most commonly in studies that spanned both Delta and Omicron periods (*62*).

Discussion S7: Additional Discussion on Propensity for Testing vs. Propensity for Boosting

Adjusting for the propensity of testing accounts for the difference between the study sample (the tested individuals) and the broader population (both the tested and untested individuals), i.e., selection bias inherent in test-negative designs. Adjusting for the propensity of vaccination, on the other hand, accounts for the difference between the boosted group and the control group (within the study sample of all tested individuals), i.e., confounding bias in an observational study.

We do not expect adjustment for the propensity of testing to control for confounding bias. Thus, the observation that for the PSA and IPW method, the VE using only propensity for testing was closer to an unadjusted estimate is reasonable. In addition, the observation that for the PSA-CA and IPW-CA method, the VE estimates were much more similar regardless of which propensity score is used, indicates that covariate adjustment accounted for confounding bias, and selection bias is not substantial in the MM data (because results with and without adjustment for selection bias were similar).

Without doing a proper simulation study with varying degrees of confounding and testing coverage, it is impossible to quantify or generalize the properties of the estimators that adjust for both propensities as opposed to the estimators that adjust for one.

Supplementary Methods

Methods S1: Search terms for PubMed Search
Date searched: 1/1/2023
Number of results: 217
Date filter: January 1, 2021 to present
Language filter: English

Search Blocks

1. "covid-19"[Text Word] OR "COVID19"[Text Word] OR "COVID-19"[Supplementary Concept] OR "SARS-CoV-2"[Text Word] OR "SARS-CoV2"[Text Word] OR "severe acute respiratory syndrome coronavirus 2"[Supplementary Concept] OR "severe acute respiratory syndrome coronavirus 2"[Text Word] OR "2019-nCoV"[Text Word] OR "2019nCoV"[Text Word] OR "coronavirus"[Text Word] OR "coronavirus"[MeSH Terms]
2. "booster"[Title] OR "boosters"[Title] OR "third dose"[Title] OR "fourth dose"[Title] OR "3 doses"[Title] OR "4 doses"[Title]
3. "effectiveness"[Title/Abstract] OR "effective"[Title/Abstract] OR “effect”[Title] OR “effects”[Title] OR "odds"[Title] OR "protection"[Title] OR “association”[Title] OR “comparison”[Title] OR “comparing”[Title]
4. "infection"[Title/Abstract] OR "hospitalization"[Title/Abstract] OR "COVID-19 outcomes"[Title/Abstract] OR "COVID19 outcomes"[Title/Abstract] OR "covid 19 outcomes"[Title/Abstract] OR "severe COVID-19"[Title/Abstract] OR "severe COVID19"[Title/Abstract] OR "severe covid 19"[Title/Abstract] OR "confirmed COVID-19"[Title/Abstract] OR "confirmed COVID19"[Title/Abstract] OR "confirmed covid 19"[Title/Abstract] OR “ICU”[Title/Abstract] OR “intensive care”[Title/Abstract] OR “mortality”[Title/Abstract] OR “death”[Title/Abstract]

Methods S2: Search terms for Embase Search
Date Searched: 1/1/2023
Number of Results: 96
Date filter: January 1, 2021 to present
Language filter: English
Other filters applied: exclude MedLine (only Embase and preprints), only articles and preprints (exclude conference abstracts and reviews)

Search Blocks:

1. 'covid 19':ti,ab OR covid19:ti,ab OR 'sars cov 2':ti,ab OR 'sars cov2':ti,ab OR 'severe acute respiratory syndrome coronavirus 2':ti,ab OR '2019 ncov':ti,ab OR 2019ncov:ti,ab OR coronavirus:ti,ab OR 'coronavirinae'/exp OR 'coronavirus disease 2019'/exp OR covid:ti,ab
2. 'booster':ti OR 'boosters':ti OR 'third dose':ti OR 'fourth dose':ti OR '3 doses':ti OR '4 doses':ti
3. 'effectiveness':ti,ab OR 'effective':ti,ab OR 'effect':ti OR 'effects':ti OR 'odds':ti OR 'protection':ti OR 'association':ti OR 'comparison':ti OR 'comparing':ti
4. "infection":ti,ab OR "hospitalization":ti,ab OR "COVID-19 outcomes":ti,ab OR "COVID19 outcomes":ti,ab OR "covid 19 outcomes":ti,ab OR "severe COVID-19":ti,ab OR "severe COVID19":ti,ab OR "severe covid 19":ti,ab OR "confirmed COVID-19":ti,ab OR "confirmed COVID19":ti,ab OR "confirmed covid 19":ti,ab OR "ICU":ti,ab OR "intensive care":ti,ab OR "mortality":ti,ab OR "death":ti,ab

Methods S3: Test-negative methods

Study Population

Our study population for the main analysis consisted of 65,992 individuals (75,645 individuals for the two or more booster secondary analysis) after exclusion criteria (from 115,660 individuals before exclusion criteria) who received two or three doses of the BNT162b2 (Pfizer) or mRNA-1273 (Moderna) vaccines and were tested for (including both positive and negative results) or diagnosed with COVID-19 at MM between October 1, 2021 and December 31, 2022. We allowed each individual to contribute up to one test per quarter, treating tests as the unit of observation in models. Any tests taken after a previous positive test or documented infection were excluded. We also restricted the data to complete cases of all the covariates used in analysis, which are given below.

Exposures

We used the same exposures as in the cohort analysis.

Outcomes

We considered the same outcomes as in the cohort analysis.

Statistical Analysis

For each combination of study population and outcome, we fit several models using a variety of different adjustment, matching, or propensity score strategies to the data. As a secondary analysis, we, also for each combination of study population and outcome, fit unadjusted and unadjusted models (not including matching or propensity scores) using different quarters of study start dates (only including tests that occurred during or after the study start quarter) between Q4 2021 and Q4 2022.

Models in the test-negative analysis used logistic regression, formulated as:

$$\text{logit}[P(Y_{outcome} = 1 | I(Vaccine), Covariates)] = \beta_0 + \beta_{Vax} I(Vaccine) + \beta_{Cov} Covariates \quad (4)$$

(see Methods S4 for definitions of vaccination and outcome indicators). Some models utilizing matching used conditional logistic regression, an extension of logistic regression with matching stratum-specific intercepts, formulated as:

$$\text{logit}[P(Y_{outcome} = 1 | I(Vaccine), Covariates)] = \beta_{0,j|i} + \beta_{Vax} I(Vaccine) + \beta_{Cov} Covariates \quad (5)$$

VE was calculated by exponentiating the coefficient for vaccination, giving the odds ratio for the outcome for those boosted compared to those in the previous stage of vaccination, then subtracting it from one, i.e.,

$$\widehat{VE} = 1 - e^{\hat{\beta}_{Vax}} = 1 - \widehat{OR}_{Vax:Outcome} \quad (6)$$

For infection models, cases were considered as individuals who tested positive or were diagnosed with COVID-19 and controls those who tested negative.

Test-negative designs can be extended as case-controls to consider severe disease as an endpoint. These designs are variants of case-control designs where cases are defined as those who experience severe disease, but the control group may be defined with test results being part of the control ascertainment process.

For example, we can consider a 2x2 table of severe disease status versus test result status that may lead to the control group definitions in the subsequent table:

Table S12. 2x2 Table of severe disease versus test positivity.

	**Severe Disease**	**No Severe Disease**	
**Test-Positive**	Case	Potential Control A	
**Test-Negative**	Potential Control B	Potential Control C	

Table S13. Choices of control group for test-negative extension to severe disease. Choices of control groups for the extension of a test-negative study to severe disease outcomes as a case-control, expressed in terms of the entries of the 2x2 Table S12.

Control Group	2x2 Table Potential Controls

1 - Test-negative	B, C
2 - Non-hospitalized	A, C
3 - Test-positive non-hospitalized	A
4 - Test-negative non-hospitalized	C

We considered control groups 1, 2, and 3 shown above in the MM analysis. In sum, cases were considered those with severe disease defined above, and we fit models with three different choices of controls: those who tested negative (control group 1), those who had no severe disease regardless of test results (control group 2), and those who tested positive but had no severe disease (control group 3). For the last model, final VE was calculated as:

$$\widehat{VE} = 1 - \widehat{OR}_{Vax:Infection} * \widehat{OR}_{Vax:Severe\ Disease} \quad (7)$$

where the odds ratio for infection is obtained from a model using the same design otherwise with infection and not severe disease as the outcome variable in logistic regression.

We adjusted for the same set of covariates as done in the cohort models, except with the addition of quarter of test.

Unadjusted logistic regression and adjusted logistic regression for the covariates were fit to the data. In addition, we fit models using the same matching and propensity score methods used with the cohort data (exact matching, propensity score caliper matching, models adjusted for propensity for boosting, and models with IPW for propensity for boosting) and explained further in Table S11 (except note that unconditional logistic regression was used in lieu of unstratified Cox regression, and conditional logistic regression was used in lieu of stratified Cox regression).

Methods S4: Definitions of terms in models (vaccination and outcome indicators)

Exposures:

For first booster models:

$$I(Vaccine) = \begin{cases} 0, & \text{fully vaccinated} \\ 1, & \text{boosted} \end{cases} \quad (8)$$

For second booster models:

$$I(Vaccine) = \begin{cases} 0, & \text{once boosted} \\ 1, & \text{boosted twice or more} \end{cases} \quad (9)$$

Outcomes:

For infection models:

$$I(Outcome) = \begin{cases} 0, & \text{tested negative} \\ 1, & \text{tested positive} \end{cases} \quad (10)$$

For severe disease models:

$$I(Outcome) = \begin{cases} 0, & \text{no severe disease} \\ 1, & \text{severe disease} \end{cases} \quad (11)$$

Supplementary Figures

Literature Review

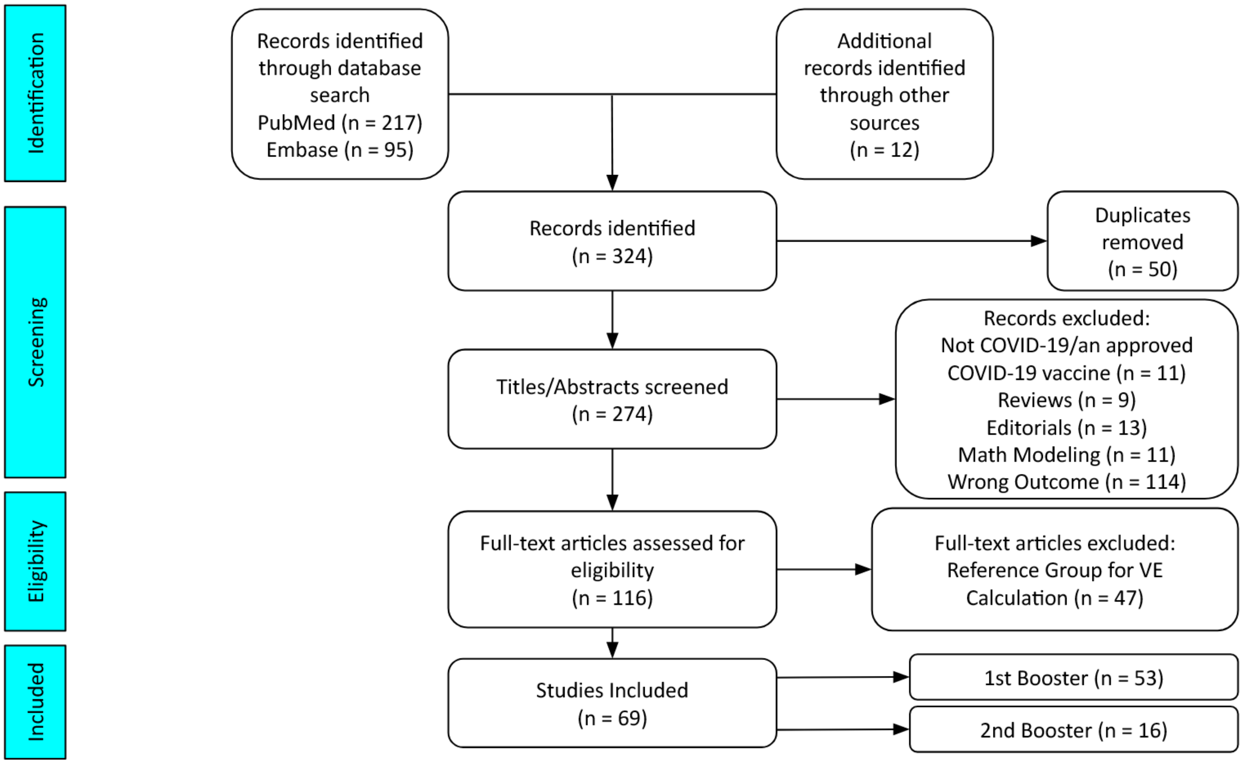

Fig. S1. **PRISMA flow diagram for the selection process of studies in the literature review**.

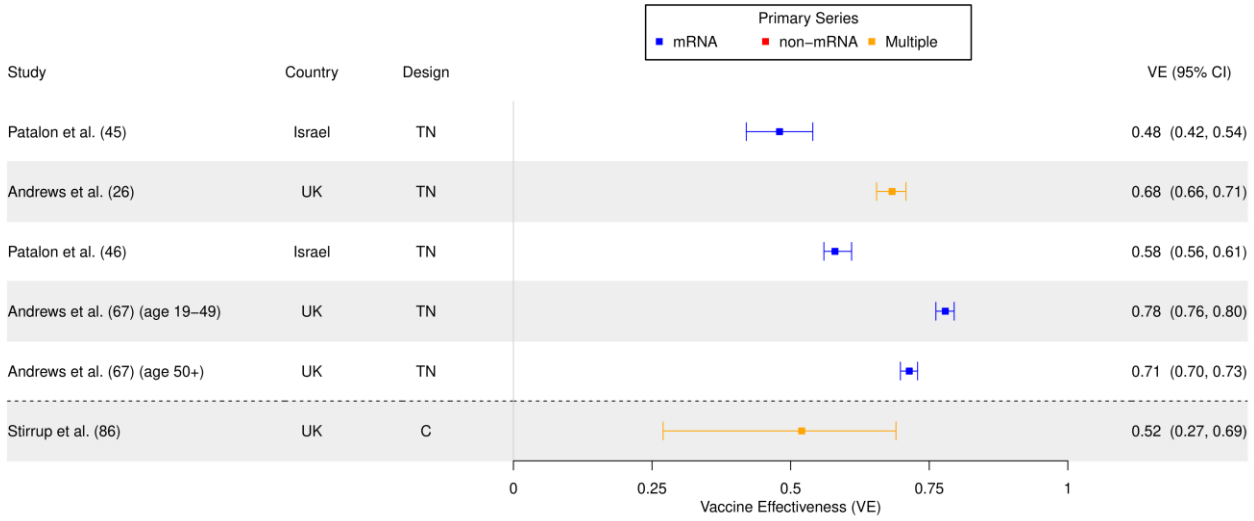

Fig. S2. **First booster VE compared to primary series against infection between 1- and 2-weeks post-booster vaccination**. Studies are sorted in chronological order of last month included in the study period (with publication date being used to break ties). The dotted line indicates a division between studies ending in 2021 and ending in 2022 (noting that Omicron became the dominant variant in many countries around the end of 2021).

Abbreviations: TN, test-negative; MTN, matched test-negative; CC, case-control (non-test-negative); MCC, matched case-control (non-test-negative); C, cohort; MC, matched cohort.

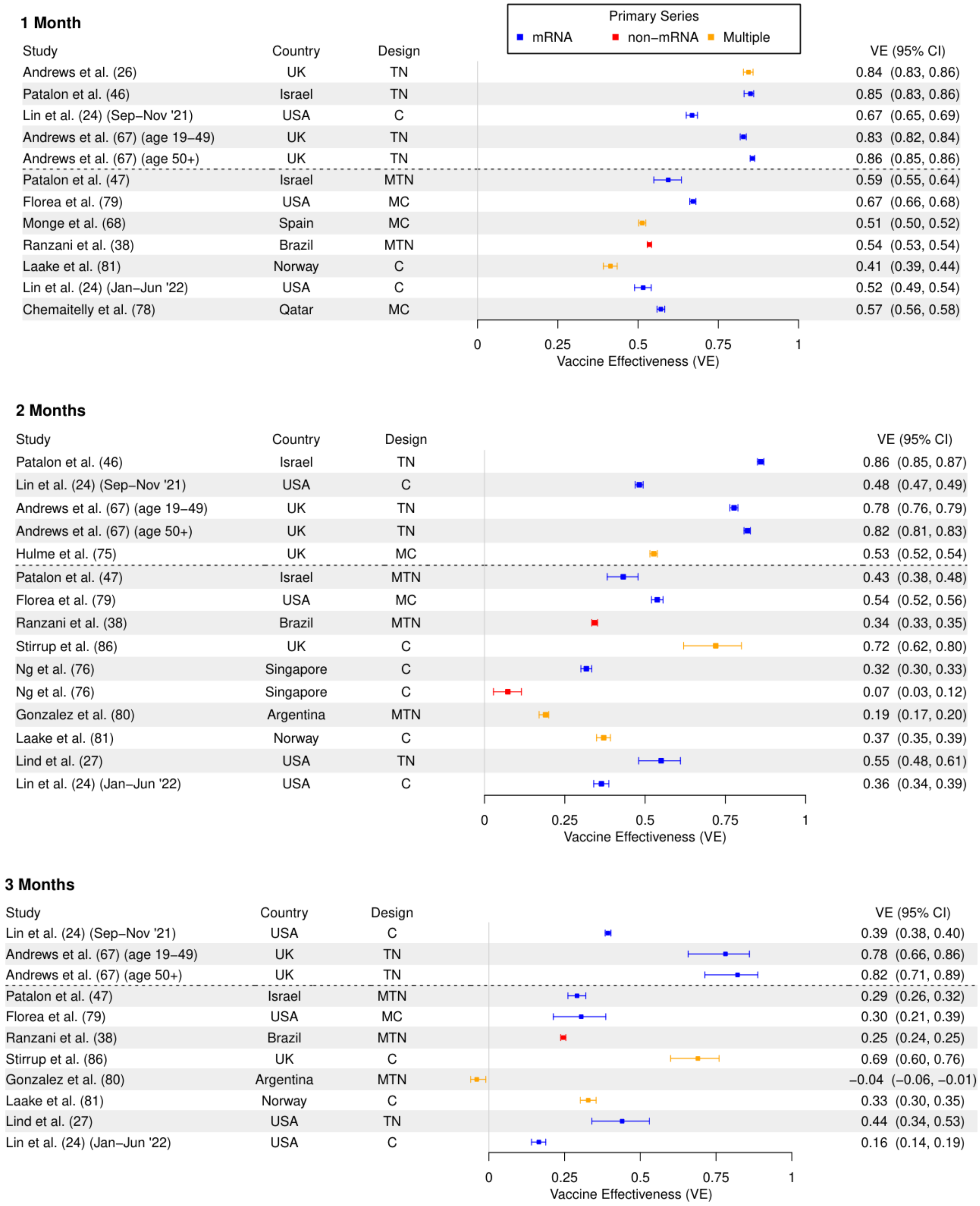

Fig. S3. First booster VE compared to primary series against infection at 1, 2, and 3 months after booster vaccination. Studies are sorted in chronological order of last month included in the study period (with publication date being used to break ties). The dotted line indicates a

division between studies ending in 2021 and ending in 2022 (noting that Omicron became the dominant variant in many countries around the end of 2021).
Abbreviations: TN, test-negative; MTN, matched test-negative; CC, case-control (non-test-negative); MCC, matched case-control (non-test-negative); C, cohort; MC, matched cohort.

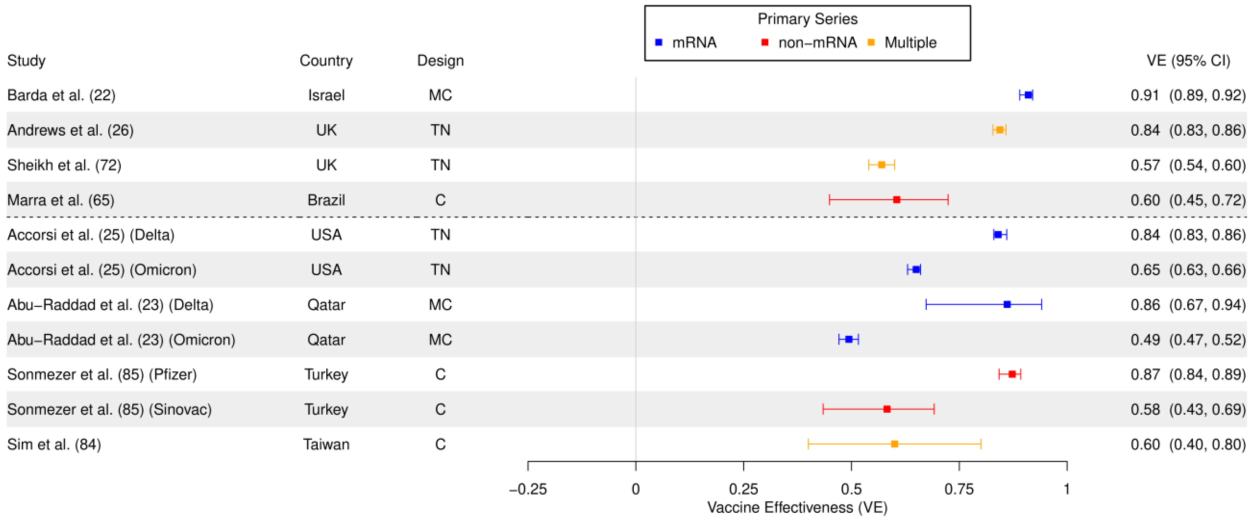

Fig. S4. First booster VE compared to primary series against symptomatic infection (in studies unstratified by time since booster). Studies are sorted in chronological order of last month included in the study period (with publication date being used to break ties). The dotted line indicates a division between studies ending in 2021 and ending in 2022 (noting that Omicron became the dominant variant in many countries around the end of 2021).
Abbreviations: TN, test-negative; MTN, matched test-negative; CC, case-control (non-test-negative); MCC, matched case-control (non-test-negative); C, cohort; MC, matched cohort.

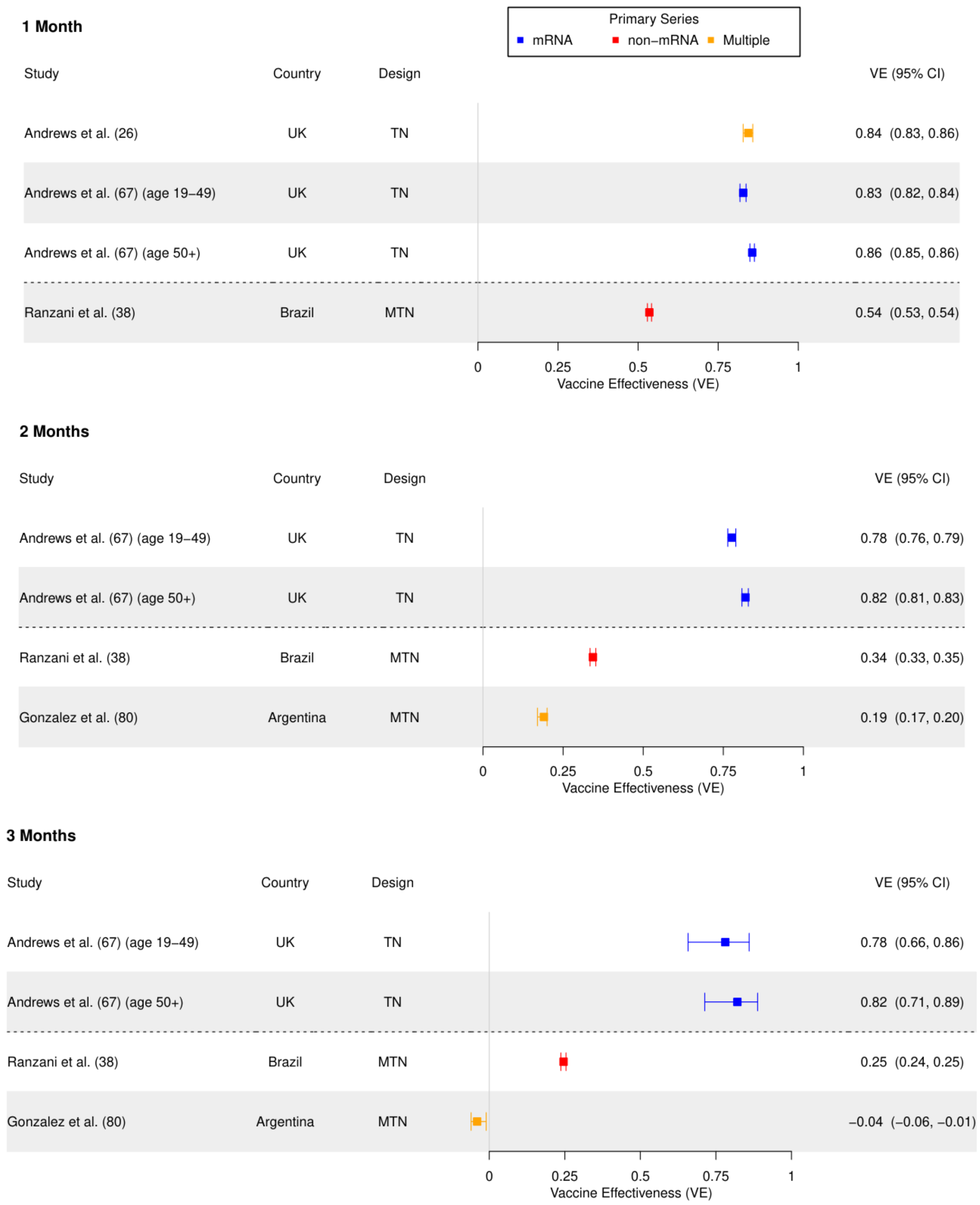

Fig. S5. First booster VE compared to primary series against symptomatic infection at 1, 2, and 3 months after booster vaccination. Studies are sorted in chronological order of last month included in the study period (with publication date being used to break ties). The dotted line

indicates a division between studies ending in 2021 and ending in 2022 (noting that Omicron became the dominant variant in many countries around the end of 2021).
Abbreviations: TN, test-negative; MTN, matched test-negative; CC, case-control (non-test-negative); MCC, matched case-control (non-test-negative); C, cohort; MC, matched cohort.

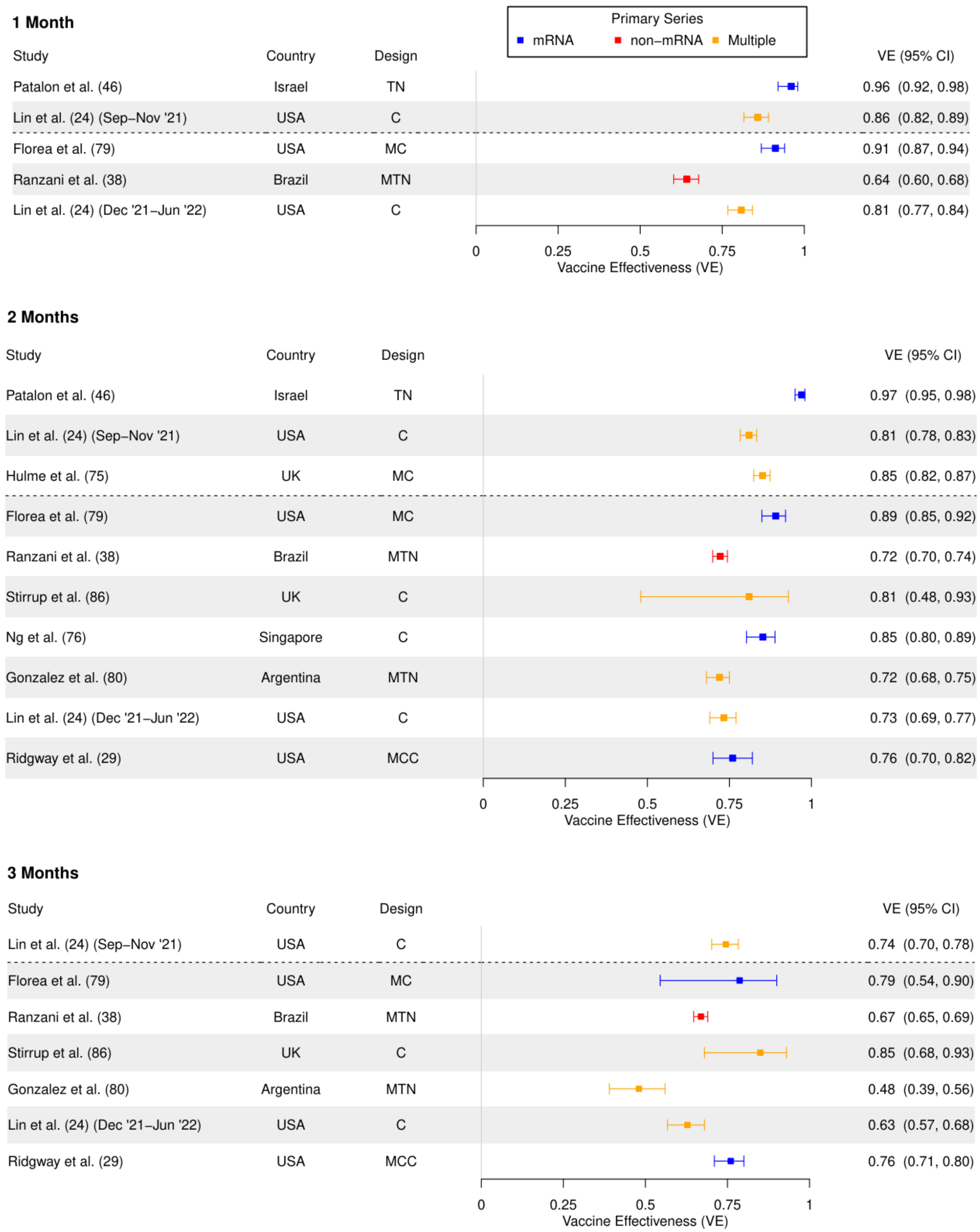

Fig. S6. First booster VE compared to primary series against hospitalization or severe disease outcomes at 1, 2, and 3 months after booster vaccination. Studies are sorted in chronological order of last month included in the study period (with publication date being used to break

ties). The dotted line indicates a division between studies ending in 2021 and ending in 2022 (noting that Omicron became the dominant variant in many countries around the end of 2021). Abbreviations: TN, test-negative; MTN, matched test-negative; CC, case-control (non-test-negative); MCC, matched case-control (non-test-negative); C, cohort; MC, matched cohort.

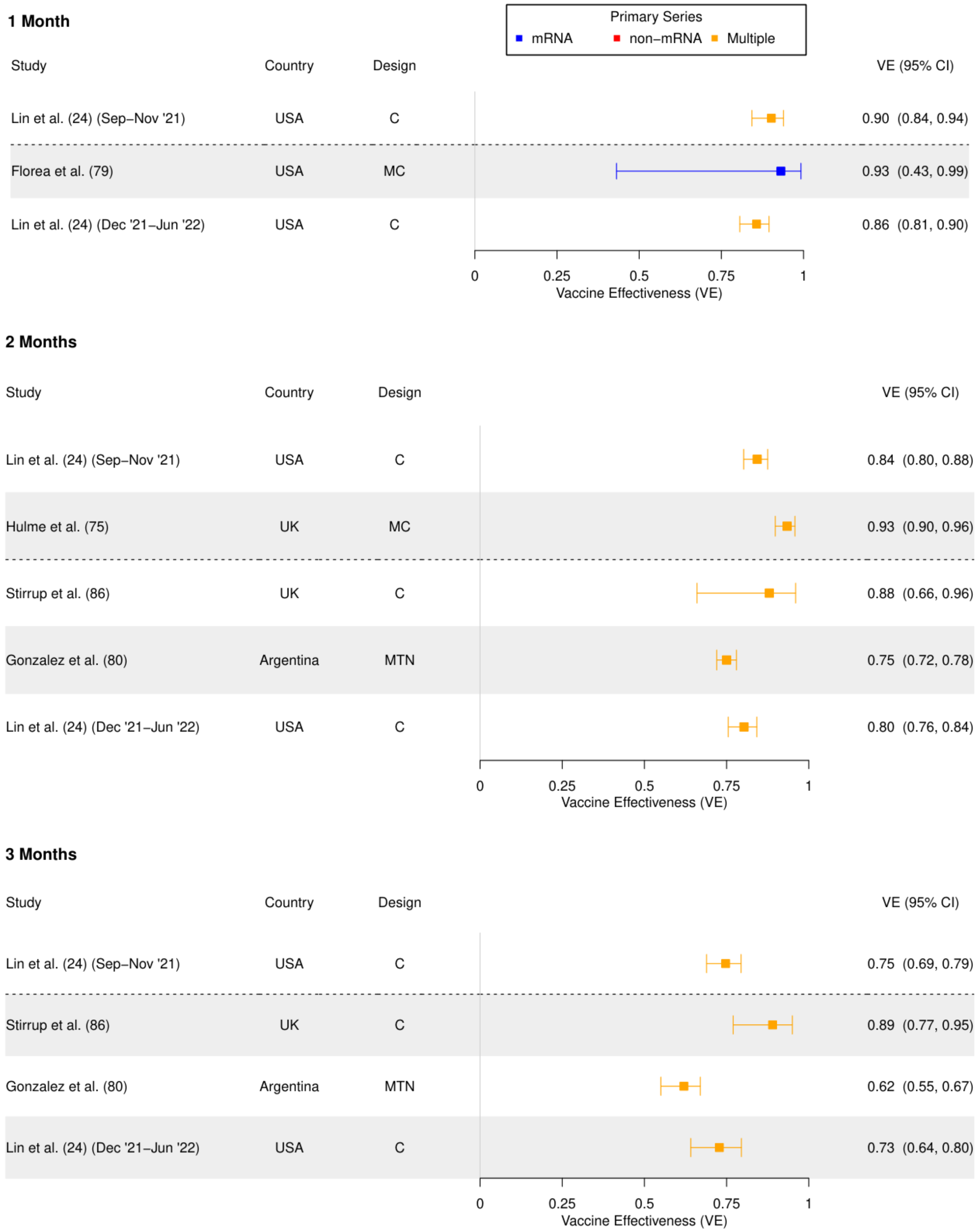

Fig. S7. **First booster VE compared to primary series against mortality at 1, 2, and 3 months after booster vaccination**. Studies are sorted in chronological order of last month included in the study period (with publication date being used to break ties). The dotted line indicates a

division between studies ending in 2021 and ending in 2022 (noting that Omicron became the dominant variant in many countries around the end of 2021).

Abbreviations: TN, test-negative; MTN, matched test-negative; CC, case-control (non-test-negative); MCC, matched case-control (non-test-negative); C, cohort; MC, matched cohort.

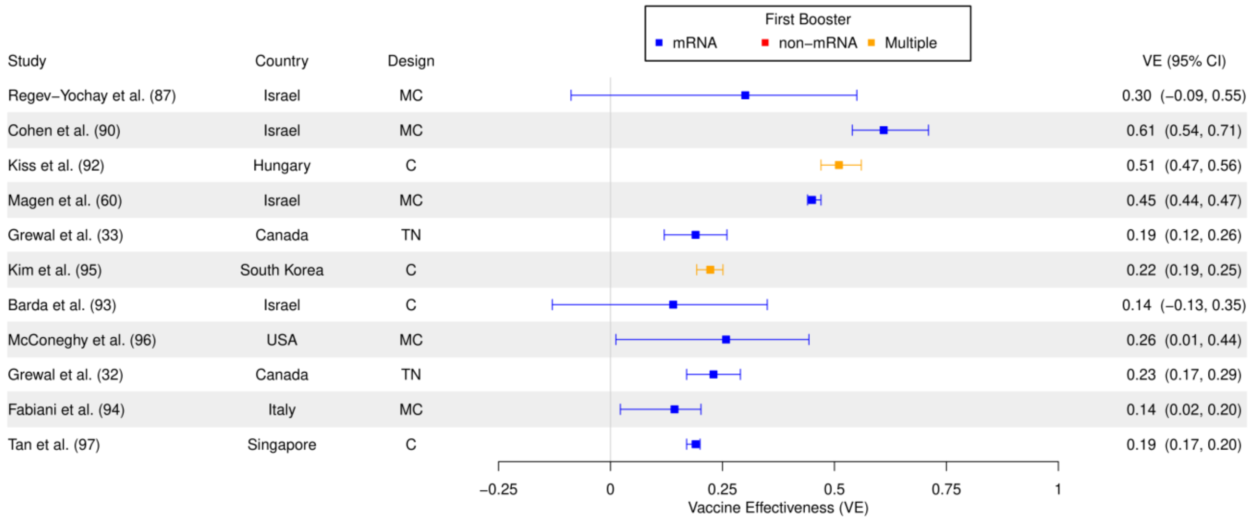

Fig. S8. Second booster VE compared to first booster against infection unstratified by time since booster vaccination. Studies are sorted in chronological order of last month included in the study period (with publication date being used to break ties). Both symptomatic and overall results are included for studies that produced estimates for both, indicated by "(sympt)" and "(all)", respectively.

Abbreviations: TN, test-negative; MTN, matched test-negative; CC, case-control (non-test-negative); MCC, matched case-control (non-test-negative); C, cohort; MC, matched cohort.

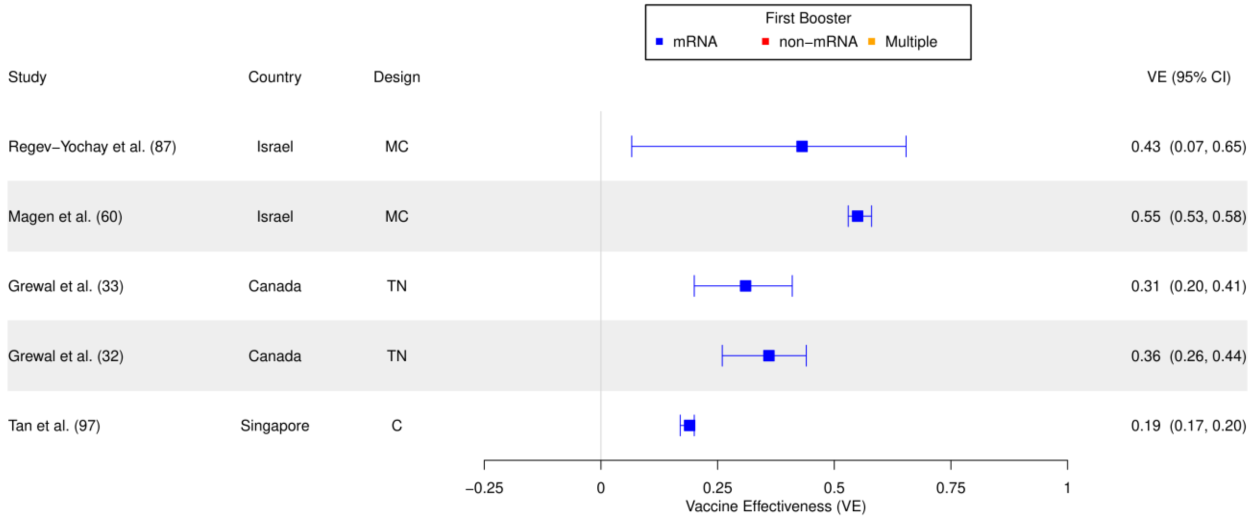

Fig. S9. **Second booster VE compared to first booster against symptomatic infection unstratified by time since booster vaccination**. Studies are sorted in chronological order of last month included in the study period (with publication date being used to break ties). Abbreviations: TN, test-negative; MTN, matched test-negative; CC, case-control (non-test-negative); MCC, matched case-control (non-test-negative); C, cohort; MC, matched cohort.

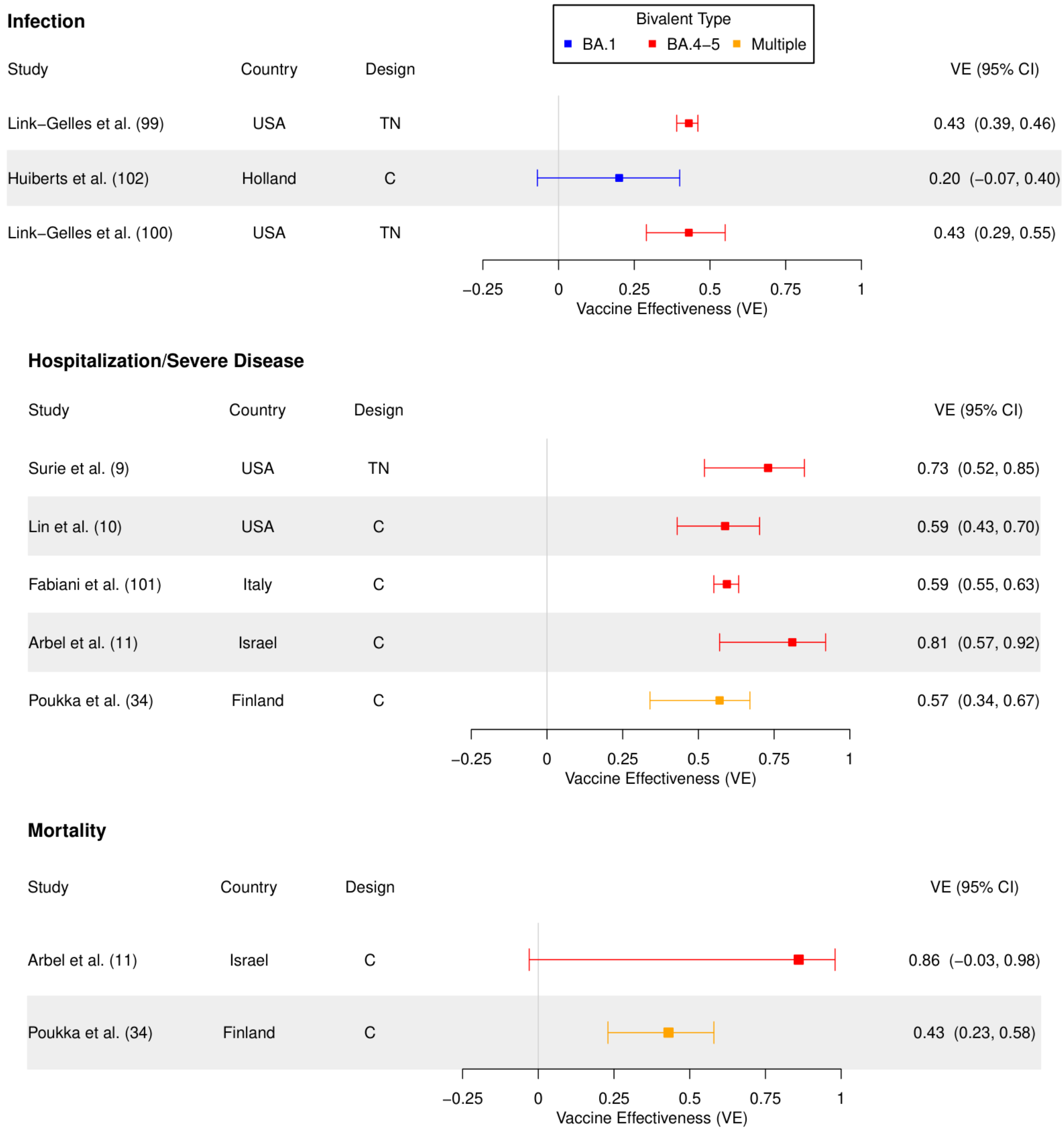

Fig. S10. Bivalent booster (targeting either BA.1 or BA.4/5) VE compared to a previous dose against infection, hospitalization/severe disease, and mortality only in separate panels among those aged 65 or older (or 60 or older). Studies are sorted in chronological order of last month included in the study period (with publication date being used to break ties). For studies with stratified results, we displayed results corresponding to only the oldest age, most recent variant, longest time since previous dose, and a 2-month time since boosted strata. Abbreviations: TN, test-negative; MTN, matched test-negative; CC, case-control (non-test-negative); MCC, matched case-control (non-test-negative); C, cohort; MC, matched cohort.

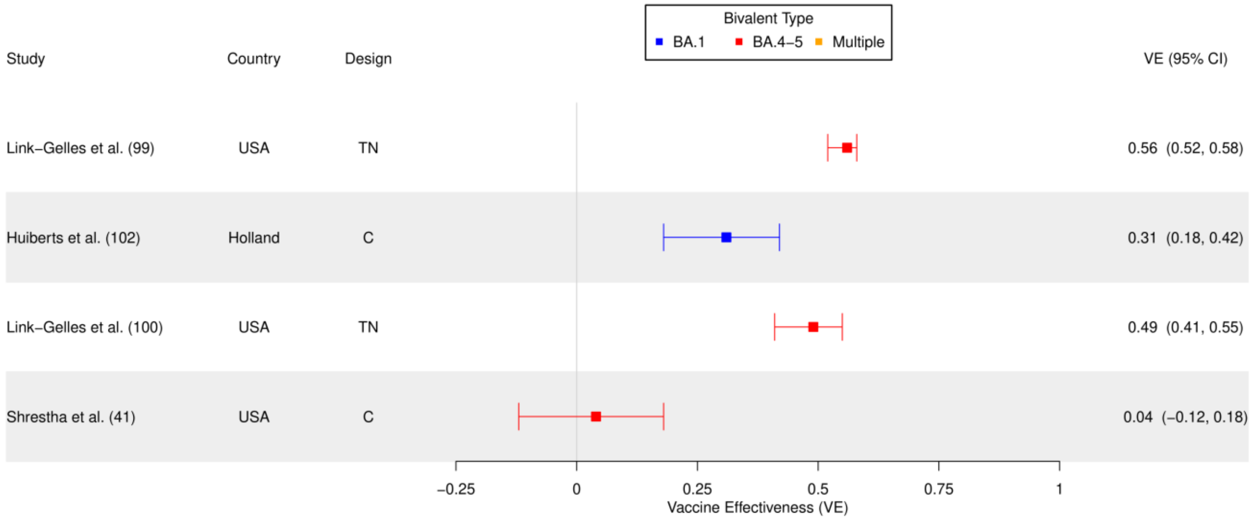

Fig. S11. Bivalent booster (targeting either BA.1 or BA.4/5) VE compared to a previous dose against infection only in separate panels among those aged 18-65 (or 18-60). Studies are sorted in chronological order of last month included in the study period (with publication date being used to break ties). For studies with stratified results, we displayed results corresponding to only the oldest age, most recent variant, longest time since previous dose, and a 2-month time since boosted strata.

Abbreviations: TN, test-negative; MTN, matched test-negative; CC, case-control (non-test-negative); MCC, matched case-control (non-test-negative); C, cohort; MC, matched cohort.

First Booster vs. Primary Series

Cohort Infection and Severe Disease – First Booster vs. Primary Series

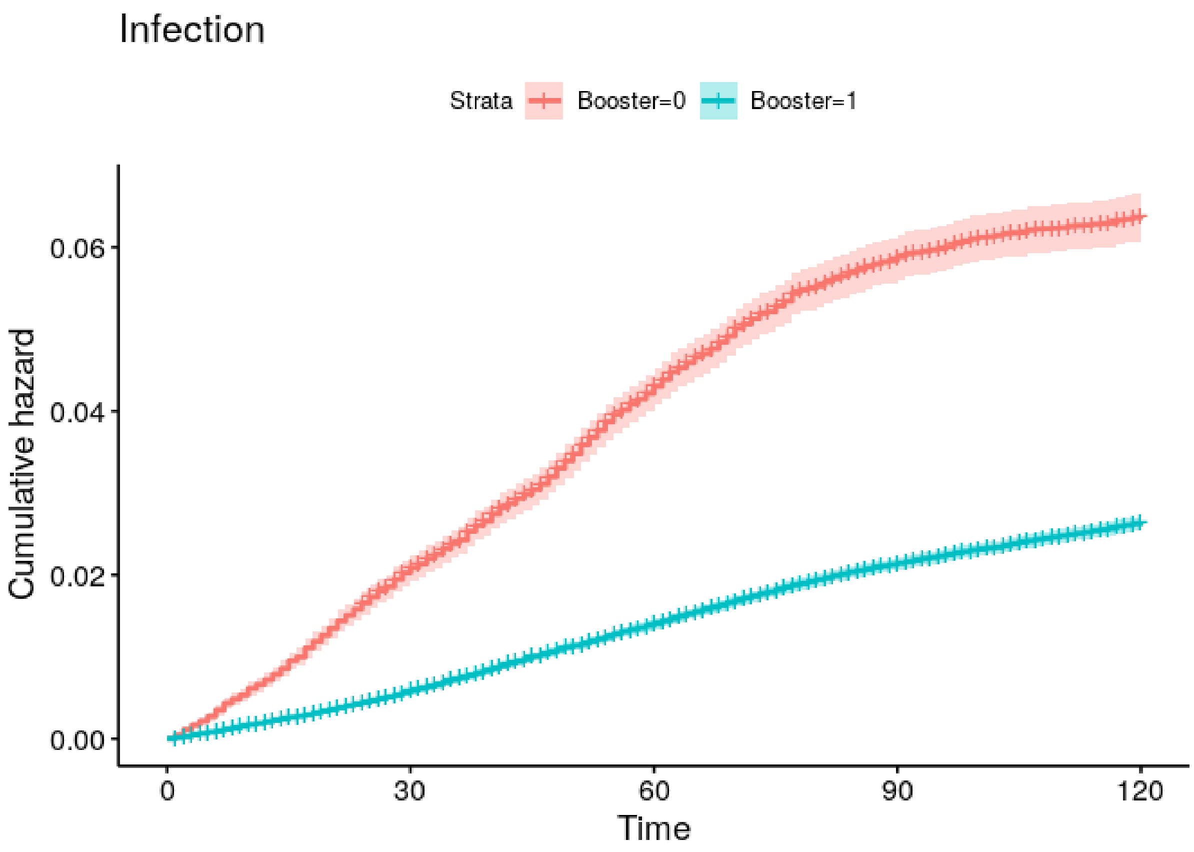
Infection
Strata
Booster=0
Booster=1
Cumulative hazard
0.06
0.04
0.02
0.00
0
30
60
90
120
Time

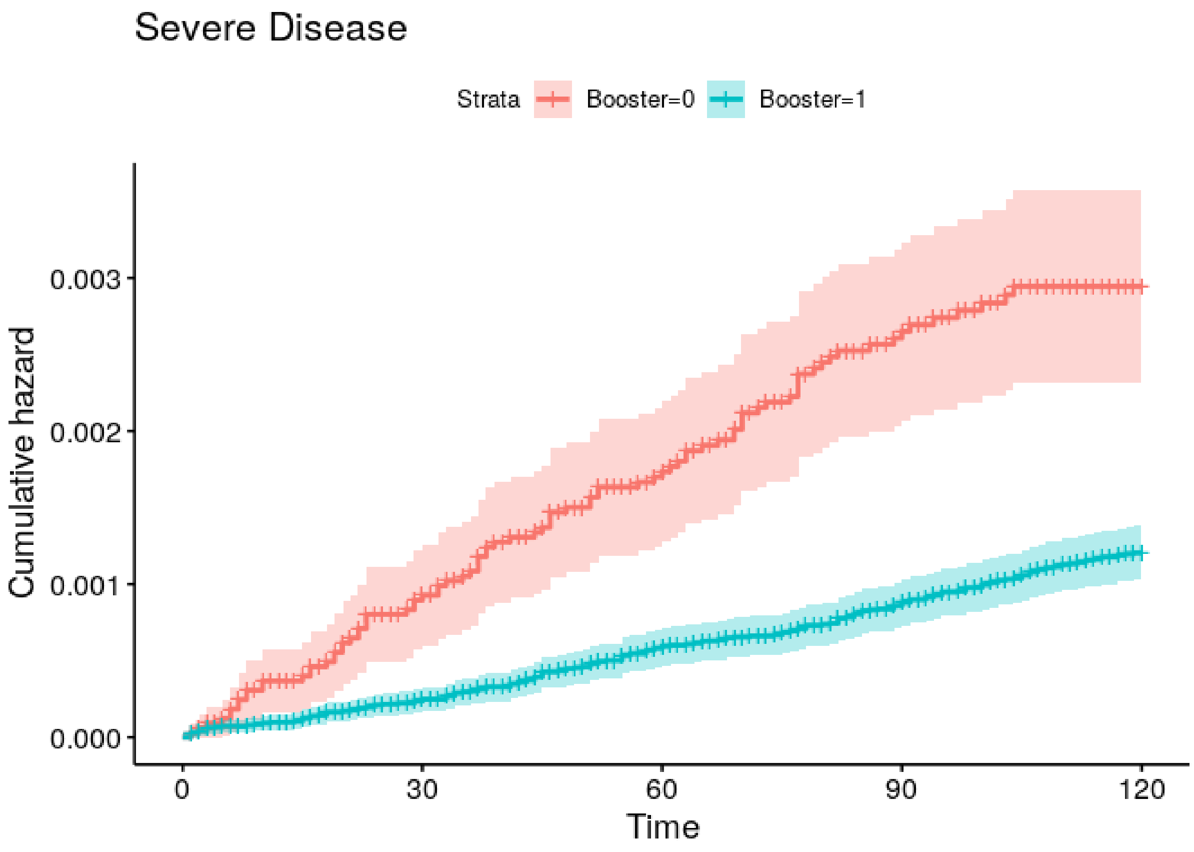
Severe Disease
Strata
Booster=0
Booster=1
Cumulative hazard
0.003
0.002
0.001
0.000
0
30
60
90
120
Time

Fig. S12. Kaplan-Meier curves for first booster/third dose vs full vaccination/second dose VE against infection and severe disease. These curves describe a study period of October 1, 2021 to December 31, 2022

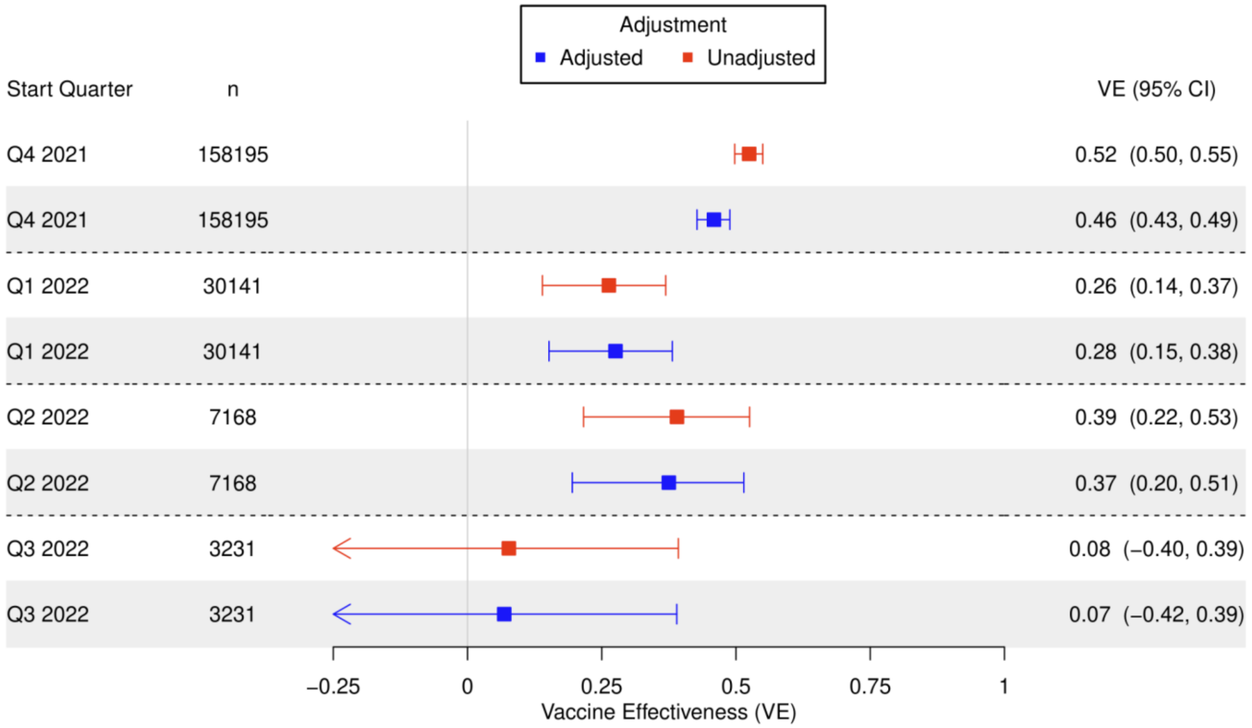

Fig. S13. Cohort first booster/third dose vs full vaccination/second dose VE against infection with various study start (index) dates. All study periods use an end date of Dec 31, 2022.

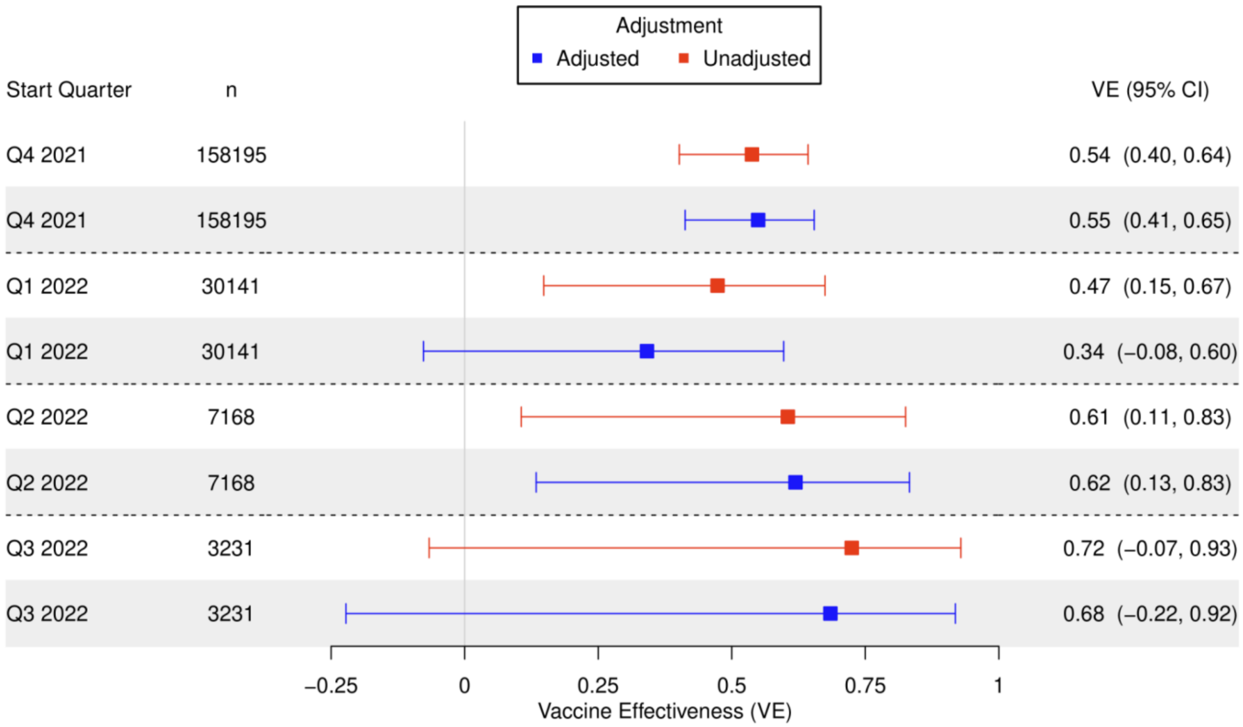

Fig. S14. **Cohort first booster/third dose vs full vaccination/second dose VE against severe disease, with various study start (index) dates**. All study periods use an end date of Dec 31, 2022.

Test-Negative Infection – First Booster vs. Primary Series

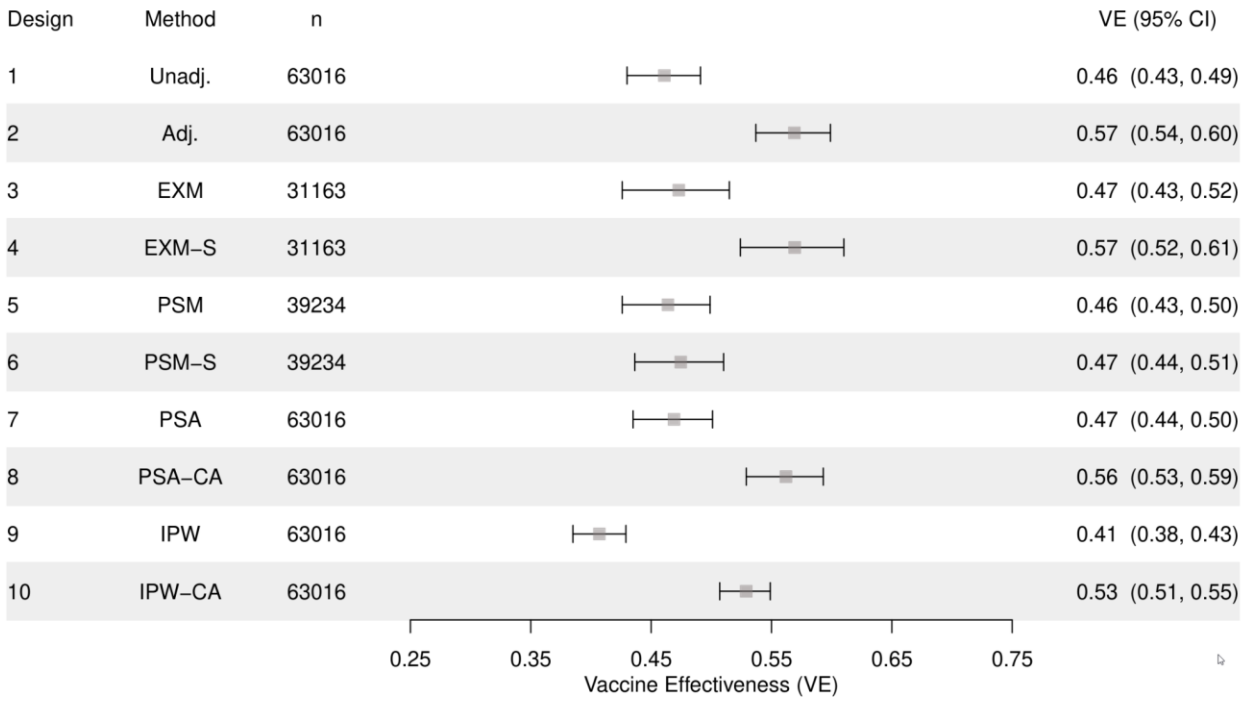

Fig. S15. **Test-negative first booster/third dose up to 2 quarters since vaccination vs full vaccination/second dose VE against infection**. We apply various matching designs during a study period of October 1, 2021 to December 31, 2022.
Abbreviations: Unadj., unadjusted; Covar. Adj., adjusted for covariates; Exact, exact matching; Exact Strat., exact matching accounting for matching strata in conditional logistic regression; Prop. Cal., propensity score matching with a 0.2 caliper; Prop. Cal. Strat, propensity score matching with a 0.2 caliper accounting for matching strata in conditional logistic regression; Prop. Adj.; adjustment for propensity score; Prop. + Covar. Adj., adjustment for propensity score and for covariates; IPW, inverse probability weighting of propensity score; IPW Covar. Adj., inverse probability weighting of propensity score also adjusting for covariates.

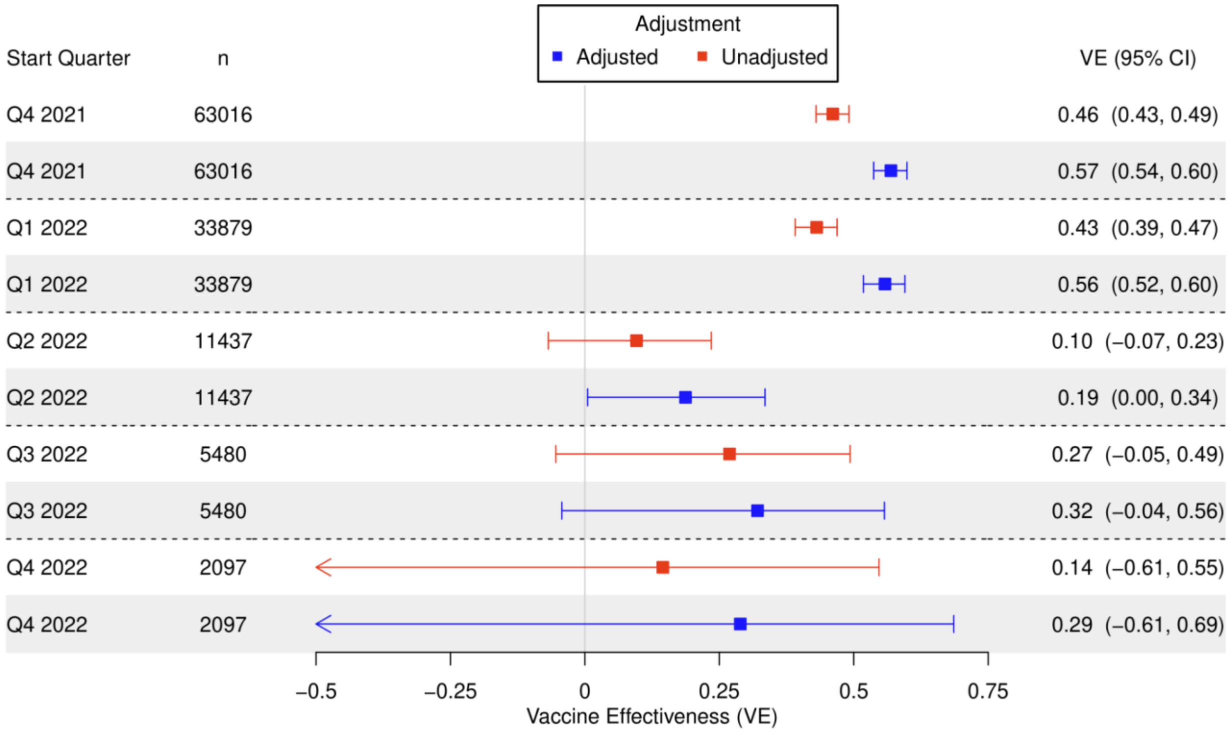

Fig. S16. **Test-negative first booster/third dose up to 2 quarters since vaccination vs full vaccination/second dose VE against infection, with various study start (index) dates**. All study periods use an end date of Dec 31, 2022.

Test-Negative Severe Disease – First Booster vs. Primary Series

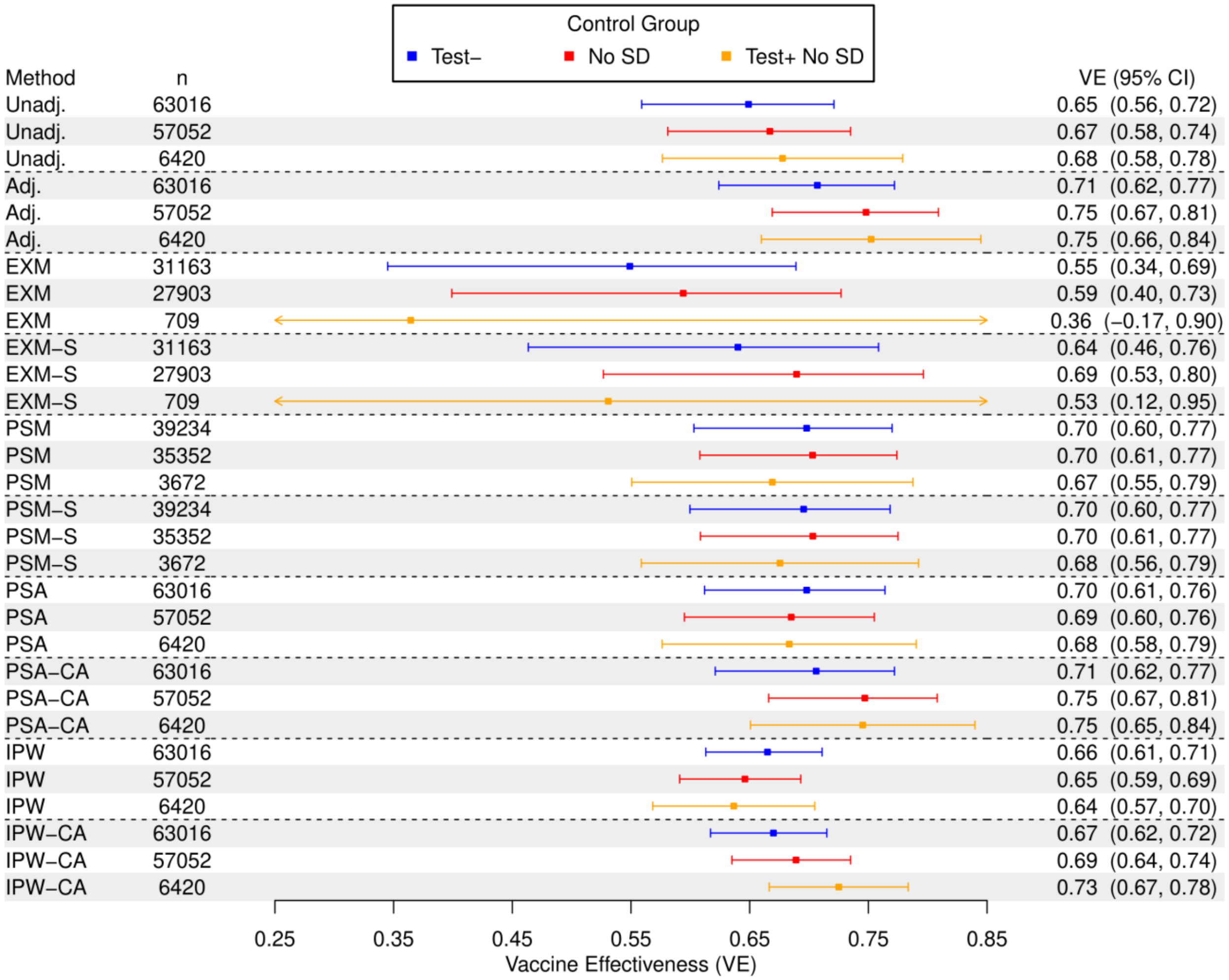

Fig. S17. **Test-negative first booster/third dose up to 2 quarters since vaccination vs full vaccination/second dose VE against severe disease**. We apply 3 different choices of control group and various matching or propensity score designs during a study period of October 1, 2021 to December 31, 2022.

Control Groups: (1) Test-, test-negatives; (2) No SD, no severe disease; (3) Test+ No SD, test-positives without severe disease.

Abbreviations: Unadj., unadjusted; Adj., adjusted for covariates; EXM, exact matching; EXM-S, exact matching accounting for matching strata in conditional logistic regression; PSM, propensity score matching with a 0.2 caliper; PSM-S, propensity score matching with a 0.2 caliper accounting for matching strata in conditional logistic regression; PSA; adjustment for propensity score; PSA-CA, adjustment for propensity score and for covariates; IPW, inverse probability weighting of propensity score; IPW-CA, inverse probability weighting of propensity score also adjusting for covariates.

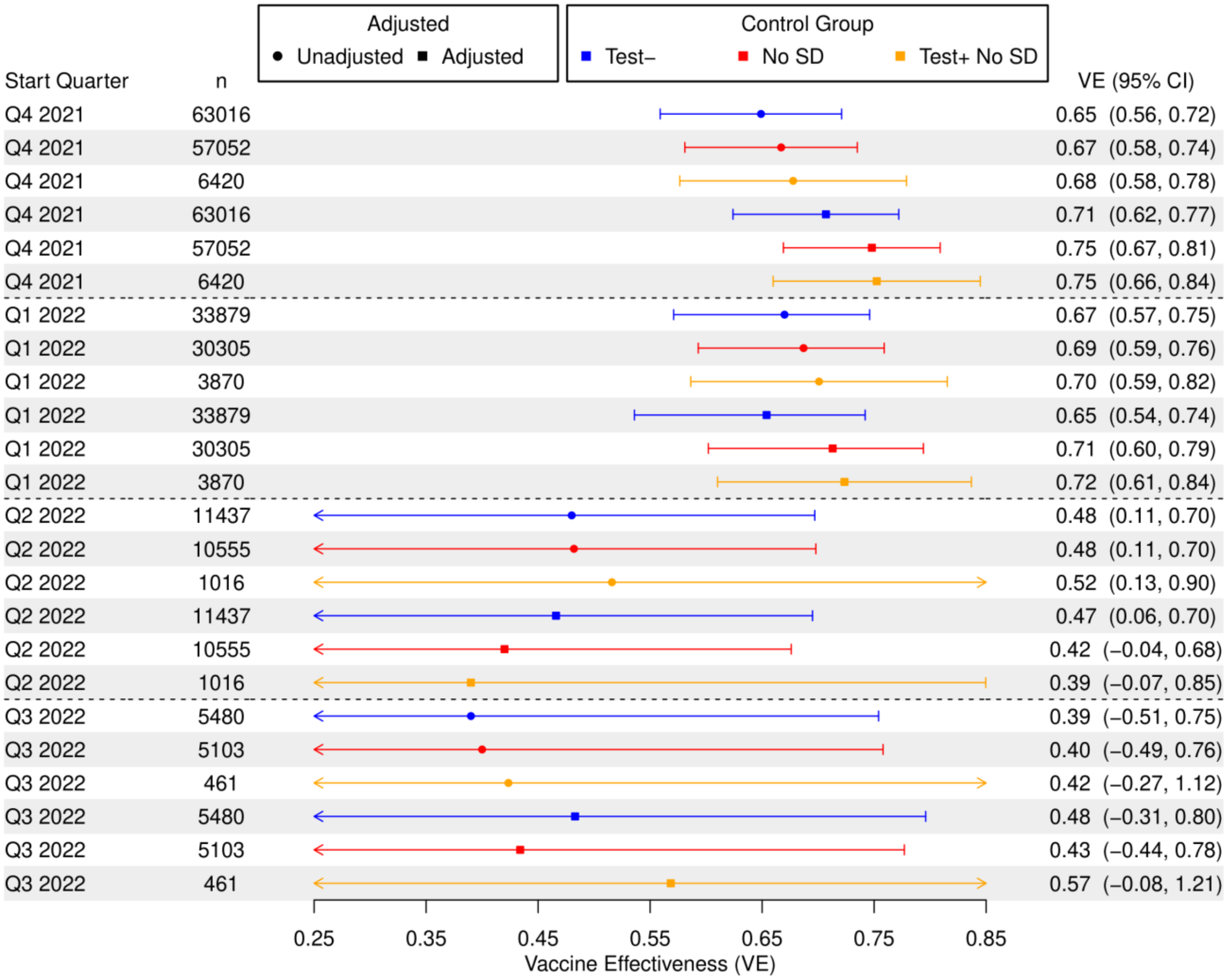

Fig. S18. Test-negative first booster/third dose up to 2 quarters since vaccination vs full vaccination/second dose VE against severe disease, with various study start (index) dates. We also apply 3 different choices of control group and use an end date of Dec 31, 2022. Control Groups: (1) Test-, test-negatives; (2) No SD, no severe disease; (3) Test+ No SD, test-positives without severe disease.

One or More Boosters vs. Primary Series

Cohort Infection – One or More Boosters vs. Primary Series

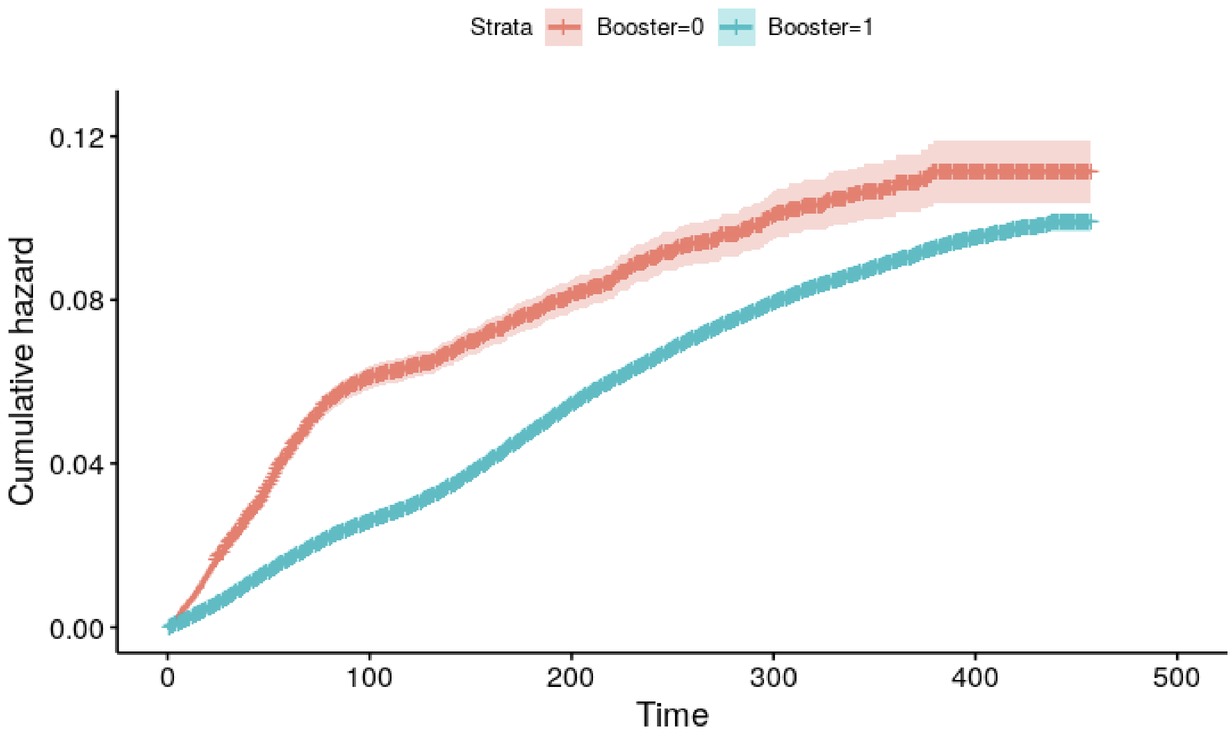

Fig. S19. **Kaplan-Meier curve for cohort boosted (3 or more doses) vs full vaccination/second dose VE against infection.** These curves describe a study period of October 1, 2021 to December 31, 2022.

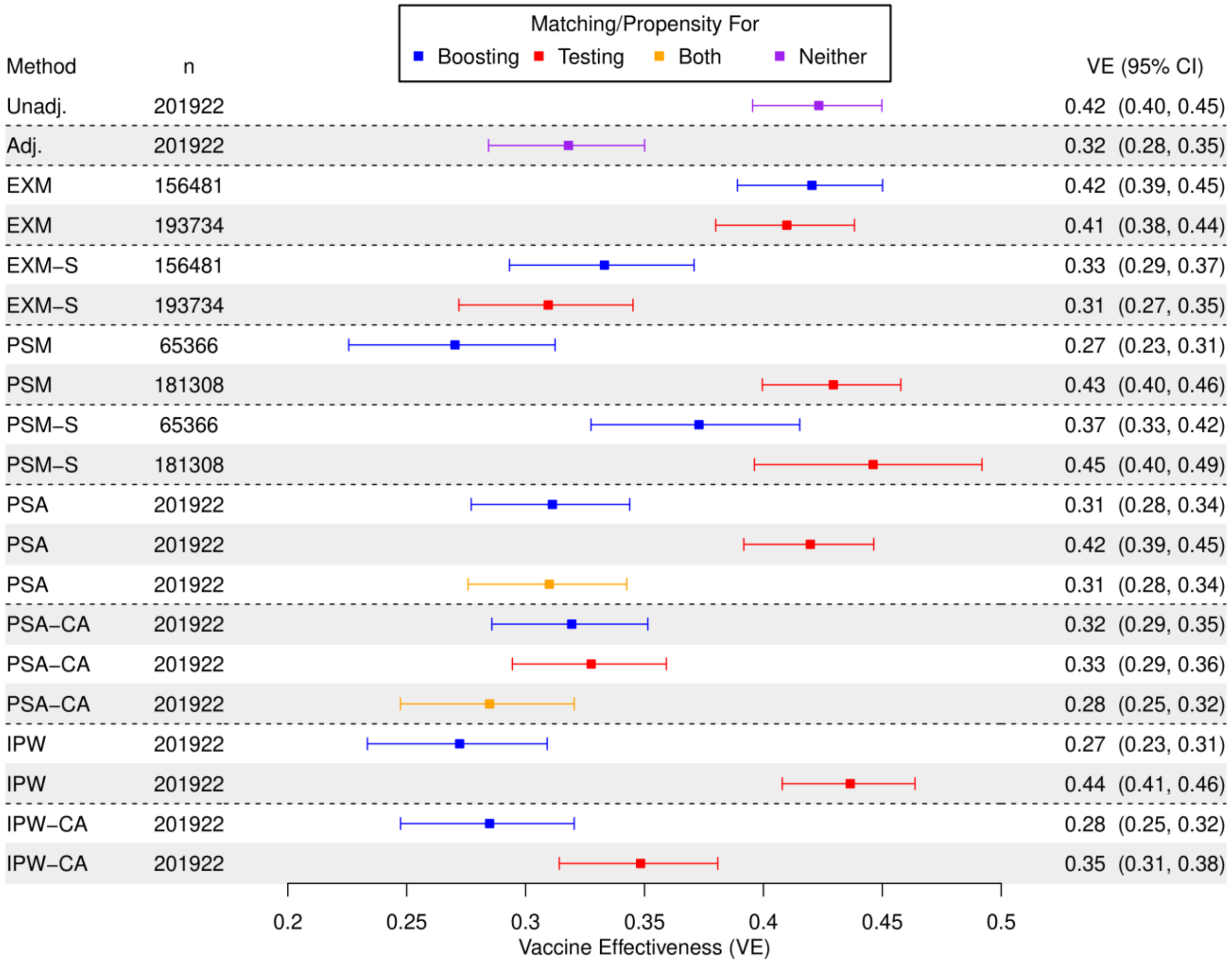

Fig. S20. **Cohort boosted (3 or more doses) vs full vaccination/second dose VE against infection**. We apply various matching designs and use a study period of October 1, 2021 to December 31, 2022.

Abbreviations: Unadj., unadjusted; Adj., adjusted for covariates; EXM, exact matching; EXM-S, exact matching with matching strata stratification in Cox regression; PSM, propensity score matching with a 0.2 caliper; PSM-S, propensity score matching with a 0.2 caliper and matching strata stratification in Cox regression; PSA; adjustment for propensity score; PSA-CA, adjustment for propensity score and for covariates; IPW, inverse probability weighting of propensity score; IPW-CA, inverse probability weighting of propensity score also adjusting for covariates.

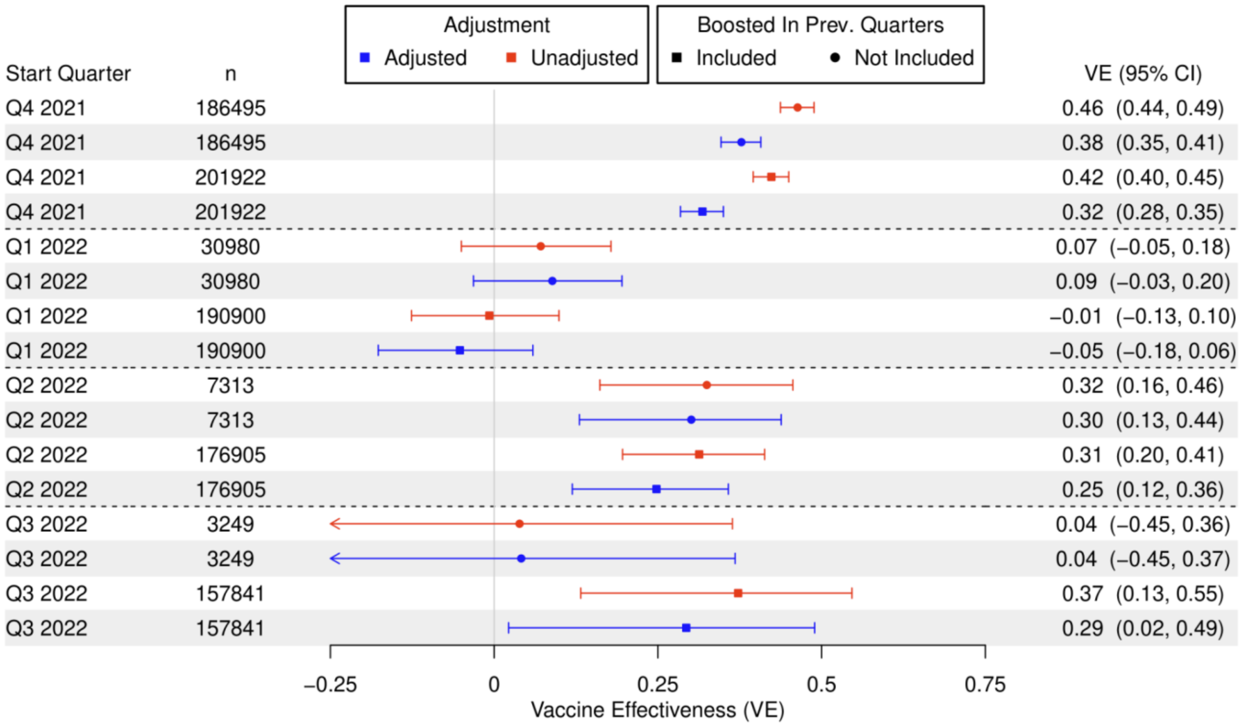

Fig. S21. Cohort boosted (3 or more doses) vs full vaccination/second dose VE against infection, with various study start (index) dates. All study periods use an end date of Dec 31, 2022.

Abbreviations: Boost in prior Q, if design includes in the boosted group only those boosted in the start quarter (N) or those boosted before or during the start quarter (Y).

Cohort Severe Disease – One or More Boosters vs. Primary Series

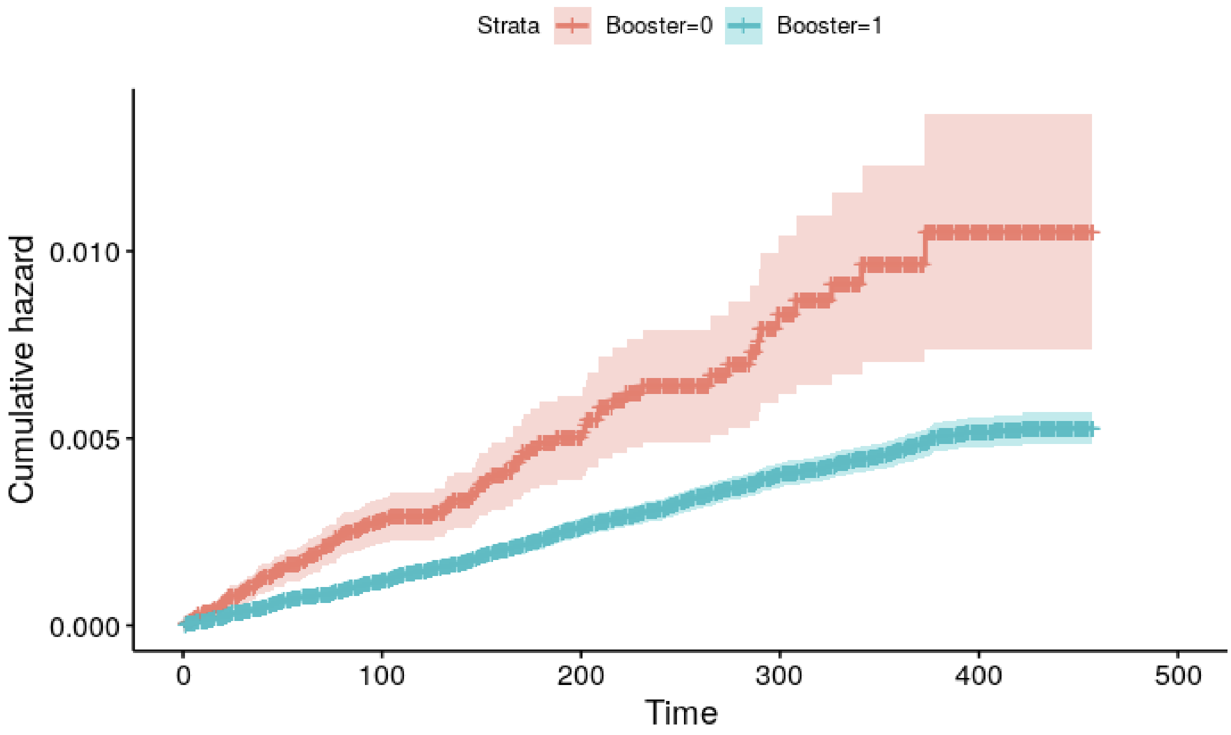

Fig. S22. Kaplan-Meier curve for cohort boosted (3 or more doses) vs full vaccination/second dose VE against severe disease. These curves describe a study period of October 1, 2021 to December 31, 2022.

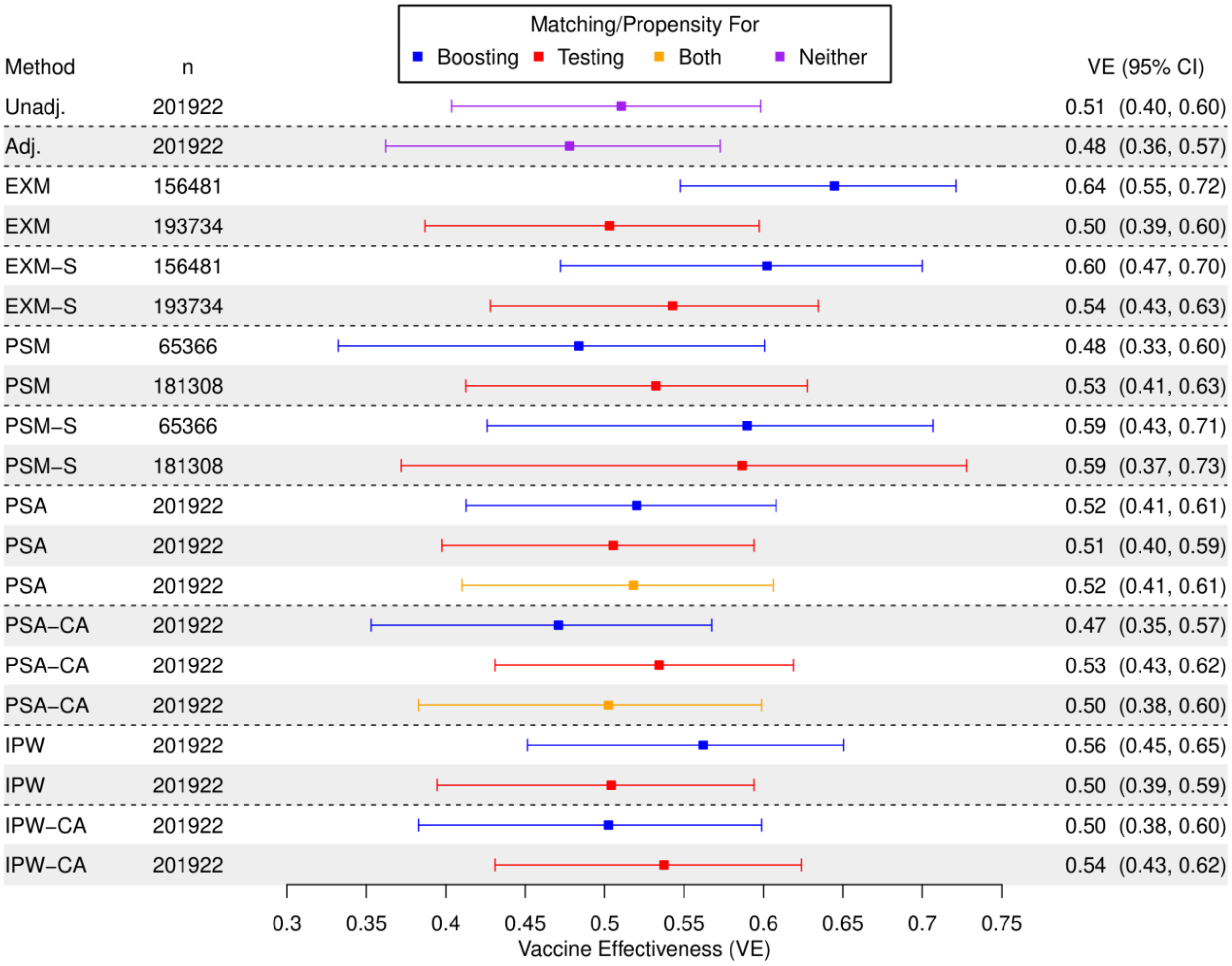

Fig. S23. Cohort boosted (3 or more doses) vs full vaccination/second dose VE against severe disease. We apply various matching or propensity score designs during a study period of October 1, 2021 to December 31, 2022.

Abbreviations: Unadj., unadjusted; Adj., adjusted for covariates; EXM, exact matching; EXM-S, exact matching with matching strata stratification in Cox regression; PSM, propensity score matching with a 0.2 caliper; PSM-S, propensity score matching with a 0.2 caliper and matching strata stratification in Cox regression; PSA; adjustment for propensity score; PSA-CA, adjustment for propensity score and for covariates; IPW, inverse probability weighting of propensity score; IPW-CA, inverse probability weighting of propensity score also adjusting for covariates.

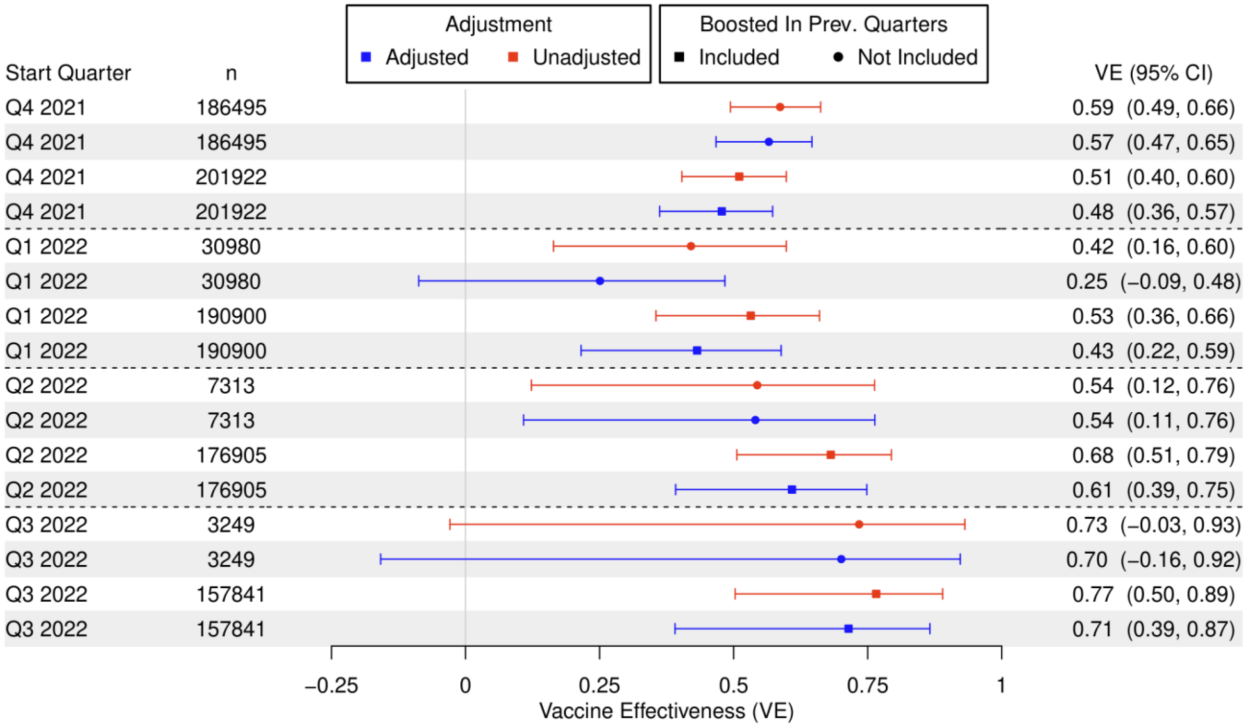

Fig. S24. Cohort boosted (3 or more doses) vs full vaccination/second dose VE against severe disease, with various study start (index) dates. We apply various matching designs and use a study end date of December 31, 2022.

Abbreviations: Boost in prior Q, if design includes in the second boosted group only those boosted in the start quarter (N) or those boosted before or during the start quarter (Y).

Test-Negative Infection – One or More Boosters vs. Primary Series

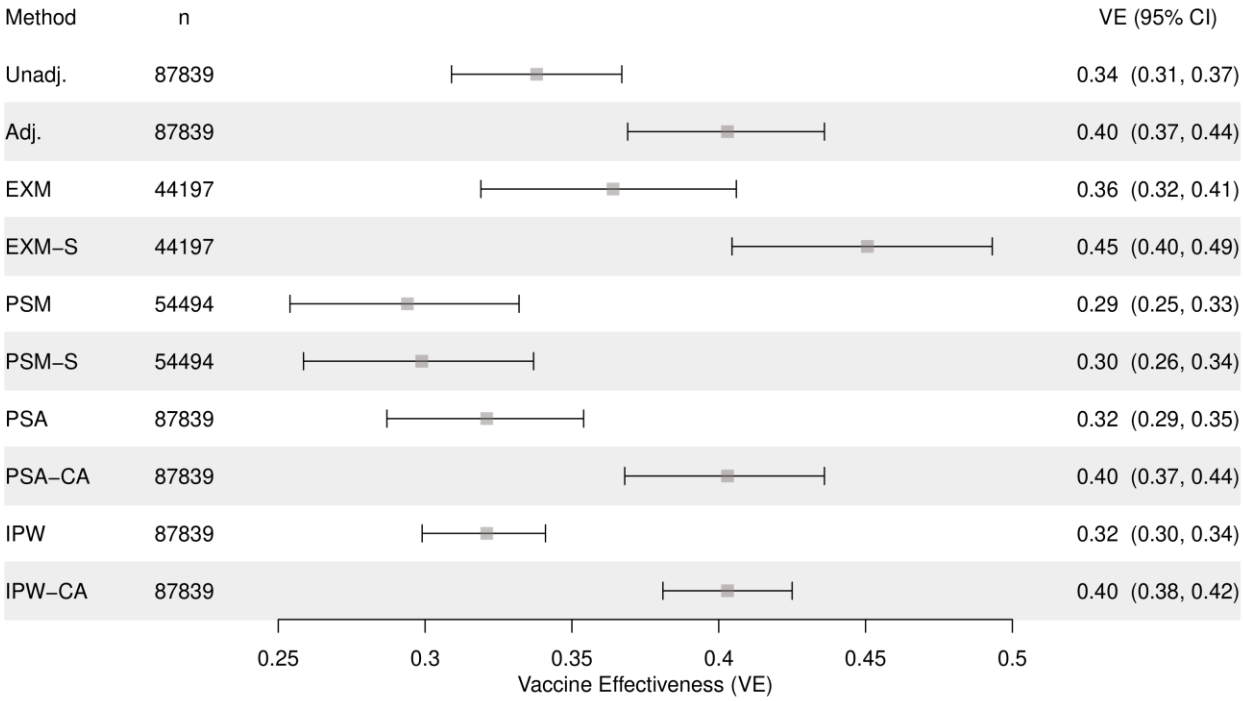

Fig. S25. **Test-negative boosted (3 or more doses) vs full vaccination/second dose VE against infection**. We apply various matching or propensity score designs and use a study period of October 1, 2021 to December 31, 2022.

Abbreviations: Unadj., unadjusted; Adj., adjusted for covariates; EXM, exact matching; EXM-S, exact matching accounting for matching strata in conditional logistic regression; PSM, propensity score matching with a 0.2 caliper; PSM-S, propensity score matching with a 0.2 caliper accounting for matching strata in conditional logistic regression; PSA; adjustment for propensity score; PSA-CA, adjustment for propensity score and for covariates; IPW, inverse probability weighting of propensity score; IPW-CA, inverse probability weighting of propensity score also adjusting for covariates.

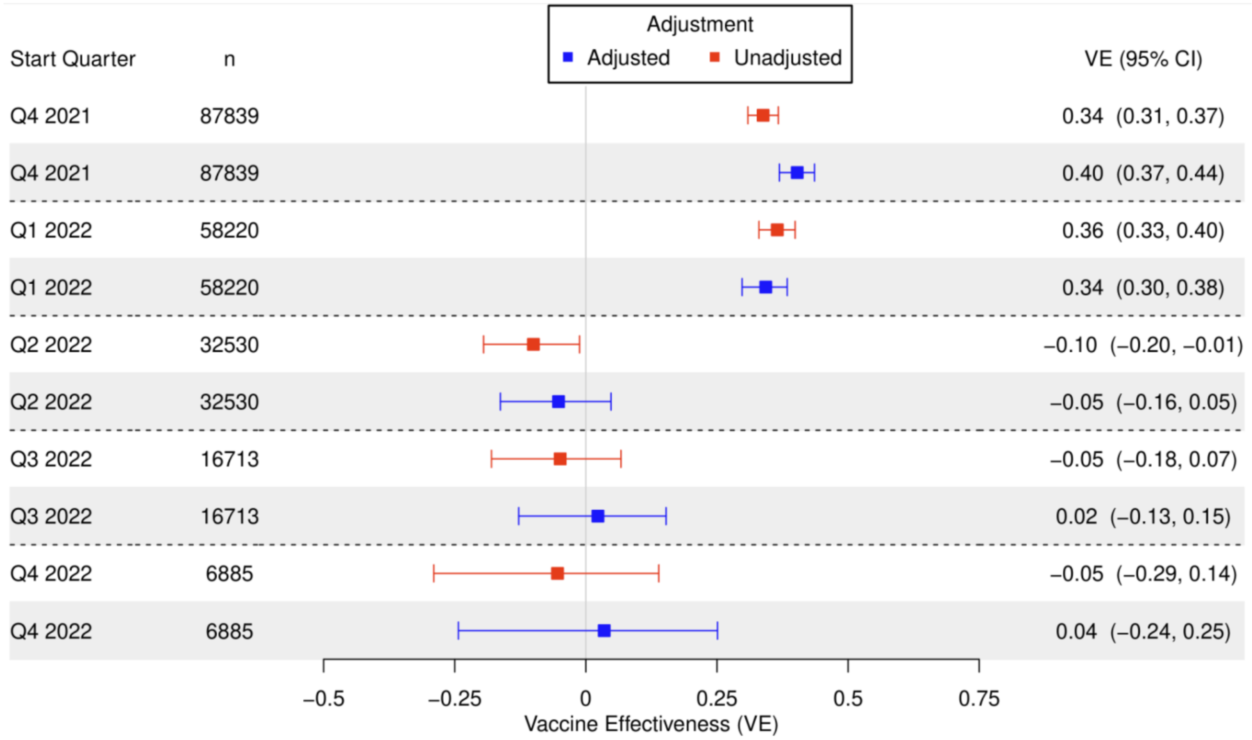

Fig. S26. Test-negative boosted (3 or more doses) vs full vaccination/second dose VE against infection, with various study start (index) dates. We use a study end date of December 31, 2022.

Test-Negative Severe Disease – One or More Boosters vs. Primary Series

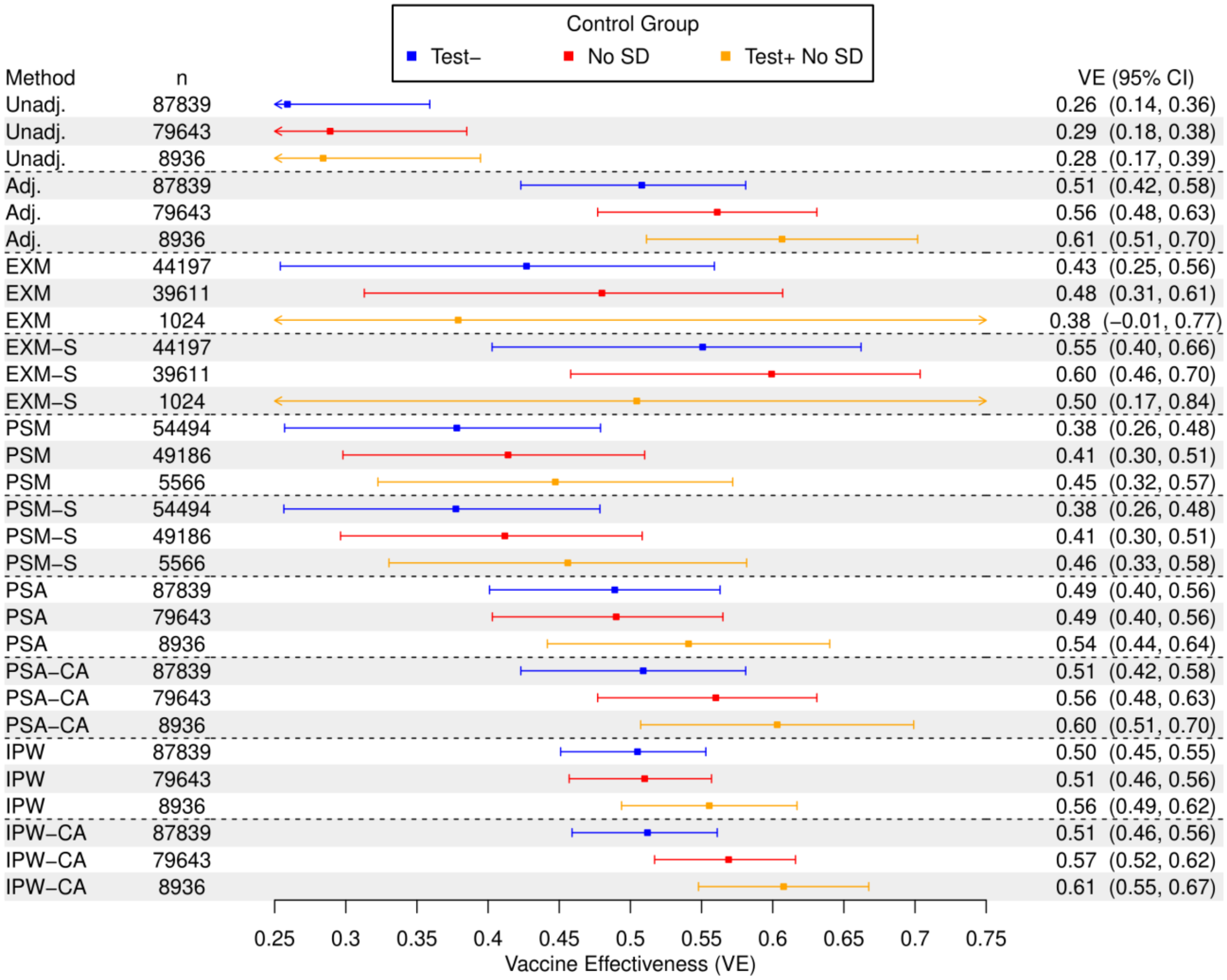

Fig. S27. **Test-negative boosted (3 or more doses) vs full vaccination/second dose VE against severe disease**. We apply various matching or propensity score designs and use a study period of October 1, 2021 to December 31, 2022.

Control Groups: (1) Test-, test-negatives; (2) No SD, no severe disease; (3) Test+ No SD, test-positives without severe disease.

Abbreviations: Unadj., unadjusted; Adj., adjusted for covariates; EXM, exact matching; EXM-S, exact matching accounting for matching strata in conditional logistic regression; PSM, propensity score matching with a 0.2 caliper; PSM-S, propensity score matching with a 0.2 caliper accounting for matching strata in conditional logistic regression; PSA; adjustment for propensity score; PSA-CA, adjustment for propensity score and for covariates; IPW, inverse probability weighting of propensity score; IPW-CA, inverse probability weighting of propensity score also adjusting for covariates.

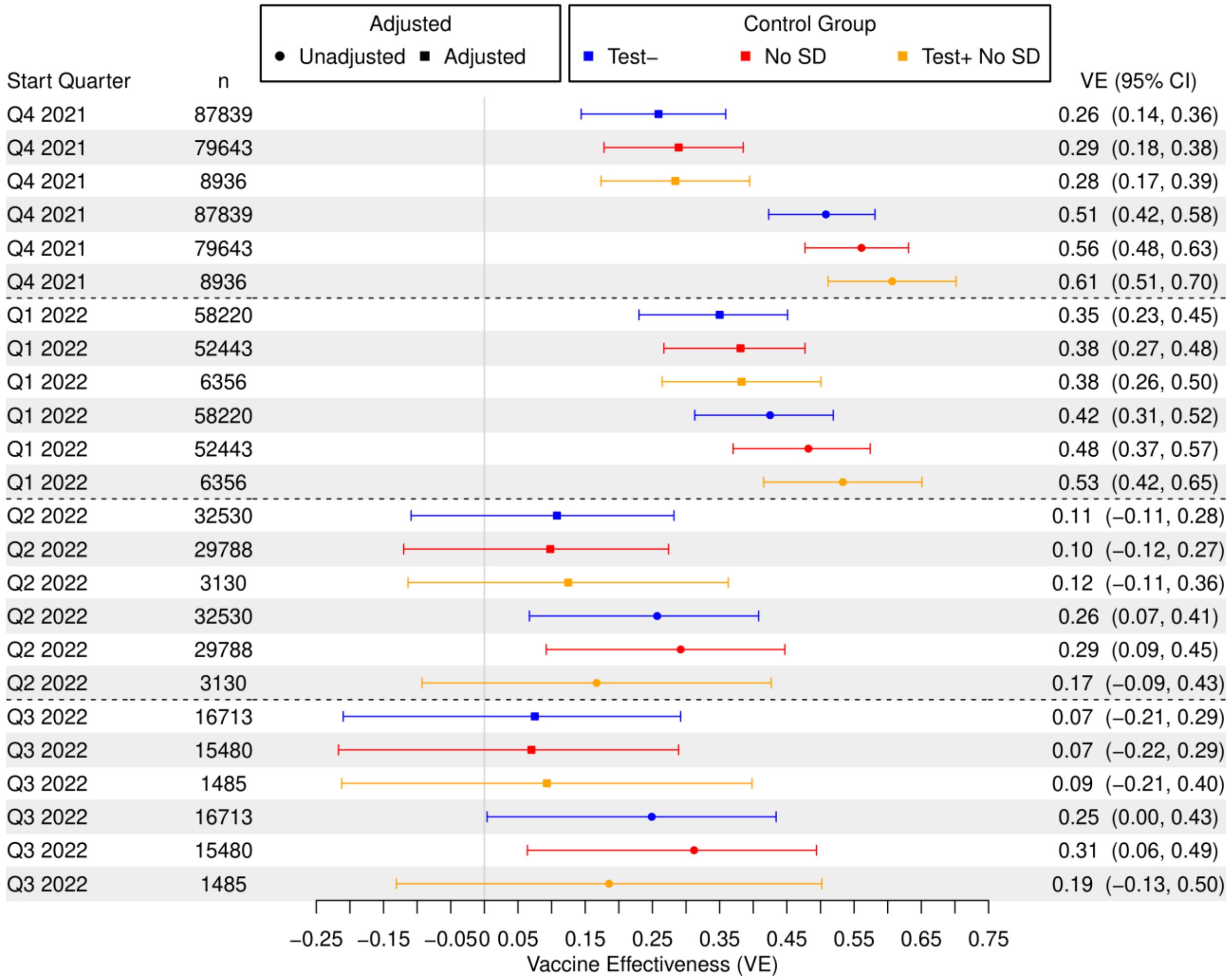

Fig. S28. Test-negative boosted (3 or more doses) vs full vaccination/second dose VE against severe disease, with various study start (index) dates. We use 3 choices of control group and a study end date of December 31, 2022.

Control Groups: (1) Test-, test-negatives; (2) No SD, no severe disease; (3) Test+ No SD, test-positives without severe disease.

Two or More Boosters vs. One Booster

Cohort Infection – Two or More Boosters vs. One Booster

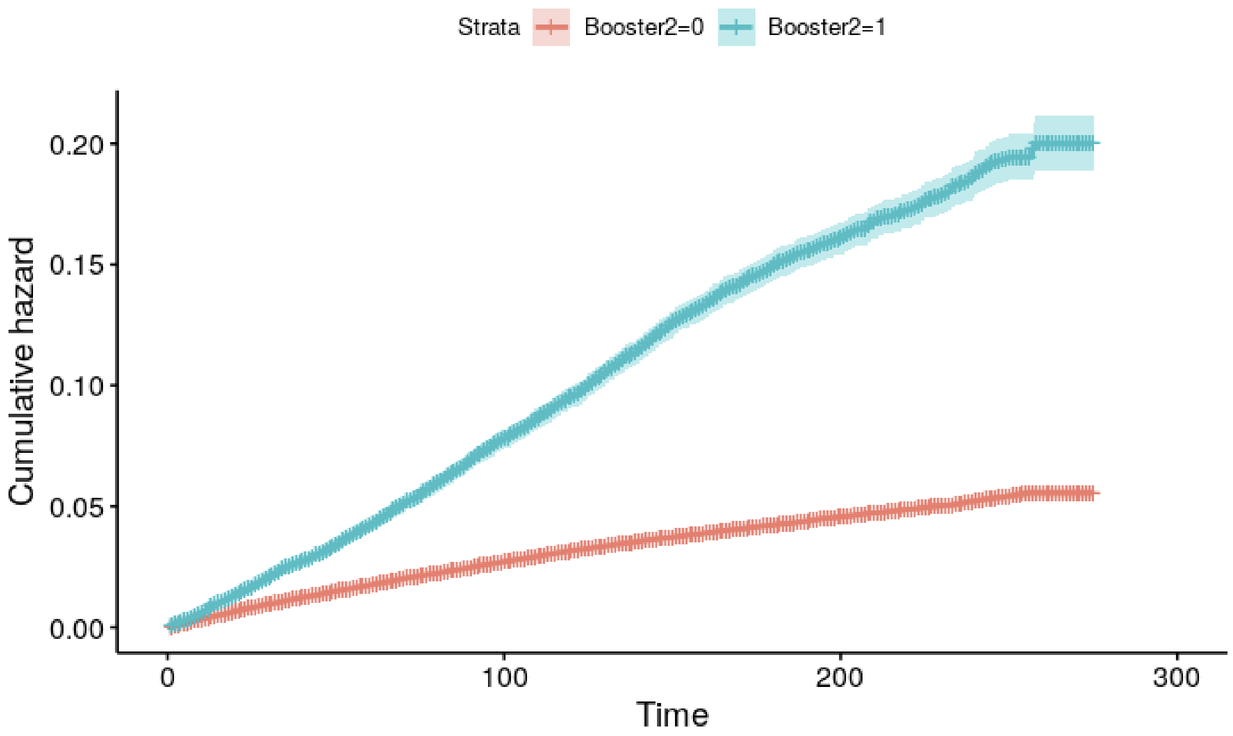

Fig. S29. Kaplan-Meier curve for cohort double boosted or more (4 or more doses) vs single boosted (3 doses) VE against infection. These curves describe a study period of April 1, 2022 to December 31, 2022.

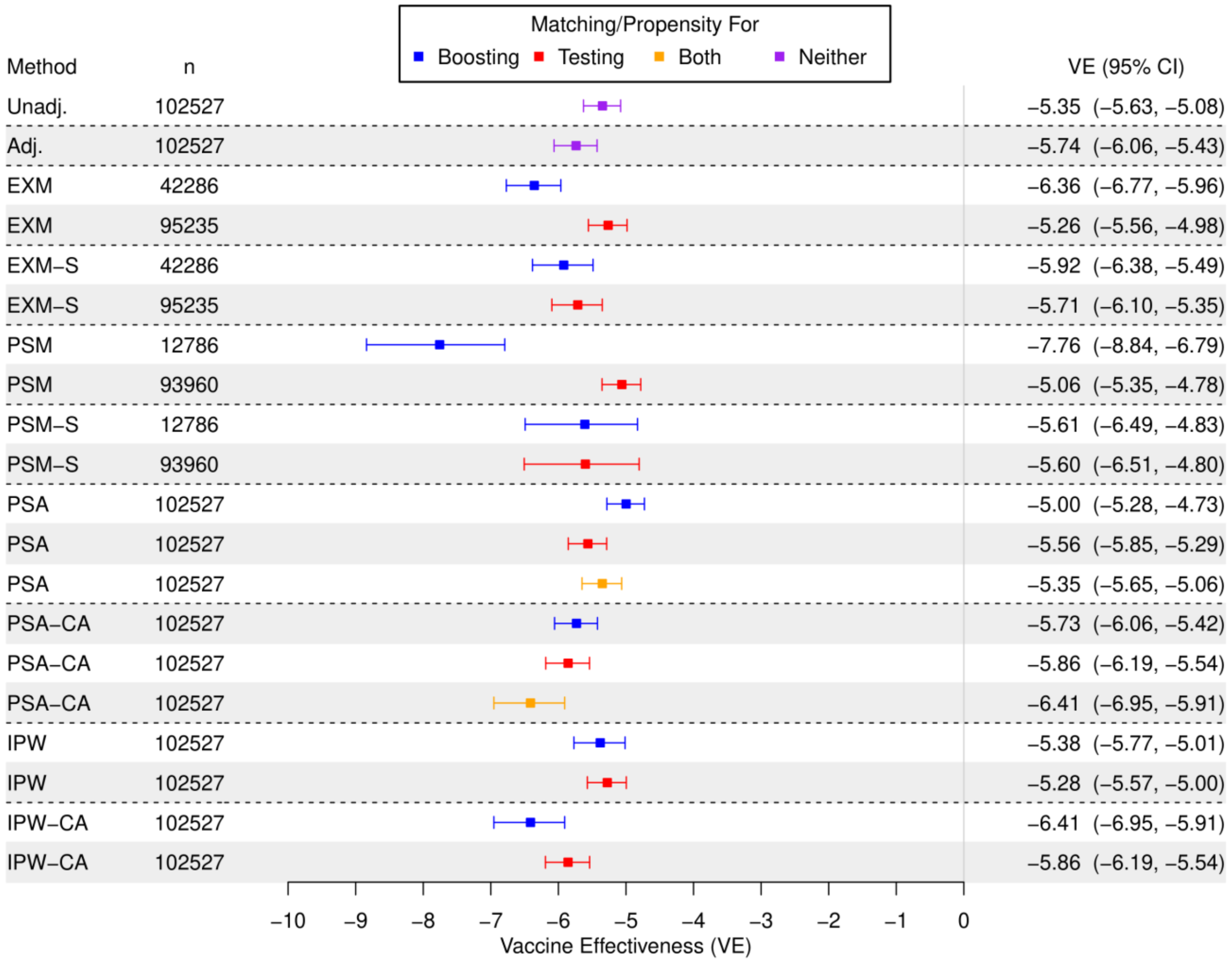

Fig. S30. **Cohort double boosted or more (4 or more doses) vs single boosted (3 doses) VE against infection**. We apply various matching designs and use a study period of April 1, 2022 to December 31, 2022.

Abbreviations: Unadj., unadjusted; Adj., adjusted for covariates; EXM, exact matching; EXM-S, exact matching with matching strata stratification in Cox regression; PSM, propensity score matching with a 0.2 caliper; PSM-S, propensity score matching with a 0.2 caliper and matching strata stratification in Cox regression; PSA; adjustment for propensity score; PSA-CA, adjustment for propensity score and for covariates; IPW, inverse probability weighting of propensity score; IPW-CA, inverse probability weighting of propensity score also adjusting for covariates.

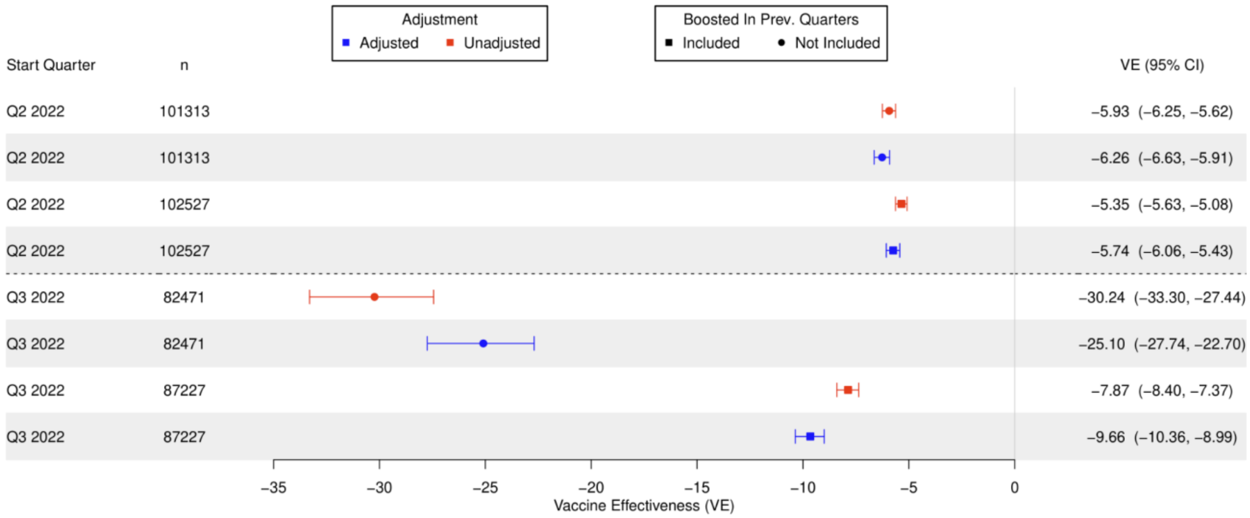

Fig. S31. **Cohort double boosted or more (4 or more doses) vs single boosted (3 doses) VE against infection, with various study start (index) dates**. We use a study end date of December 31, 2022 for all models.

Abbreviations: Boost in prior Q, if design includes in the second boosted group only those boosted in the start quarter (N) or those boosted before or during the start quarter (Y).

Cohort Severe Disease – Two or More Boosters vs. One Booster

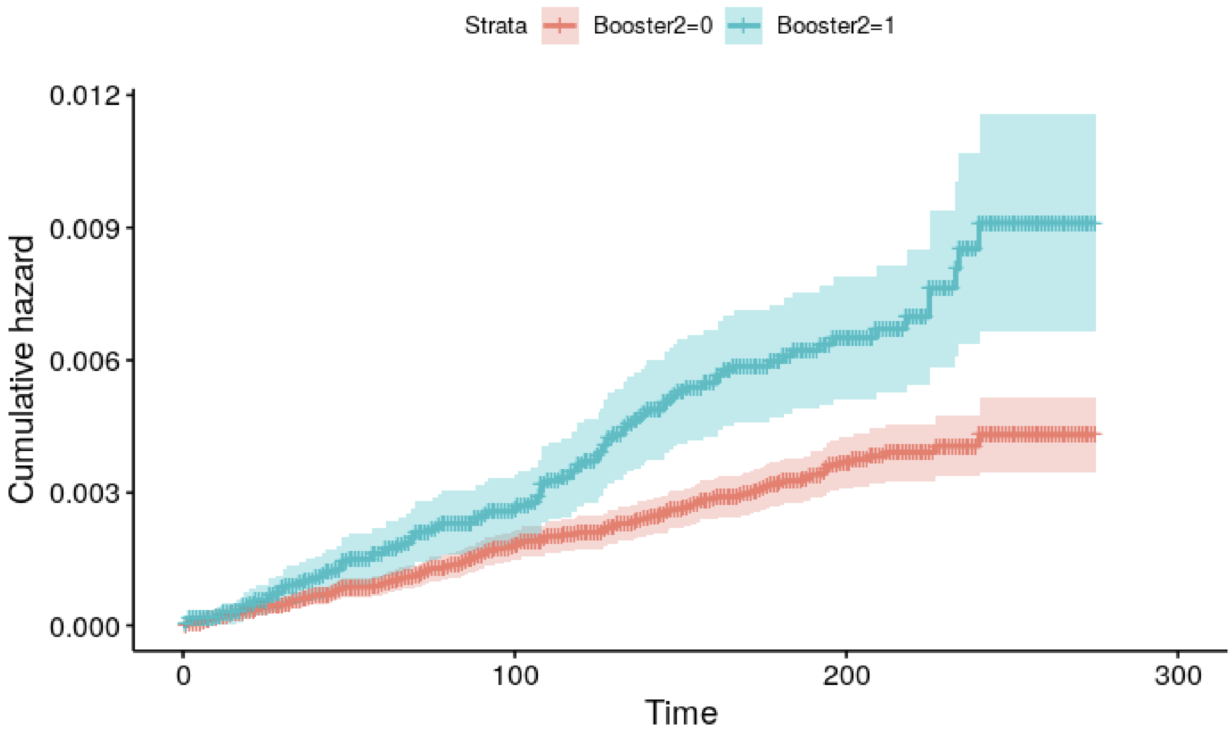

Fig. S32. Kaplan-Meier curves for cohort double boosted (4 or more doses) vs single boosted (3 doses) VE against severe disease. These curves describe a study period of April 1, 2022 to December 31, 2022.

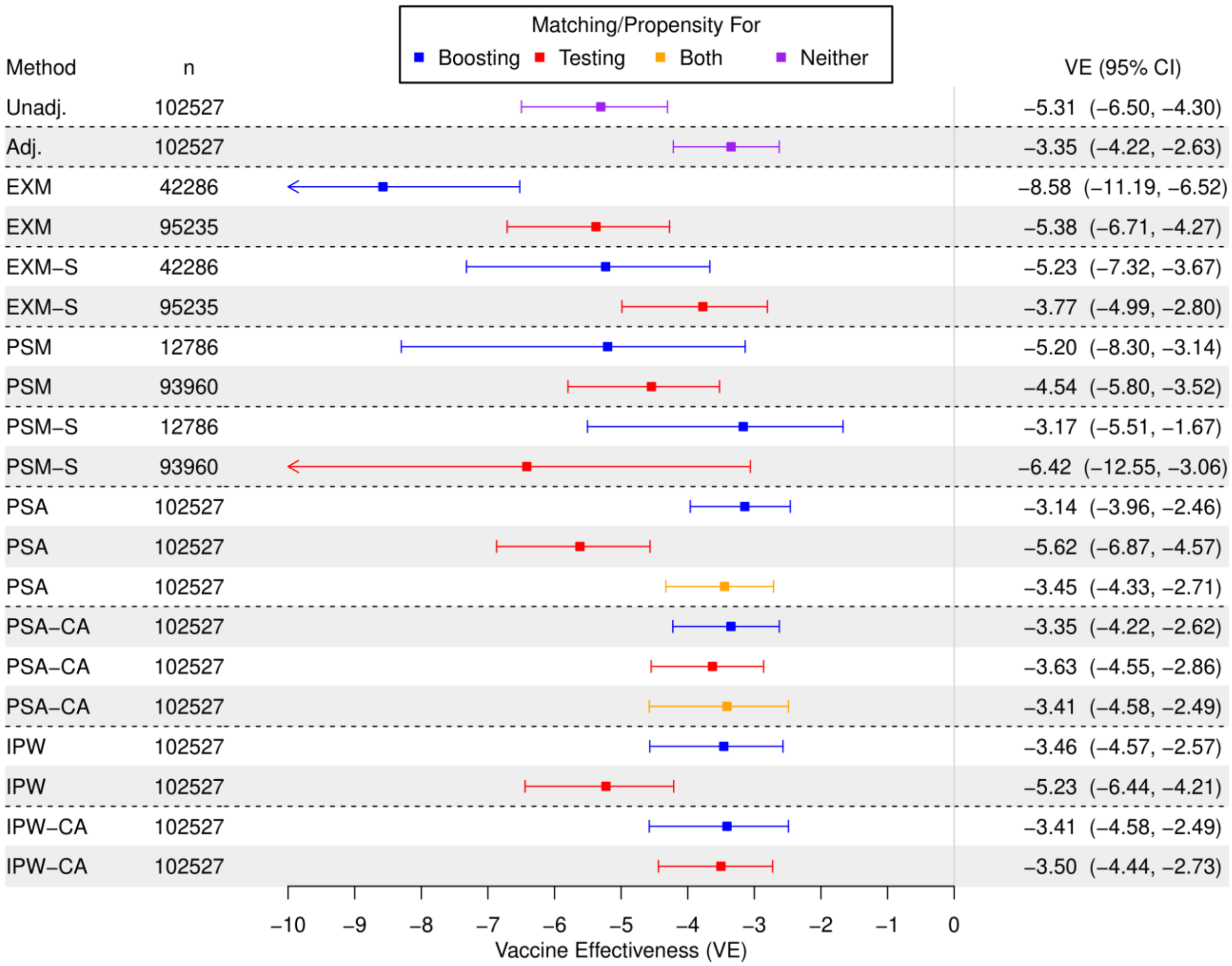

Fig. S33. **Cohort double boosted (4 or more doses) vs single boosted (3 doses) VE against severe disease**. We apply various matching or propensity score designs and use a study period of April 1, 2022 to December 31, 2022.

Abbreviations: Unadj., unadjusted; Adj., adjusted for covariates; EXM, exact matching; EXM-S, exact matching with matching strata stratification in Cox regression; PSM, propensity score matching with a 0.2 caliper; PSM-S, propensity score matching with a 0.2 caliper and matching strata stratification in Cox regression; PSA; adjustment for propensity score; PSA-CA, adjustment for propensity score and for covariates; IPW, inverse probability weighting of propensity score; IPW-CA, inverse probability weighting of propensity score also adjusting for covariates.

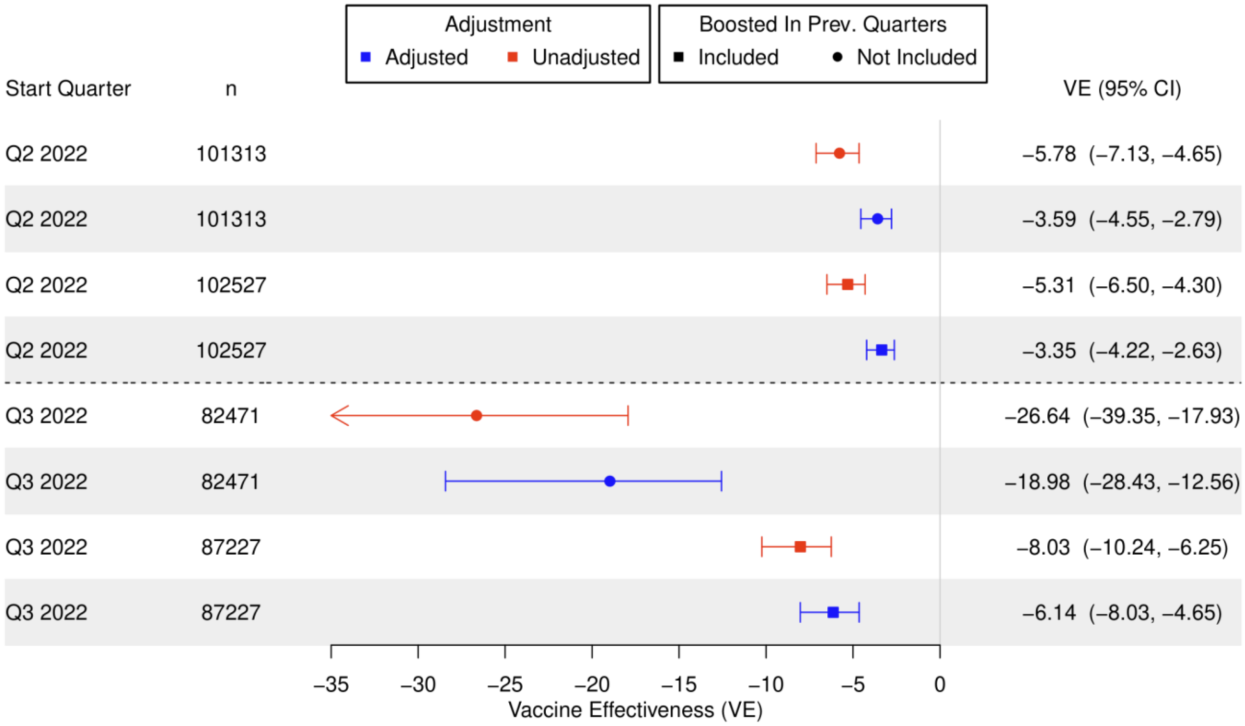

Fig. S34. Cohort double boosted (4 or more doses) vs single boosted (3 doses) VE against severe disease, with various study start (index) dates. We use 3 choices of control group and an end date of December 31, 2022.

Abbreviations: Boost in prior Q, if design includes in the second boosted group only those boosted in the start quarter (N) or those boosted before or during the start quarter (Y).

Test-Negative Infection – Two or More Boosters vs. One Booster

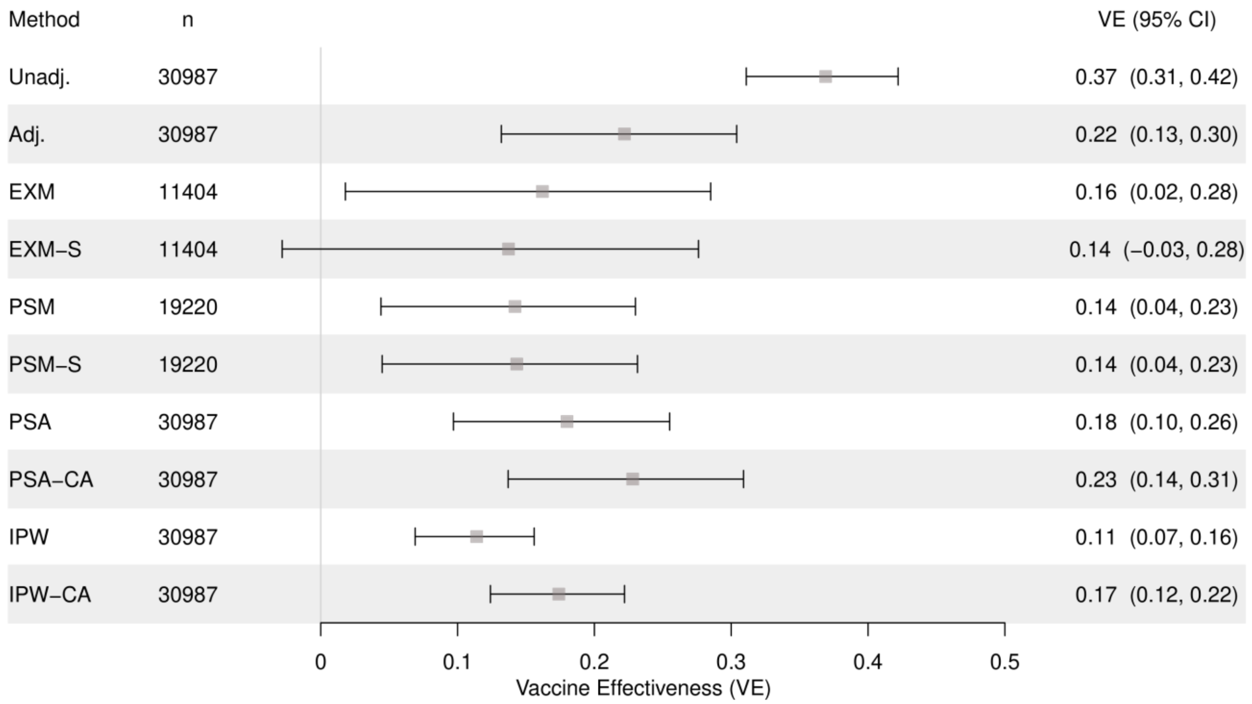

Fig. S35. Test-negative double boosted or more (4 or more doses) vs single boosted (3 doses) VE against infection. We apply various matching designs and use a study period of April 1, 2022 to December 31, 2022.

Abbreviations: Unadj., unadjusted; Adj., adjusted for covariates; EXM, exact matching; EXM-S, exact matching accounting for matching strata in conditional logistic regression; PSM, propensity score matching with a 0.2 caliper; PSM-S, propensity score matching with a 0.2 caliper accounting for matching strata in conditional logistic regression; PSA; adjustment for propensity score; PSA-CA, adjustment for propensity score and for covariates; IPW, inverse probability weighting of propensity score; IPW-CA, inverse probability weighting of propensity score also adjusting for covariates.

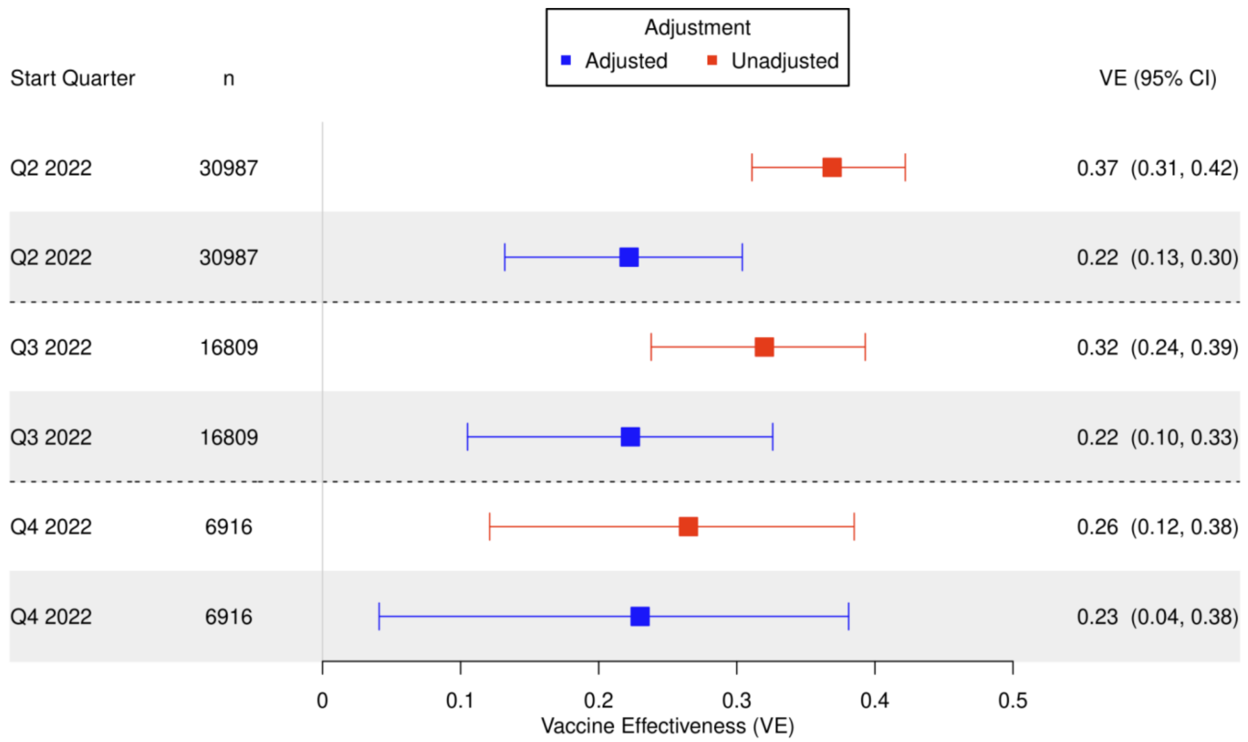

Fig. S36. Test-negative double boosted or more (4 or more doses) vs single boosted (3 doses) VE against infection, with various study start (index) dates. We use an end date of December 31, 2022 for all models.

Test-Negative Severe Disease – Two or More Boosters vs. One Booster

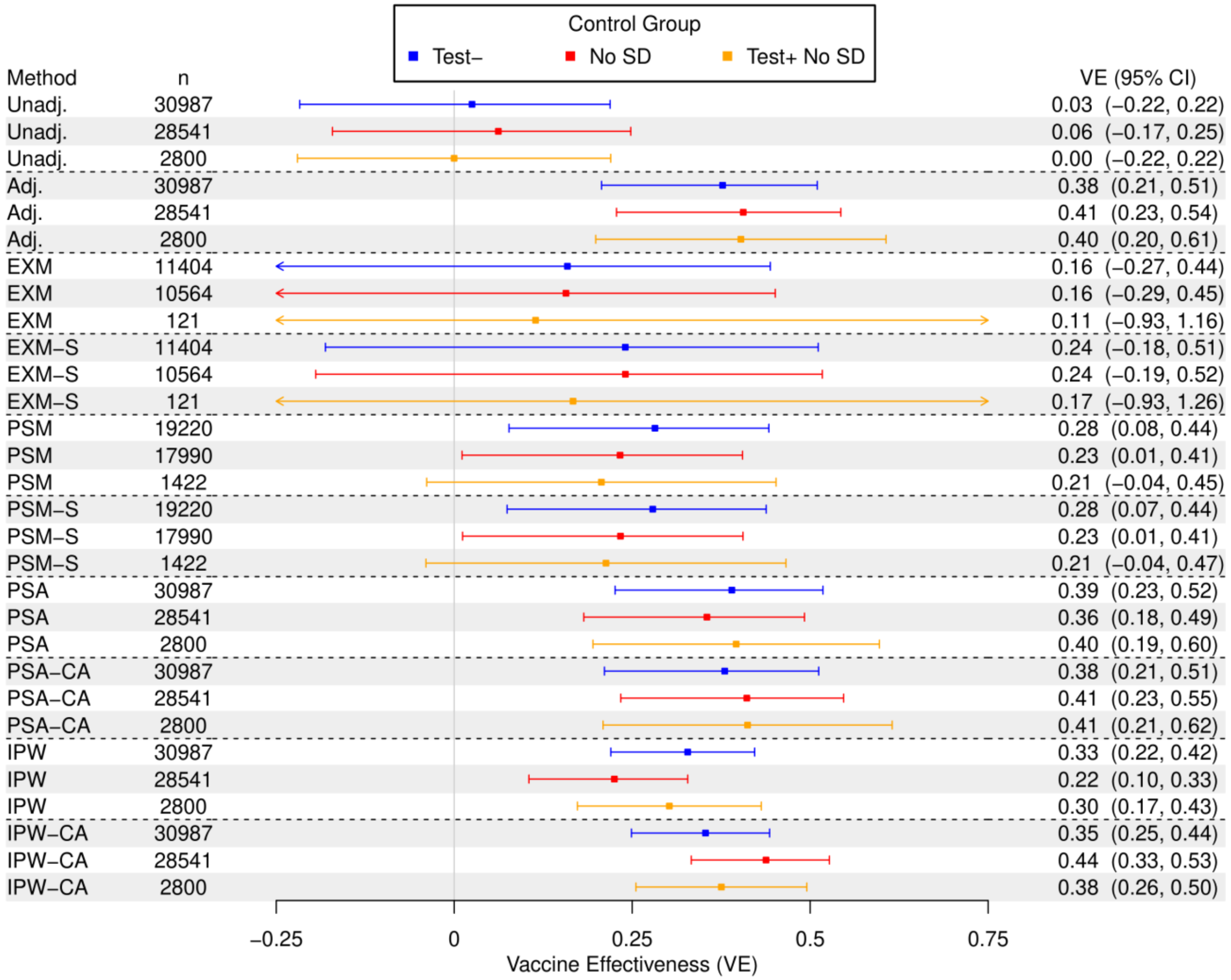

Fig. S37. **Test-negative double boosted (4 or more doses) vs single boosted (3 doses) VE against severe disease**. We apply various matching or propensity score designs during and use a study period of April 1, 2022 to December 31, 2022.

Control Groups: (1) Test-, test-negatives; (2) No SD, no severe disease; (3) Test+ No SD, test-positives without severe disease.

Abbreviations: Unadj., unadjusted; Adj., adjusted for covariates; EXM, exact matching; EXM-S, exact matching accounting for matching strata in conditional logistic regression; PSM, propensity score matching with a 0.2 caliper; PSM-S, propensity score matching with a 0.2 caliper accounting for matching strata in conditional logistic regression; PSA; adjustment for propensity score; PSA-CA, adjustment for propensity score and for covariates; IPW, inverse probability weighting of propensity score; IPW-CA, inverse probability weighting of propensity score also adjusting for covariates.

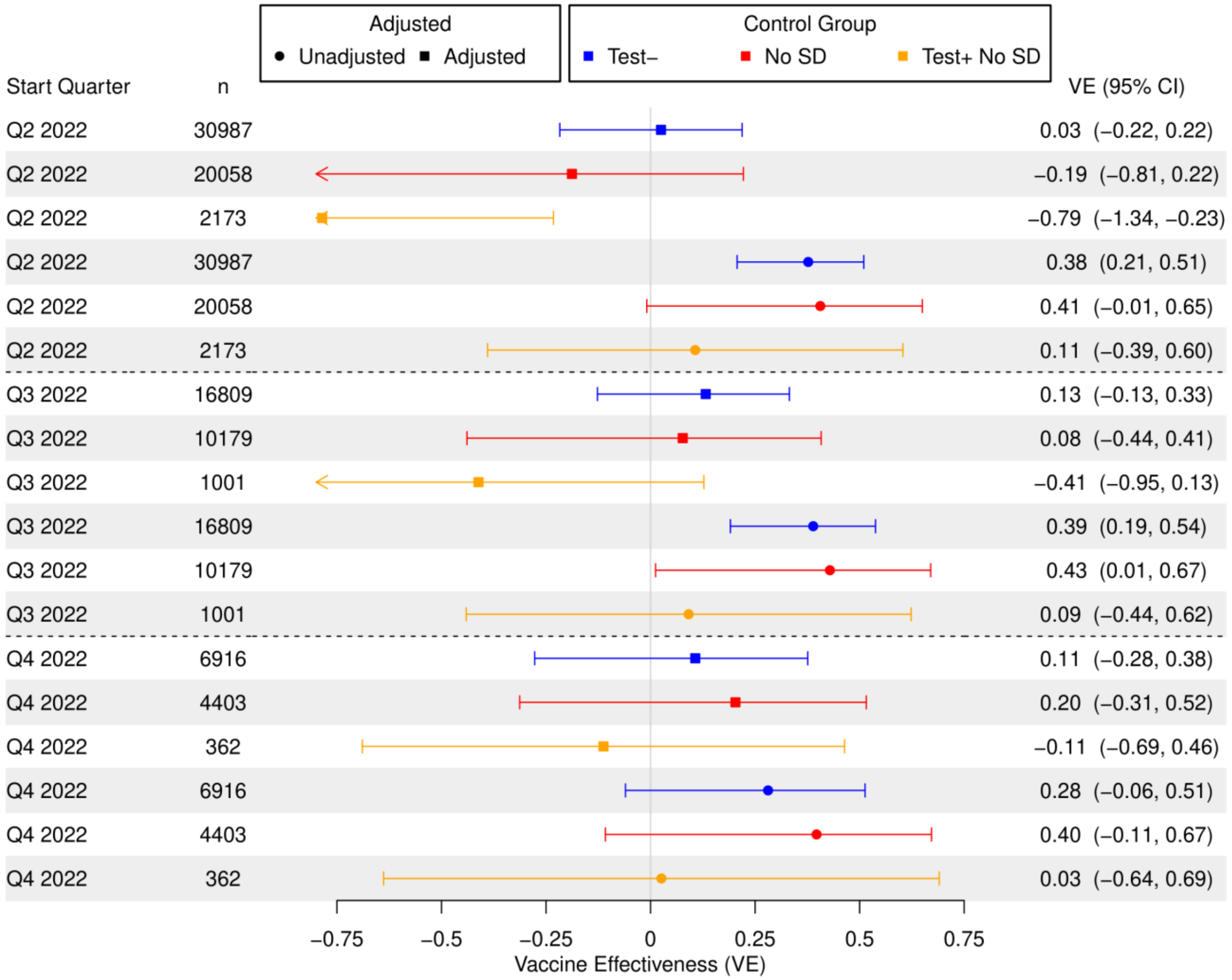

Fig. S38. Double boosted (4 or more doses) vs single boosted (3 doses) VE against severe disease, with various study start (index) dates. We use 3 choices of control group and use an end date of December 31, 2022 for all models.

Control Groups: (1) Test-, test-negatives; (2) No SD, no severe disease; (3) Test+ No SD, test-positives without severe disease.

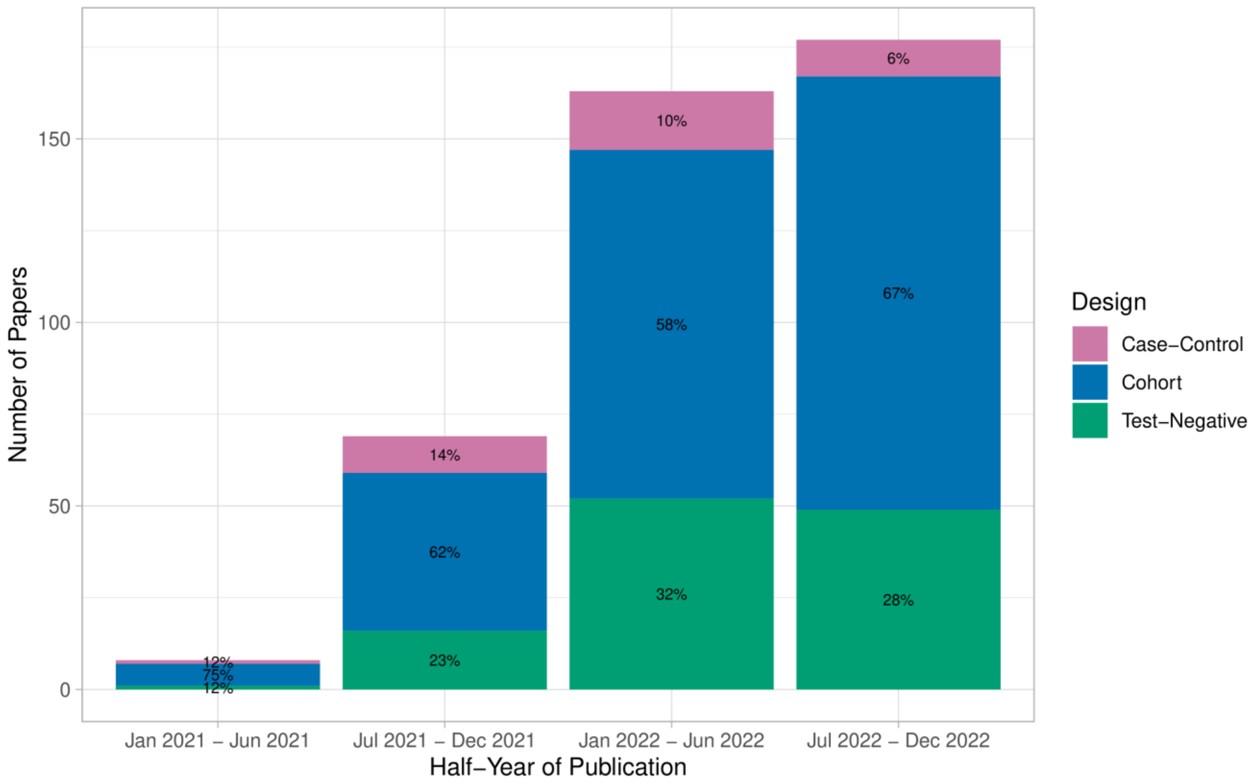

Fig. S39. **Summary of Literature Sweep of All COVID-19 VE Literature in each half of 2021 and 2022.** Number of published COVID-19 VE articles, preprints, or conference abstracts in each of 4 time periods: first half of 2021 (January 2021 – June 2021), second half of 2021 (July 2021 – December 2021), first half of 2022 (January 2022 – June 2022), and second half of 2022 (July 2022 – December 2022). Bars representing each time period are colored by class of study design, with the percentage of literature having each design in that time period displayed inside each shaded area. This is a companion figure to Fig. 1 in the main text, which does not divide 2021 into halves given the low volume of literature in the first half of 2021 (only 8 articles in the first half of 2021, compared to 69 in the second half).

Supplementary Tables

Table S1. Study designs of studies determining first booster VE compared to full vaccination and second booster VE compared to first booster. Designs are characterized as test-negative, other case-control, or cohort, and further subdivided based on their usage of matching.

(separate file)

Table S2. **Study characteristics for studies estimating first booster VE compared to primary series vaccination.**

(separate file)

Table S3. **Study characteristics for studies estimating second monovalent booster VE compared to first booster series.**

(separate file)

Table S4. **Study characteristics for studies estimating bivalent booster VE.**

(separate file)

Table S5. Characteristics of the first booster cohort study population, stratified on columns by booster status by the end of Q4 2021. For continuous variables, mean (standard deviation) is given, and for categorical variables, n (%) is given for each category. Some variables are given in both continuous and binned categorical form as they are used in different models.

(separate file)

Table S6. **Characteristics of the first booster test-negative study population, stratified on columns by booster status.** For continuous variables, mean (standard deviation) is given, and for categorical variables, n (%) is given for each category. Some variables are given in both continuous and binned categorical form as they are used in different models. Note that the number of observations in the overall column adds up to more than in each of the three booster status-stratified columns, as individuals were allowed to contribute tests at different booster statuses at different times.

(separate file)

Table S7. **Characteristics of the second booster secondary analysis cohort, stratified on columns by second booster status by the end of Q2 2022**. For continuous variables, mean (standard deviation) is given, and for categorical variables, n (%) is given for each category. Some variables are given in both continuous and binned categorical form as they are used in different models.

(separate file)

Table S8. **Characteristics of the full cohort, stratified on columns by booster status at any time since Q4 2021.** An individual may contribute to multiple columns. For continuous variables, mean (standard deviation) is given, and for categorical variables, n (%) is given for each category. Some variables are given in both continuous and binned categorical form as they are used in different models.

(separate file)

Table S9. **Summary of propensity score models.** We include coefficient values and associated 95% confidence intervals and p-values.

(separate file)

Table S10. Presence of each class of method in the literature. This table characterizes each of the classes of designs in our MM data analysis as common in the literature, rare in the literature, or proposed in our analysis.

(separate file)

Table S11: **Matching and propensity score methods used in the Michigan Medicine data analysis**. *X* used in the left side of equations refers to the full set of variables given on the right side.

(separate file)

Table S12. 2x2 Table of severe disease versus test positivity.

(separate file)

Table S13. Choices of control group for test-negative extension to severe disease. Choices of control groups for the extension of a test-negative study to severe disease outcomes as a case-control, expressed in terms of the entries of the 2x2 Table S12.

(separate file)

REFERENCES

1. D. L. Jeanmaire, R. P. Van Duyne, Surface raman spectroelectrochemistry: Part I. Heterocyclic, aromatic, and aliphatic amines adsorbed on the anodized silver electrode *Electrochemistry* **84**, 1–20 (1977).

2. A. Otto, I. Mrozek, H. Grabhorn, W. Akemann, Surface-enhanced Raman scattering. *J. Phys. Condens. Matter* **4**, 1143–1212 (1992).

3. R. Pilot, R. Signorini, C. Durante, L. Orian, M. Bhamidipati, L. Fabris, A review on surface-enhanced Raman scattering. *Biosensors* **9**, 57 (2019).

4. M. Moskovits, Surface-enhanced Raman spectroscopy: A brief retrospective. *J. Raman Spectros.* **36**, 485–496 (2005).

5. K. A. Willets, R. P. Van Duyne, Localized surface plasmon resonance spectroscopy and sensing. *Annu. Rev. Phys. Chem.* **58**, 267–297 (2007).

6. P. Roelli, C. Galland, N. Piro, T. J. Kippenberg, Molecular cavity optomechanics as a theory of plasmon-enhanced Raman scattering. *Nat. Nanotechnol.* **11**, 164–169 (2016).

7. R. Esteban, J. J. Baumberg, J. Aizpurua, Molecular optomechanics approach to surface-enhanced Raman scattering. *Acc. Chem. Res.* **55**, 1889–1899 (2022).

8. M. K. Schmidt, R. Esteban, F. Benz, J. J. Baumberg, J. Aizpurua, Linking classical and molecular optomechanics descriptions of SERS. *Faraday Discuss.* **205**, 31–65 (2017).

9. F. Benz, M. K. Schmidt, A. Dreismann, R. Chikkaraddy, Y. Zhang, A. Demetriadou, C. Carnegie, H. Ohadi, B. De Nijs, R. Esteban, J. Aizpurua, J. J. Baumberg, Single-molecule optomechanics in "picocavities". *Science* **354**, 726–729 (2016).

10. A. Lombardi, M. K. Schmidt, L. Weller, W. M. Deacon, F. Benz, B. de Nijs, J. Aizpurua, J. J. Baumberg, Pulsed molecular optomechanics in plasmonic nanocavities: From nonlinear vibrational instabilities to bond-breaking. *Phys. Rev. X* **8**, 011016 (2018).

11. Y. Xu, H. Hu, W. Chen, P. Suo, Y. Zhang, S. Zhang, H. Xu, Phononic cavity optomechanics of atomically thin crystal in plasmonic nanocavity. *ACS Nano* **16**, 12711–12719 (2022).

12 W. Chen, P. Roelli, H. Hu, S. Verlekar, S. P. Amirtharaj, A. I. Barreda, T. J. Kippenberg, M. Kovylina, E. Verhagen, A. Martínez, C. Galland, Continuous-wave frequency upconversion with a molecular optomechanical nanocavity. *Science* **374**, 1264–1267 (2021).

13. A. Xomalis, X. Zheng, R. Chikkaraddy, Z. Koczor-Benda, E. Miele, E. Rosta, G. A. E. Vandenbosch, A. Martínez, J. J. Baumberg, Detecting mid-infrared light by molecular frequency upconversion in dual-wavelength nanoantennas. *Science* **374**, 1268–1271 (2021).

14. M. Aspelmeyer, T. J. Kippenberg, F. Marquardt, Cavity optomechanics. *Rev. Mod. Phys.* **86**, 1391–1452 (2014).

15. M. K. Dezfouli, R. Gordon, S. Hughes, Molecular optomechanics in the anharmonic cavity-QED regime using hybrid metal-dielectric cavity modes. *ACS Photonics* **6**, 1400–1408 (2019).

16. I. Shlesinger, K. G. Cognée, E. Verhagen, A. F. Koenderink, Integrated molecular optomechanics with hybrid dielectric-metallic resonators. *ACS Photonics* **8**, 3506–3516 (2021).

17. I. Shlesinger, I. M. Palstra, A. F. Koenderink, Integrated sideband-resolved sers with a dimer on a nanobeam hybrid. *Phys. Rev. Lett.* **130**, 016901 (2023).

18. S. I. Shopova, C. W. Blackledge, A. T. Rosenberger, Enhanced evanescent coupling to whispering-gallery modes due to gold nanorods grown on the microresonator surface. *Appl. Phys. B* **93**, 183–187 (2008).

19. F. De Angelis, M. Patrini, G. Das, I. Maksymov, M. Galli, L. Businaro, L. C. Andreani, E. Di Fabrizio, A hybrid plasmonic−photonic nanodevice for label-free detection of a few molecules. *Nano Lett.* **8**, 2321–2327 (2008).

20. H. M. Doeleman, E. Verhagen, A. F. Koenderink, Antenna-cavity hybrids: Matching polar opposites for Purcell enhancements at any linewidth. *ACS Photonics* **3**, 1943–1951 (2016).

21. B. Gurlek, V. Sandoghdar, D. Martín-Cano, Manipulation of quenching in nanoantenna-emitter systems enabled by external detuned cavities: A path to enhance strong-coupling. *ACS Photonics* **5**, 456–461 (2018).

22. M. K. Dezfouli, R. Gordon, S. Hughes, Modal theory of modified spontaneous emission of a quantum emitter in a hybrid plasmonic photonic-crystal cavity system. *Phys. Rev. A* **95**, 013846 (2017).

23. G. P. Acuna, F. M. Möller, P. Holzmeister, S. Beater, B. Lalkens, P. Tinnefeld, Fluorescence enhancement at docking sites of DNA-directed self-assembled nanoantennas. *Science* **338**, 506–510 (2012).

24. J. J. Baumberg, J. Aizpurua, M. H. Mikkelsen, D. R. Smith, Extreme nanophotonics from ultrathin metallic gaps. *Nat. Mater.* **18**, 668–678 (2019).

25. A. Xomalis, R. Chikkaraddy, E. Oksenberg, I. Shlesinger, J. Huang, E. C. Garnett, A. F. Koenderink, J. J. Baumberg, Controlling optically driven atomic migration using crystal-facet control in plasmonic nanocavities. *ACS Nano* **14**, 10562–10568 (2020).

26. R. J. Barbour, P. A. Dalgarno, A. Curran, K. M. Nowak, H. J. Baker, D. R. Hall, N. G. Stoltz, P. M. Petroff, R. J. Warburton, A tunable microcavity. *J. Appl. Phys.* **110**, 053107 (2011).

27. A. Moreau, C. Ciracì, J. J. Mock, R. T. Hill, Q. Wang, B. J. Wiley, A. Chilkoti, D. R. Smith, Controlled-reflectance surfaces with film-coupled colloidal nanoantennas. *Nature* **492**, 86–89 (2012).

28. H. Kelkar, D. Wang, D. Martín-Cano, B. Hoffmann, S. Christiansen, S. Götzinger, V. Sandoghdar, Sensing nanoparticles with a cantilever-based scannable optical cavity of low finesse and sub-λ^3 volume. *Phys. Rev. Appl.* **4**, 054010 (2015).

29. M. Mader, J. Reichel, T. W. Hänsch, D. Hunger, A scanning cavity microscope. *Nat. Commun.* **6**, 7249 (2015).

30. J. B. Lassiter, F. McGuire, J. J. Mock, C. Ciracì, R. T. Hill, B. J. Wiley, A. Chilkoti, D. R. Smith, Plasmonic waveguide modes of film-coupled metallic nanocubes. *Nano Lett.* **13**, 5866–5872 (2013).

31. R. Chikkaraddy, X. Zheng, F. Benz, L. J. Brooks, B. De Nijs, C. Carnegie, M.-E. Kleemann, J. Mertens, R. W. Bowman, G. A. E. Vandenbosch, V. V. Moshchalkov, J. J. Baumberg, How ultranarrow gap symmetries control plasmonic nanocavity modes: From cubes to spheres in the nanoparticle-on-mirror. *ACS Photonics* **4**, 469–475 (2017).

32. W. Yan, R. Faggiani, P. Lalanne, Rigorous modal analysis of plasmonic nanoresonators. *Phys. Rev. B* **97**, 205422 (2018).

33. P. Lalanne, W. Yan, K. Vynck, C. Sauvan, J.-P. Hugonin, Light interaction with photonic and plasmonic resonances. *Laser Photon. Rev.* **12**, 1700113 (2018).

34. J. Yang, J.-P. Hugonin, P. Lalanne, Near-to-far field transformations for radiative and guided waves. *ACS Photonics* **3**, 395–402 (2016).

35. R. Chikkaraddy, J. J. Baumberg, Accessing plasmonic hotspots using nanoparticle-on-foil constructs. *ACS Photonics* **8**, 2811–2817 (2021).

36. C. Sauvan, J. P. Hugonin, I. S. Maksymov, P. Lalanne, Theory of the spontaneous optical emission of nanosize photonic and plasmon resonators. *Phys. Rev. Lett.* **110**, 237401 (2013).

37. N. Thakkar, M. T. Rea, K. C. Smith, K. D. Heylman, S. C. Quillin, K. A. Knapper, E. H. Horak, D. J. Masiello, R. H. Goldsmith, Sculpting Fano resonances to control photonic-plasmonic hybridization. *Nano Lett.* **17**, 6927–6934 (2017).

38. H. M. Doeleman, C. D. Dieleman, C. Mennes, B. Ehrler, A. F. Koenderink, Observation of cooperative Purcell enhancements in antenna-cavity hybrids. *ACS Nano* **14**, 12027–12036 (2020).

39. A. G. Primo, N. C. Carvalho, C. M. Kersul, N. C. Frateschi, G. S. Wiederhecker, T. P. M. Alegre, Quasinormal-mode perturbation theory for dissipative and dispersive optomechanics. *Phys. Rev. Lett.* **125**, 233601 (2020).

40. A. F. Koenderink, M. Kafesaki, B. C. Buchler, V. Sandoghdar, Controlling the resonance of a photonic crystal microcavity by a near-field probe. *Phys. Rev. Lett.* **95**, 153904 (2005).

41. F. Ruesink, H. M. Doeleman, R. Hendrikx, A. F. Koenderink, E. Verhagen, Perturbing open cavities: Anomalous resonance frequency shifts in a hybrid cavity-nanoantenna system. *Phys. Rev. Lett.* **115**, 203904 (2015).

42. E. Oksenberg, I. Shlesinger, A. Xomalis, A. Baldi, J. J. Baumberg, A. F. Koenderink, E. C. Garnett, Energy-resolved plasmonic chemistry in individual nanoreactors. *Nat. Nanotechnol.* **16**, 1378–1385 (2021).

43. D. Hunger, T. Steinmetz, Y. Colombe, C. Deutsch, T. W. Hänsch, J. Reichel, A fiber Fabry-Perot cavity with high finesse. *New J. Phys.* **12**, 065038 (2010).

44. J. Mertens, M.-E. Kleemann, R. Chikkaraddy, P. Narang, J. J. Baumberg, How light is emitted by plasmonic metals. *Nano Lett.* **17**, 2568–2574 (2017).

45. P. Roelli, D. Martin-Cano, T. J. Kippenberg, C. Galland, Molecular platform for frequency upconversion at the single-photon level. *Phys. Rev. X* **10**, 031057 (2020).

46. K. Kneipp, Y. Wang, H. Kneipp, L. T. Perelman, I. Itzkan, R. R. Dasari, M. S. Feld, Single molecule detection using surface-enhanced raman scattering (sers). *Phys. Rev. Lett.* **78**, 1667–1670 (1997).

47. L. Li, T. Hutter, U. Steiner, S. Mahajan, Single molecule SERS and detection of biomolecules with a single gold nanoparticle on a mirror junction. *Analyst* **138**, 4574 (2013), 4578.

48. A. A. Clerk, F. Marquardt, K. Jacobs, Back-action evasion and squeezing of a mechanical resonator using a cavity detector. *New J. Phys.* **10**, 095010 (2008).

49. C. F. Ockeloen-Korppi, E. Damskägg, J.-M. Pirkkalainen, M. Asjad, A. A. Clerk, F. Massel, M. J. Woolley, M. A. Sillanpää, Stabilized entanglement of massive mechanical oscillators. *Nature* **556**, 478–482 (2018).

50. T. Hümmer, J. Noe, M. S. Hofmann, T. W. Hänsch, A. Högele, D. Hunger, Cavity-enhanced Raman microscopy of individual carbon nanotubes. *Nat. Commun.* **7**, 12155 (2016).

51. I. M. Palstra, H. M. Doeleman, A. F. Koenderink, Hybrid cavity-antenna systems for quantum optics outside the cryostat? *Nanophotonics* **8**, 1513–1531 (2019).

52. Y. Zhang, J. Aizpurua, R. Esteban, Optomechanical collective effects in surface-enhanced Raman scattering from many molecules. *ACS Photonics* **7**, 1676–1688 (2020).
